# Access to Isoquinolin-2(1*H*)-yl-acetamides and Isoindolin-2-yl-acetamides from a Common MCR Precursor

**DOI:** 10.1021/acs.joc.2c01905

**Published:** 2022-10-25

**Authors:** Xin Li, Qian Wang, Qiang Zheng, Katarzyna Kurpiewska, Justyna Kalinowska-Tluscik, Alexander Dömling

**Affiliations:** † Department of Drug Design, University of Groningen, A. Deusinglaan 1, 9713 AV Groningen, The Netherlands; ‡ Faculty of Chemistry, Department of Crystal Chemistry and Crystal, Physics, Biocrystallography Group, Jagiellonian University, Gronostajowa 2, 30-387 Krakow, Poland

## Abstract

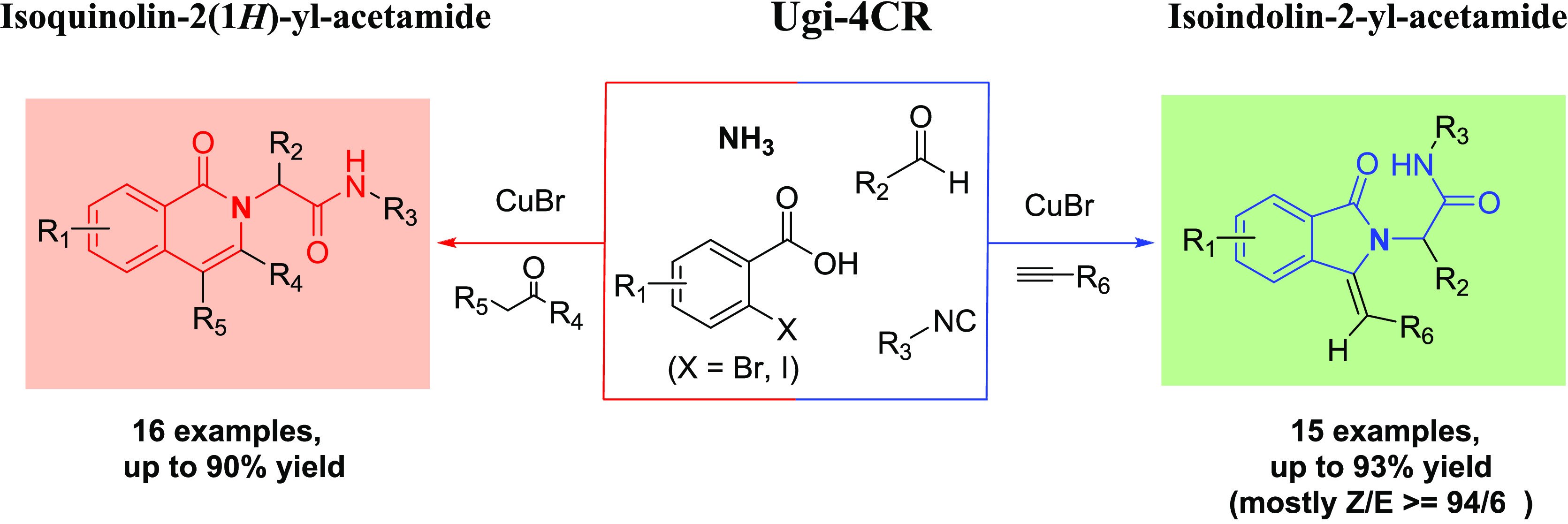

We achieved a divergent synthesis of isoquinolin-2(1*H*)-yl-acetamides (16 examples, up to 90% yields) and regioselective isoindolin-2-yl-acetamides (14 examples, up to 93% yields) in moderate to good yields by reacting various substituted ethanones or terminal alkynes with Ugi-4CR intermediates via an ammonia-Ugi-4CR/Copper(I)-catalyzed annulation sequence reaction. The same intermediate thus gives 2D distant but 3D closely related scaffolds, which can be of high interest in exploiting chemistry space on a receptor. The scopes and limitations of these efficient sequence reactions are described, as well as gram-scale synthesis.

## Introduction

**Figure 1:**
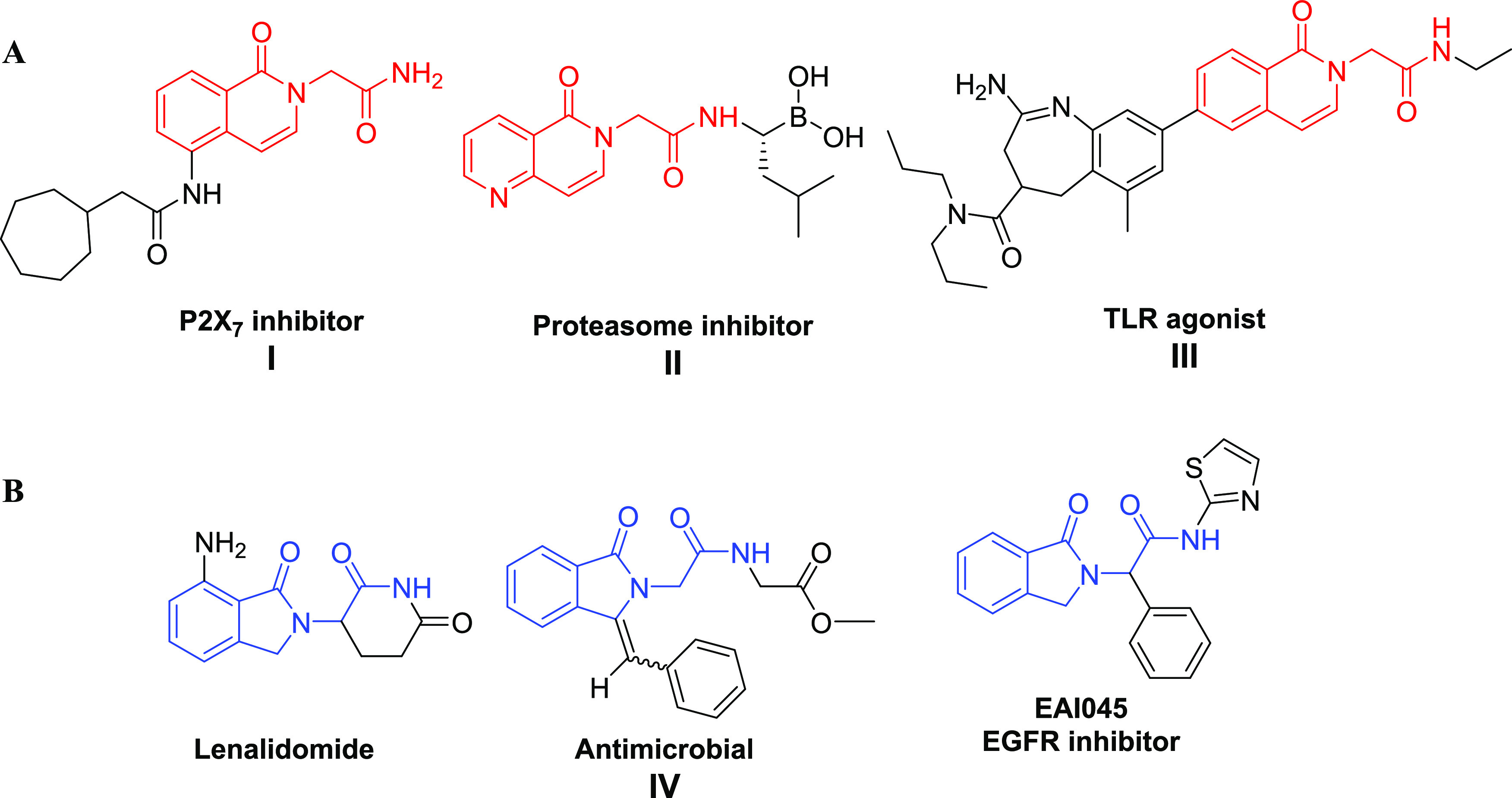
Representative bioactive molecules with isoquinolin-2(1*H*)-yl-acetamide scaffold (A) and isoindolin-2-yl-acetamide skeleton (B).

Multicomponent reactions (MCRs) and post transformations of MCR products have become progressively popular, which has made them some of the most successful methods leading to the rapid generation of small-molecule library of high structural diversity and molecular complexity.^1^ The Ugi reaction, one of the best-known MCRs, with the advantage of atom economy and environmental benefit, could typically afford a linear bis amide backbone.^2^ However, linear bis amides often have issues with stability, solubility, and distribution–metabolism–pharmacokinetic (DMPK) and are not a preferred scaffold in medicinal chemistry.^3^ Therefore, Ugi-4CR and its post-amide-cyclization reactions are widely used in medicinal chemistry research due to the diversity of scope and ability to improve the metabolic stability of final products.^4,5^ Isoquinolin-2(1*H*)-yl-acetamide and isoindolin-2-yl-acetamide have attracted increasing attention due to their possible diverse biological activities. The isoquinolin-2(1*H*)-yl-acetamide scaffold, for example, was found in P2X7 inhibitors **I**, proteasome inhibitors **II** or TLR agonists **III** (Figure 1A).^6−8^ Likewise, the isoindolin-2-yl-acetamide also appears in the FDA-proved anticancer drug Lenalidomide,^9^ as well as the antimicrobial compound **IV** or the EGFR inhibitor EAI045 (Figure 1B).^10−12^

Not surprisingly, new and superior ways to construct the isoquinolin-2(1*H*)-yl-acetamide scaffold or isoindolin-2-yl-acetamide skeleton are in high demand. Yang’s group published a Ugi-4CR/Pd-catalyzed intramolecular arylation reaction to efficiently afford the tricyclic isoquinolin-2(1*H*)-yl-acetamides.^13^

**Scheme 1:**
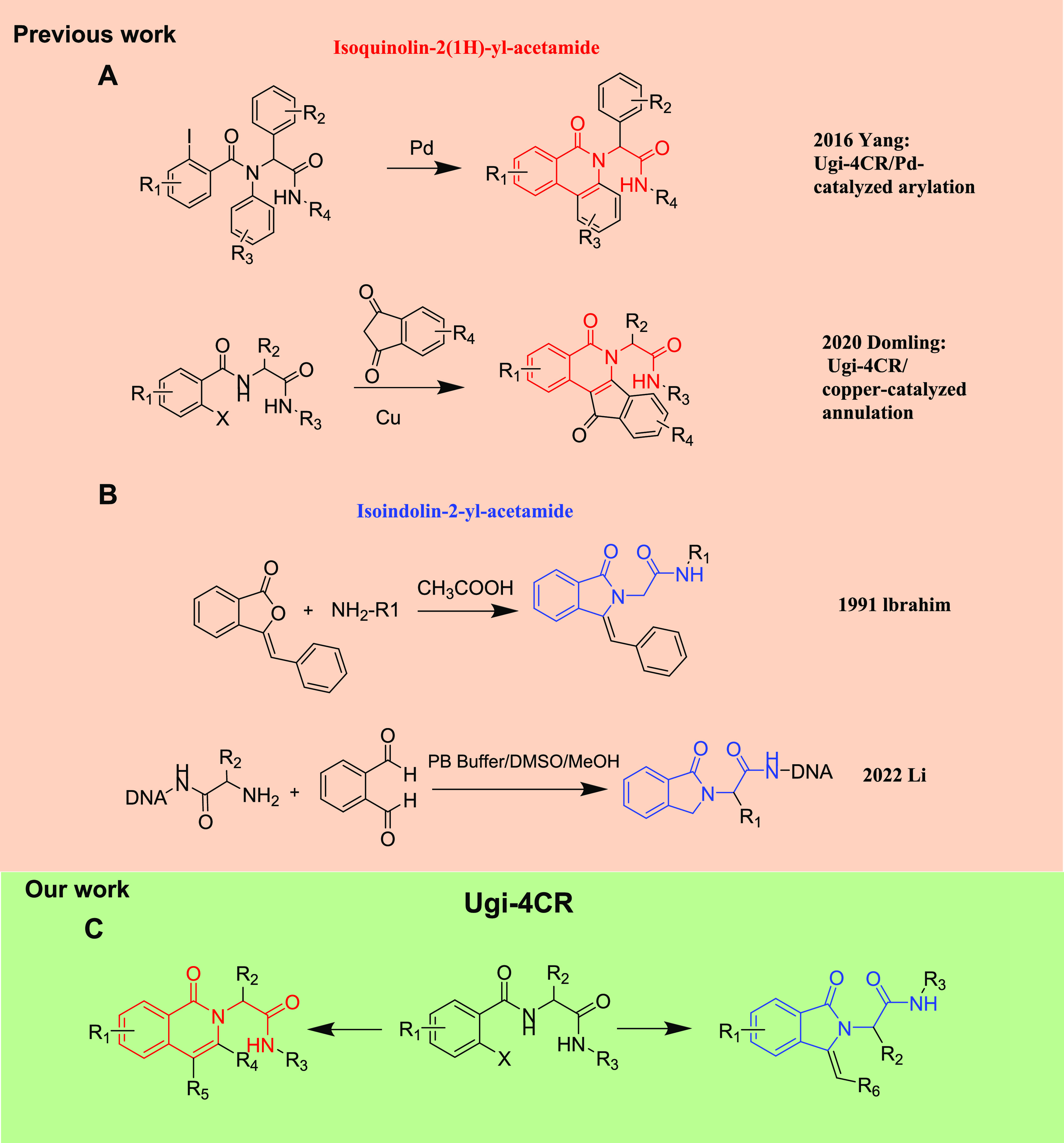
Strategies of Isoquinolin-2(*1*H)-yl-acetamide and Isoindolin-2-yl-acetamide

Recently, our group reported a two-step synthesis of privileged tetracyclic isoquinolin-2(1*H*)-yl-acetamide scaffold via an ammonia-Ugi-4CR/copper-catalyzed annulation sequence (Scheme 1A).^14^ As for the isoindolin-2-yl-acetamides (Scheme 1B), Ibrahim synthesized a series of (1-benzylidene-3-oxoisoindolin-2-yl)-acetamide derivatives from 3-benzalphthalide and the corresponding amino acids.^10^ Most recently, Li’s group developed an efficient protocol to construct a DNA-encoded, isoindolin-2-yl-acetamide-based chemical library with potential lenalidomide-like pharmacological properties.^15^ However, to date, no article has achieved the fast synthesis of both isoquinolin-2(1*H*)-yl-acetamide and isoindolin-2-yl-acetamide derivatives from a common Ugi MCR-based precursor.

Based on previous works on copper-catalyzed cyclization reactions^16^ and our ongoing experience in MCR chemistry and post-MCR modifications,^17^ we hypothesized that two different polycyclic heterocycles could be accessed by a Ugi/Cu-catalyzed annulation sequence reaction between Ugi adducts and different corresponding reagents. Hence, we report herein the synthesis of either isoquinolin-2(1*H*)-yl-acetamides or isoindolin-2-yl-acetamides in moderate to good yields by adopting Cu(I)-catalyzed C–C coupling/annulation reaction of the C(sp^2^)–I/Br bond of Ugi-4CR adducts with substituted ethanones or terminal alkynes, respectively (Scheme 1C).

## Results and Discussion

**Scheme 2:**
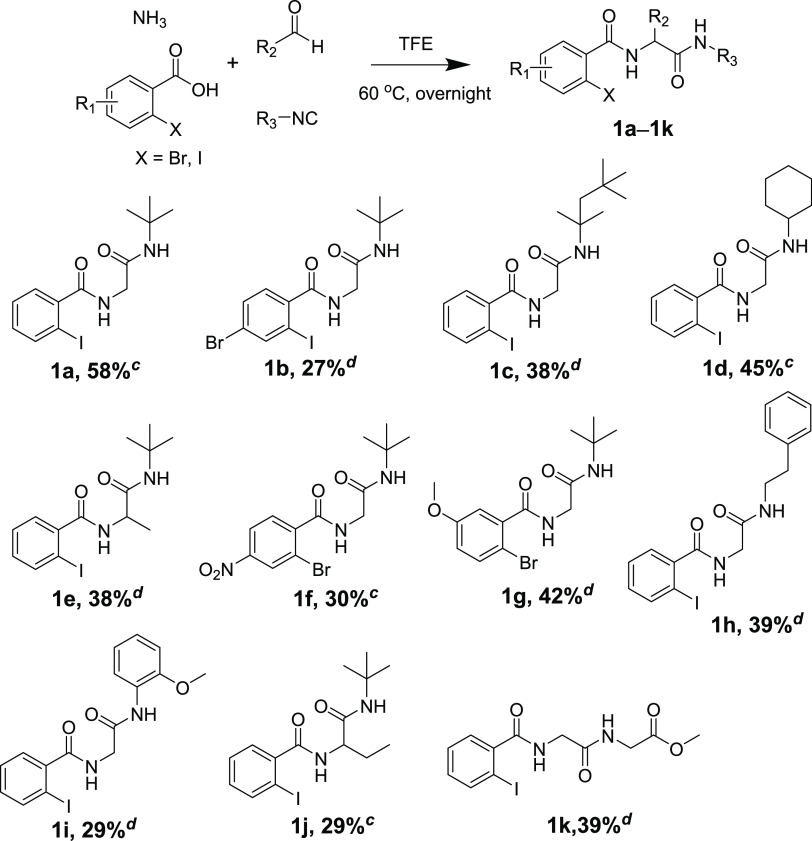
Ugi Adducts in This Work,,, Reaction conditions: carboxylic acid (2 mmol), 25% ammonia solution (2.4 mmol), aldehyde (2 mmol), isocyanide (2 mmol), TFE (2 mL), 60 °C, overnight. Yield refers to the purified products. Published Ugi adducts from our previous articles. New synthesized Ugi adducts in this work.

First, we synthesized 11 Ugi adducts in 27–58% yields via Ugi-ammonia-4CR based on our previous work (Scheme 2).^4,14^

Then, the Ugi adduct **1a** and acetophenone **2a** were selected for the model copper-catalyzed cyclization reaction for the synthesis of isoquinolin-2(1*H*)-yl-acetamide **3a**. The target compound **3a** could be achieved in 62% yield by reacting **1a** with 1.5 equimolar **2a** in DMSO at 80 °C for 16 h under N_2_ in the presence of 10 mol % CuCl and 2 equivalent Cs_2_CO_3_ (Table 1, entry 1). Further optimization was done by variation of the nature of the Cu catalyst, base, solvent, reaction time and temperature (Table 1). CuCl_2_, CuSO_4_, and Cu_2_O all failed to improve yield, affording **3a** in 41, 42, and 39%, respectively (entries 2–4). Comparable to CuCl, CuBr_2_ and CuI could achieve 63 and 61% yield (entries 5 and 6). When replacing CuCl with CuBr, the yield of **3a** slightly increased to 67% (entry 7). K_2_CO_3_ and Na_2_CO_3_ failed to give better yield compared to Cs_2_CO_3_, only trace products were obtained (entries 8 and 9). Further attempts to change the solvent DMSO to MeCN, dioxane, MeOH, toluene, THF and DMF didn’t achieve any yield improvements (entries 10–15). To our delight, increasing the temperature from 80 to 90 or 100 °C provided higher yields of 79% and 78%, respectively (entries 16 and 17). Furthermore, decreasing the reaction time significantly reduced the yield (entries 18–20), and likewise, using microwave irradiation to promote the reaction was not advantageous, resulting in a lower yield (33%, entry 21). Therefore, the optimal reaction condition is: Ugi intermediate **1a** (0.3 mmol), acetophenone **2a** (0.45 mmol), 10 mol % CuBr, and 2.0 equiv of Cs_2_CO_3_ in DMSO (2 mL) at 90 °C for 16 h (entry 16). At the same time, we also screened the reaction condition for the synthesis of isoindolin-2-yl-acetamide **5a** and found the best condition as follows: Ugi intermediate **1a** (0.3 mmol), phenylethyne **4a** (0.45 mmol), 20 mol % CuBr, and 2.0 equiv of K_2_CO_3_ in PEG (2 mL) at 100 °C for 2 h (Table S2, entry 13).

**Table 1:**
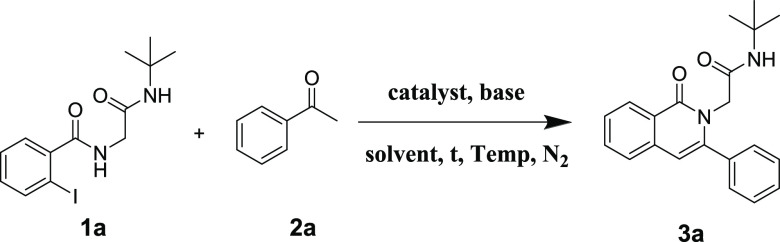
Optimization of Reaction Conditions

entry	catalyst (10 mol %)	base	solvent	time (h)	temp (°C)	yield **3a** (%)
1	CuCl	Cs_2_CO_3_	DMSO(dry)	16	80	62^a^
2	CuCl_2_	Cs_2_CO_3_	DMSO(dry)	16	80	41^b^
3	CuSO4	Cs_2_CO_3_	DMSO(dry)	16	80	42^b^
4	Cu_2_O	Cs_2_CO_3_	DMSO(dry)	16	80	39^b^
5	CuBr_2_	Cs_2_CO_3_	DMSO(dry)	16	80	63^b^
6	CuI	Cs_2_CO_3_	DMSO(dry)	16	80	61^b^
7	CuBr	Cs_2_CO_3_	DMSO(dry)	16	80	67^b^
8	CuBr	K_2_CO_3_	DMSO(dry)	16	80	trace
9	CuBr	Na_2_CO_3_	DMSO(dry)	16	80	trace
10	CuBr	Cs_2_CO_3_	MeCN	16	80	8^b^
11	CuBr	Cs_2_CO_3_	dioxane	16	80	49^b^
12	CuBr	Cs_2_CO_3_	MeOH	16	80	16^b^
13	CuBr	Cs_2_CO_3_	toluene	16	80	trace
14	CuBr	Cs_2_CO_3_	THF	16	80	trace
15	CuBr	Cs_2_CO_3_	DMF	16	80	30^b^
16	CuBr	Cs_2_CO_3_	DMSO(dry)	16	90	79^a^
17	CuBr	Cs_2_CO_3_	DMSO(dry)	16	100	78^a^
18	CuBr	Cs_2_CO_3_	DMSO(dry)	4	80	37^b^
19	CuBr	Cs_2_CO_3_	DMSO(dry)	8	80	43^b^
20	CuBr	Cs_2_CO_3_	DMSO(dry)	12	80	55^b^
21^c^	CuBr	Cs_2_CO_3_	DMSO(dry)	2	80	30^b^

a Reaction conditions: **1a** (0.3 mmol), **2a** (0.45 mmol), catalyst (10 mmol %), base (0.6 mmol), solvent (2 mL), isolated yields.

b Reaction conditions: **1a** (0.1 mmol), **2a** (0.15 mmol), catalyst (10 mmol %), base (0.2 mmol), solvent (1 mL), HPLC yields.

c Microwave.

**Scheme 3:**
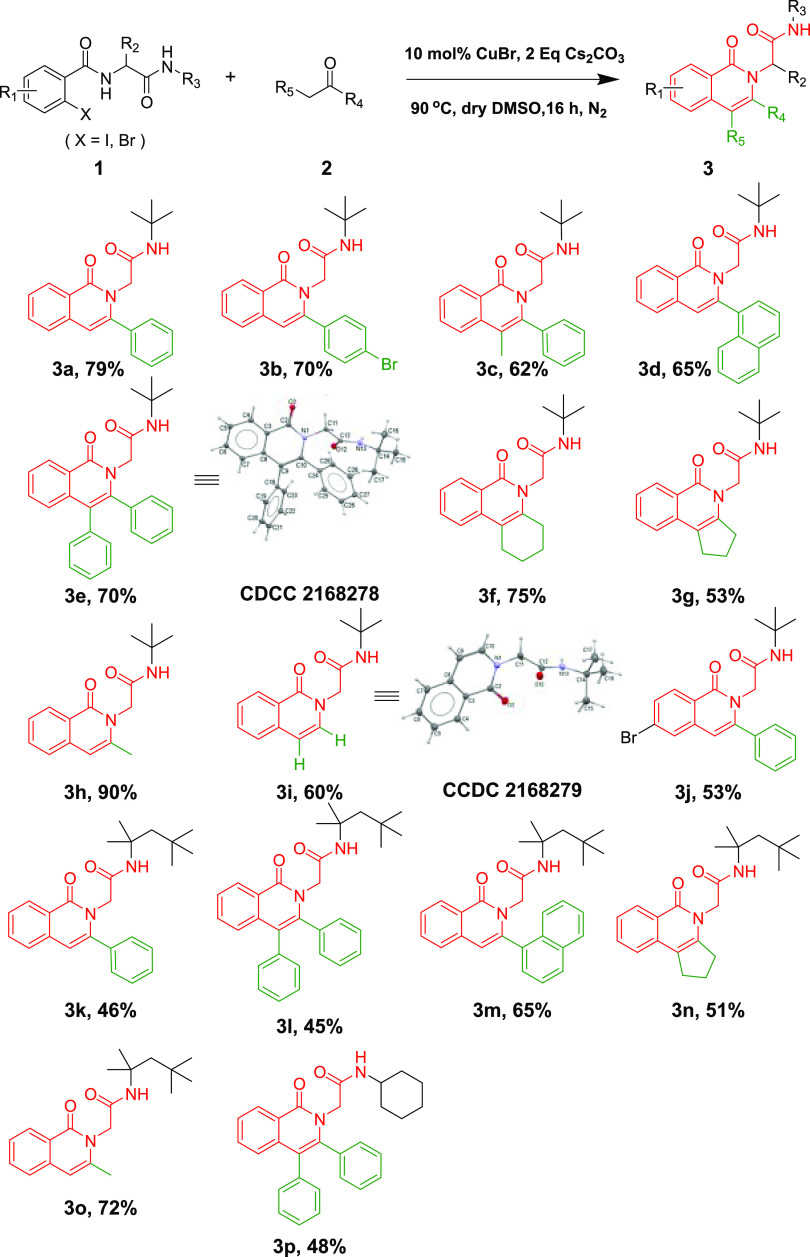
Substrate Scope for the Synthesis of Isoquinolin-2(1*H*)-yl-acetamides, Reaction conditions: **1** (0.3 mmol), **2** (0.45 mmol), Cs_2_CO_3_ (0.6 mmol), CuBr (0.03 mmol), DMSO (2 mL), 90 °C, 16 h. Yield refers to the purified products through a single step.

**Scheme 4:**
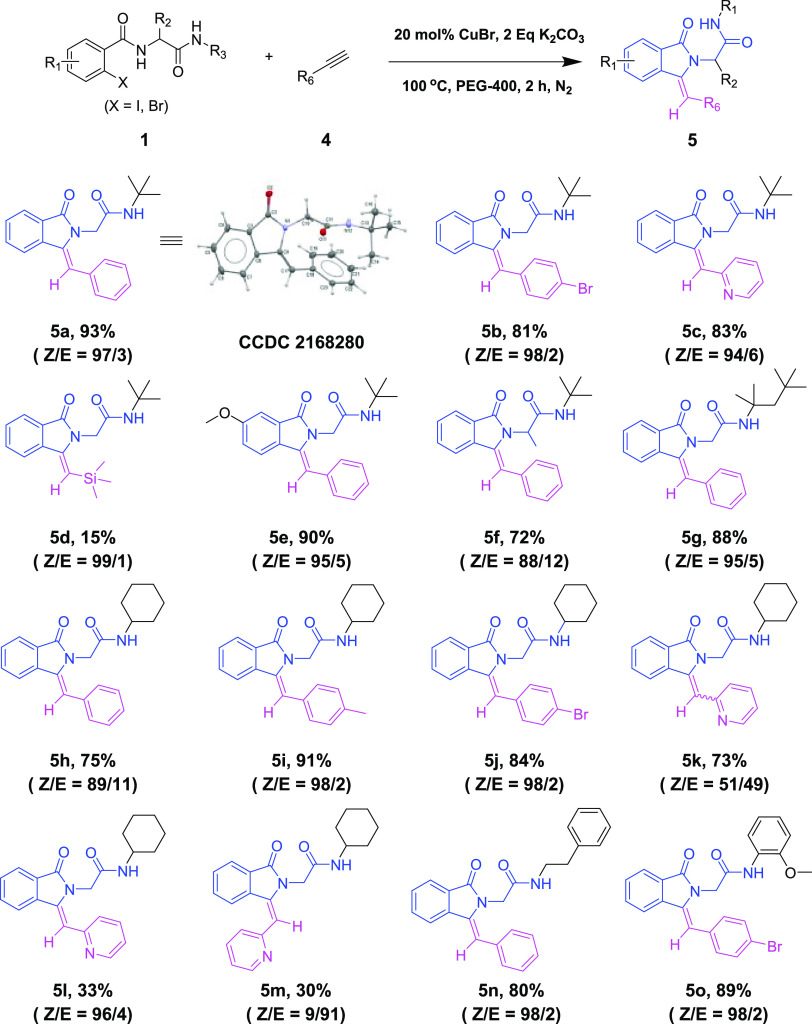
Substrate Scope for the Synthesis of Isoindolin-2-yl-acetamides,, Reaction conditions: **1** (0.3 mmol), **4** (0.45 mmol), K_2_CO_3_ (0.6 mmol), CuBr (0.06 mmol), PEG-400 (2 mL), 100 °C, 2 h. Yield refers to the purified products through a single step. *E*/*Z* was calculated based on ^1^H NMR.

With optimized conditions in hand, we then investigated the substrate scope and limitations toward the corresponding isoquinolin-2(1*H*)-yl-acetamides **3a**–**3p** (Scheme 3) and isoindolin-2-yl-acetamides **5a**–**5o** (Scheme 4). First, we summarized the scope for the synthesis of isoquinolin-2(1*H*)-yl-acetamide derivatives. Overall, paraformaldehyde-based Ugi starting materials were tolerated in the domino reactions to efficiently give the desired products (**3a**–**3p**). Various substituted ethanones performed well with *tert*-butyl isocyanide-derived Ugi adducts in the cyclization reaction to give corresponding products (**3a**–**3j**) in moderate to good yields (53–90%). Aryl-substituted ethanones (acetophenones) proceeded smoothly to afford the isoquinolin-2(1*H*)-yl-acetamide derivatives in good yield (**3a**–**3e**). Among them, acetophenone gave optimal yield (**3a**, 79%), weak electron-withdrawing groups such as 4-Br resulted in slightly lower yield (**3b**, 70%), likewise, double-substituted ethanones like 1-phenyl-2-methyl-ethanone (**3c**, 62%) or 1,2-diphenyl-ethanone (**3e**, 70%) also gave decreased yields. 1-Aceto-naphthone gave 65% yields (**3d**).

When cyclohexanone and cyclopentanone were employed, the desired compounds could be obtained in 75% (**3f**) and 53% (**3g**) yields, comparable to the yields achieved by the above aromatic ethanones. Interestingly, acetone worked very well, providing the target compound (**3h**) in 90% yield. Noteworthy, acetaldehyde was also tolerated in this reaction, yielding 60% of **3i**. The substituted Ugi intermediate 4-Br-2-iodobenzamide worked also well and resulted in the formation of **3j** (53% yield). When Ugi adducts formed by *tert*-octyl-isocyanide were applied to the annulation reaction, the final products (**3k**–**3o**) were obtained in moderate to good yields, whereas the cyclohexyl isocyanide-based Ugi substrate provided compound **3p** in 48% yield. We also observed limitations of the reaction (Table S4**, 3q** and **3r)**. 4-F-acetophenone yielded only trace amounts of product **3q**, possibly due to lower acetyl activity in the structure. In addition, 4-NO_2_-2-iodobenzamide even couldn’t undergo the reaction, probably because it is difficult to achieve oxidative addition with acetophenone. The structures of **3e** and **3i** were unambiguously elucidated by X-ray.

Then, the scope for constructing the isoindolin-2-yl-acetamides was investigated (Scheme 4). Different terminal alkynes could be successfully employed in the reaction and produce the desired target compounds (**5a**–**5d**) with excellent regioselectivity (*Z*/*E* ≥ 94/6), aryl alkynes such as phenylethyne (**5a**), 4-Br-phenylethyne (**5b**), 2-pyridylethyne (**5c**) gave good yields (81–93%), while alkyl alkyne like trimethylsilylacetylene (**5d**) resulted in 15% yield. 5-Methoxy-2-bromobenzamide and ethyl aldehyde originated Ugi adducts were also tolerated, providing compounds **5e** (*Z*/*E* = 95/5, 90%) and **5f** (*Z*/*E* = 88/12, 72%) with good regioselectivity.

Aside from Ugi intermediates based on *tert*-butyl isocyanide, the Ugi adducts generated from other alkyl isonitriles like *tert*-octyl-isocyanide and cyclohexyl isocyanide afforded final products **5g**–**5m** in moderate to excellent yields.

Gratifyingly, Ugi synthons from two aryl nitriles, phenylethyl isocyanide and 2-methoxyphenyl isocyanide could also result in corresponding isoindolin-2-yl-acetamides (**5n**–**5o**) in good yield, thus widely broadening the scope of the reaction. It should be noted that when the Ugi product from cyclohexyl isonitrile reacted with 2-pyridineacetylene, we could obtain a product (**5k**, *Z*/*E* = 51/49) with almost equal amounts of *Z* and *E* forms in 73% yield, which was further purified to give **5l** (*Z*/*E* = 96/4) and **5m** (*Z*/*E* = 9/91) with better regioselectivity. The 2D NMR correlations were done to assign the structure of **5a** which was subsequently unambiguously determined by X-ray (Figure S5).

**Scheme 5:**
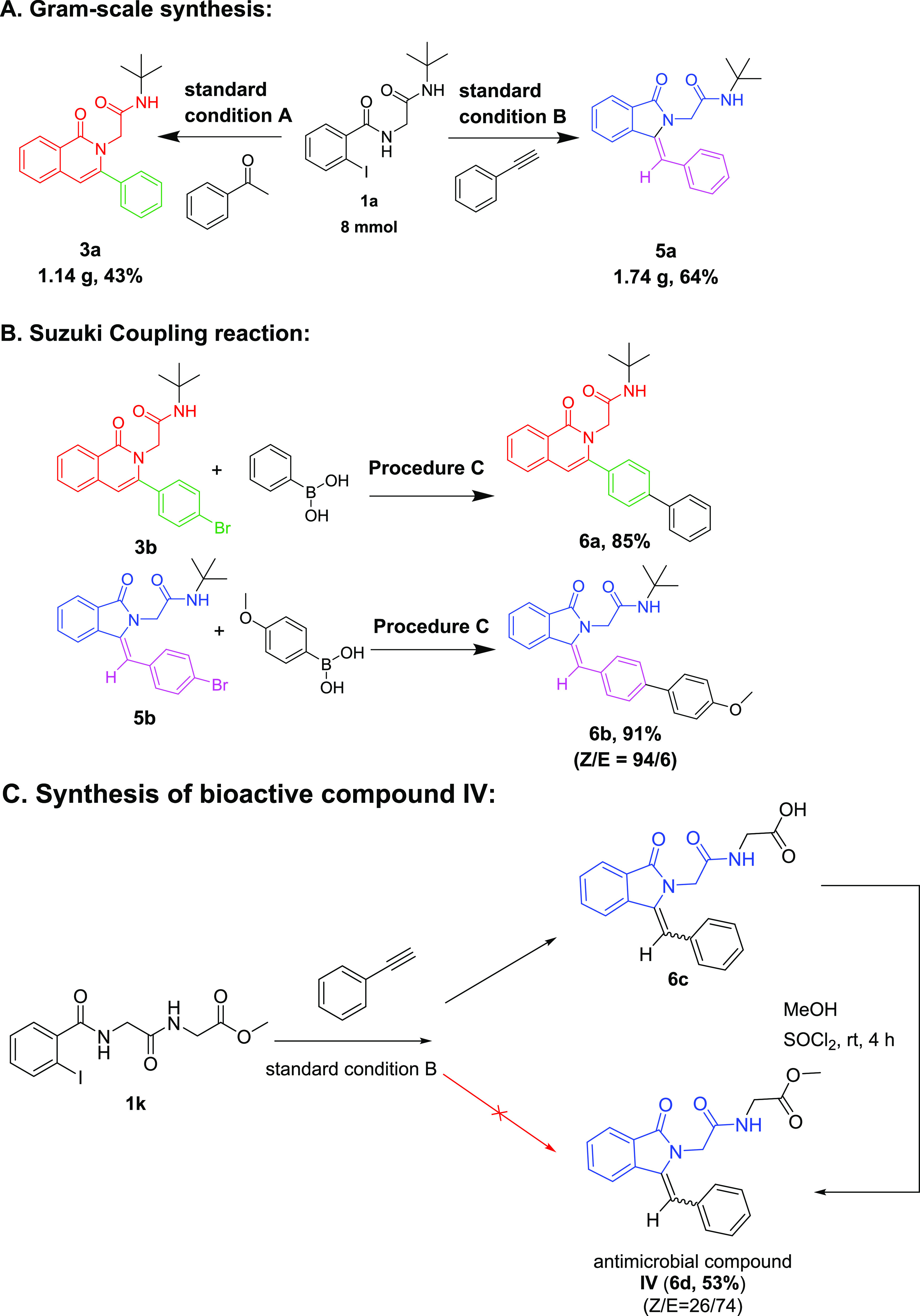
Gram-Scale Reaction, Suzuki Coupling Functionalization, and Bioactive Compound Synthesis

To support the preparative usefulness of our method, gram-scale experiments were carried out for the synthesis of isoquinolin-2(1*H*)-yl-acetamide **3a** and isoindolin-2-yl-acetamide **5a** in moderate yields (Scheme 5A). Two CuBr-promoted cyclization reactions of Ugi-4CR-based 2-iodobenzamide **1a** with acetophenone and phenylethyne were conducted on an 8 mmol scale, producing **3a** (1,14 g) and **5a** (1,74g) in 43% and 64% yield, respectively. To further evaluate the potential of the above-described two scaffolds, we performed the Suzuki coupling late-stage functionalization. **3b** and **5b** were coupled with phenylboronic acid and 4-methoxyphenyl boronic acid separately, giving the corresponding products **6a** and **6b** in good yields via Pd-catalyzed Suzuki reaction (Scheme 5B). We also applied our method to synthesize antibacterial compound **IV** (Scheme 5C); however, instead of obtaining the normal cyclization product **IV** directly, we obtained the carboxylic acid **6c**, which may be due to the similar hydrolysis of the methyl ester occurring in the process as in our previous work.^4^ Then, we reacted **6c** with MeOH and SOCl_2_ to realize product **IV** (**6d**, *Z*/*E* = 26/74) in 53% yield.

**Figure 2:**
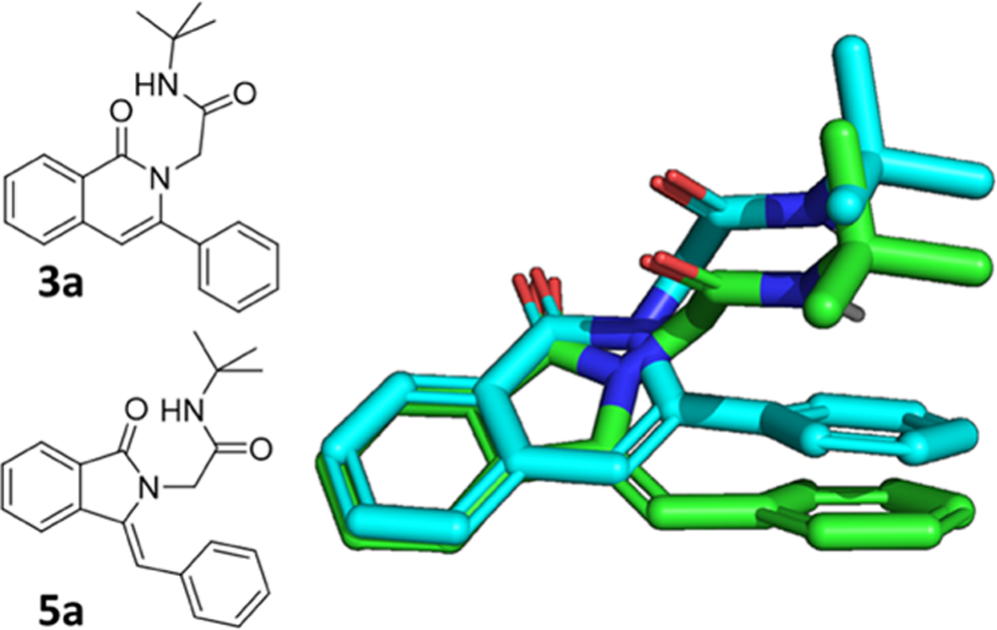
Alignment of energy-minimized isoquinolinone **3a** and isoindolinone **5a** supporting the similar 3D shape of the two scaffolds.

The herein-reported two complementary syntheses of two related scaffolds are based on a common Ugi-4CR intermediate. Switching between two related scaffolds is often applied in medicinal chemistry and sometimes called “scaffold hopping”. It is of high importance during lead optimization as the two related scaffolds might bind similarly into the same receptor site but might have different pharmacokinetic/pharmacodynamic (PKPD) parameters. Clearly, the two scaffolds align well in 3D (Figure 2) and are therefore potential bioisosteres.

## Conclusions

In summary, we developed an efficient Ugi-4CR/copper(I) catalytic system for the synthesis of two different bioactive potential scaffolds: isoquinolin-2(1*H*)-yl-acetamide and isoindolin-2-yl-acetamide, with the advantages of atom economy, good yields and absence of ligands. Additionally, product diversity can be achieved not only through the Ugi starting materials aldehydes, isocyanides, and 2-halogene benzoic acids but also by different substituted ethanones and terminal alkynes. The proposed two different reaction mechanisms were also discussed in the Supporting Information. Having access to two different scaffolds from a common MCR precursor potentially facilitates structure–activity relationship (SAR) enormously, while allowing optimization of “druglike” properties through scaffold hopping.

## Experimental Section

### General Information

Nuclear magnetic resonance spectra were recorded on a Bruker Avance 500 spectrometer. Chemical shifts for ^1^H NMR were reported relative to TMS (δ 0 ppm) or internal solvent peak (CDCl_3_ δ 7.26 ppm, CD_3_OD δ 3.31 ppm or D_2_O δ 4.79 ppm) and coupling constants were in hertz (Hz). The following abbreviations were used for spin multiplicity: s = singlet, d = doublet, t = triplet, dt = double triplet, ddd = doublet of double doublet, m = multiplet, and br = broad. Chemical shifts for ^13^C NMR reported in ppm relative to the solvent peak (CDCl_3_ δ 77.23 ppm, DMSO δ 39.52 ppm, CD_3_OD δ 49.00 ppm). Flash chromatography was performed on a Grace Reveleris X2 using Grace Reveleris Silica columns (12 g) and a gradient of petroleum ether/ethyl acetate (0–100%) or dichloromethane/methanol (0–20%) was applied. Thin layer chromatography was performed on Fluka precoated silica gel plates (0.20 mm thick, particle size 25 μm). All isocyanides were made in-house via the Ugi procedure.^18^ Benzoic acids, ethanones (2), terminal alkynes (4), and other reagents were purchased from Sigma Aldrich, ABCR, Acros, Fluorochem, AK Scientific, Combiblocks, or A2B and were used without further purification. Mass spectra were measured on a Waters Investigator Supercritical Fluid Chromatograph with a 3100 MS Detector (ESI) using a solvent system of methanol and CO_2_ on a Viridis silica gel column (4.6 × 250 mm^2^, 5 μm particle size) and reported as (*m*/*z*). High-resolution mass spectra (HRMS) were recorded using an LTQ-Orbitrap-XL (Thermo Fisher Scientific; ESI pos. mode) at a resolution of 60000@m/z400. Melting points were obtained on a melting point apparatus and were uncorrected. Yields given refer to chromatographically purified compounds unless otherwise stated. Compounds **1a**, **1d**, **1f**, and **1j** were all prepared following our reported literature^4,14^ (Table S3).

### General Experimental Procedure and Characterization

#### General Procedure for Ugi-4CR Products

To a stirred solution of the carboxylic acid (2 mmol, 1.0 equiv) in 2,2,2-trifluoroethanol (2 mL) in a 5 mL vial, 0.3 mL of 25% ammonia solution (2.4 mmol, 1.2 equiv) was added. Aldehyde (2 mmol, 1.0 equiv) and isocyanide (2 mmol, 1.0 equiv) were then introduced into the mixture, the vial was capped, and then the reaction mixture was placed in a heated metal block and stirred at 60 °C overnight. After the completion of the reaction, the solvent was removed in vacuo and the crude products were purified by column chromatography to give the desired products **1b, 1c, 1e**, and **1g–1i** (Table S3).

#### General Procedure A

A sealed tube was charged with Ugi adduct **1** (0.2/0.3 mmol, 1.0 equiv), substituted ethanones (0.3/0.45 mmol, 1.5 equiv), CS_2_CO_3_ (0.4/0.6 mmol, 2.0 equiv), and the CuBr (0.02/0.03 mmol, 0.1 equiv). DMSO (1/2 mL) was added, and the mixture was stirred under N_2_ at 90 °C for 16 h. After the reaction, saturated aqueous NaCl (10 mL) and EtOAc (10 mL) were added successively to the cooled reaction mixture. The organic phase was separated, and the aqueous phase was further extracted with EtOAc (3 × 10 mL). Then, the combined organic layers were dried over anhydrous Na_2_SO_4_ and concentrated. The residues were purified by column chromatography on silica gel to afford the compounds **3a–3p**.

#### General Procedure B

A sealed tube was charged with Ugi adduct **1** (0.2/0.3 mmol, 1.0 equiv), terminal alkynes (0.3/0.45 mmol, 1.5 equiv), K_2_CO_3_(0.4/0.6 mmol, 2.0 equiv), and the CuBr(0.04/0.06 mmol, 0.2 equiv). PEG-400 (1/2 mL) was added, and the mixture was stirred under N_2_ at 100 °C for 2 h. After the reaction, the solvent was removed in vacuo and the residues were purified by column chromatography on silica gel to afford the compounds **5a–5n**.

#### Procedure C

Compound **3b** or **5b** (0.15 mmol, 1.0 equiv) and the corresponding phenylboronic acid (0.225 mmol, 1.5 equiv) were placed in a 10 mL tube, and toluene/ethanol (v/v = 5:1) (3 mL) and sat. NaHCO_3_ (3 mL) were added. The tube was flushed with N_2_ for 10 min, then Pd(dppf)Cl_2_ (0.015 mmol, 0.1 equiv) was added, and the tube was sealed. The mixture was allowed to react at 90 °C in an oil bath for 12 h. Then, the reaction mixture was cooled to room temperature and treated with H_2_O and extracted with EtOAc. The combined organic layers were washed with brine and dried over anhydrous Na_2_SO_4_. After the removal of EtOAc, the residues were purified by silica gel column chromatography to afford Suzuki coupling products **6a** and **6b**.

##### 4-Bromo-*N*-(2-(*tert*-butylamino)-2-oxoethyl)-2-iodobenzamide (**1b**)

It was synthesized according to the procedure of Ugi-4CR reaction on a 2 mmol scale to afford **1b** (236 mg, 27%) as a white solid. mp: 181–182 °C. *R*_f_ = 0.59 (50% EtOAc/petroleum ether). ^1^H NMR (500 MHz, CDCl_3_) δ 8.00 (d, *J* = 1.9 Hz, 1H), 7.48 (dd, *J* = 1.9, 8.2 Hz, 1H), 7.24 (d, *J* = 8.2 Hz, 1H), 7.18 (t, *J* = 5.3 Hz, 1H), 6.46 (s, 1H), 4.02 (d, *J* = 5.0 Hz, 2H), 1.34 (s, 9H). ^13^C{^1^H} NMR (126 MHz, CDCl_3_) δ 168.9, 167.5, 142.2, 140.1, 131.4, 129.5, 124.7, 93.3, 51.8, 44.6, 28.9. HRMS (ESI) *m*/*z*: [M + H]^+^ calcd for C_13_H_17_BrIN_2_O_2_, 438.9518, found, 438.9510.

##### 2-Iodo-*N*-(2-oxo-2-((2,4,4-trimethylpentan-2-yl)amino)ethyl)benzamide (**1c**)

It was synthesized according to the procedure of Ugi-4CR reaction on a 2 mmol scale to afford **1c** (285 mg, 38%) as a white solid. mp: 158–160 °C. *R*_f_ = 0.38 (50% EtOAc/petroleum ether). ^1^H NMR (500 MHz, CDCl_3_) δ 7.87 (dd, *J* = 1.1, 8.0 Hz, 1H), 7.44–7.32 (m, 2H), 7.11 (td, *J* = 1.9, 7.5 Hz, 1H), 6.81 (t, *J* = 5.1 Hz, 1H), 6.13 (s, 1H), 4.05 (d, *J* = 5.2 Hz, 2H), 1.76 (s, 2H), 1.42 (s, 6H), 1.00 (s, 9H). ^13^C{^1^H} NMR (126 MHz, CDCl_3_) δ 169.7, 167.3, 141.3, 140.2, 131.6, 128.5, 128.3, 92.7, 55.9, 51.4, 44.7, 31.8, 31.6, 29.4. HRMS (ESI) *m*/*z*: [M + H]^+^ calcd for C_17_H_26_IN_2_O_2_, 417.1039, found, 417.1021.

##### N-(1-(*tert*-Butylamino)-1-oxopropan-2-yl)-2-iodobenzamide (**1e**)

It was synthesized according to the procedure of Ugi-4CR reaction on a 2 mmol scale to afford **1e** (285 mg, 38%) as a brown oil. *R*_f_ = 0.29 (50% EtOAc/petroleum ether). ^1^H NMR (500 MHz, CDCl_3_) δ 7.93–7.75 (m, 1H), 7.37–7.32 (m, 2H), 7.08 (ddd, *J* = 3.3, 5.8, 7.9 Hz, 1H), 6.69 (d, *J* = 7.7 Hz, 1H), 6.40 (s, 1H), 4.61 (dd, *J* = 6.9, 7.7 Hz, 1H), 1.46 (d, *J* = 6.9 Hz, 3H), 1.35 (s, 9H). ^13^C{^1^H} NMR (126 MHz, CDCl_3_) δ 171.0, 169.1, 141.6, 140.1, 131.4, 128.4, 128.3, 92.6, 51.6, 50.0, 28.9, 18.5. HRMS (ESI) *m*/*z*: [M + H]^+^ calcd for C_14_H_20_IN_2_O_2_, 375.0570, found, 375.0557.

##### 2-Bromo-*N*-(2-(*tert*-butylamino)-2-oxoethyl)-5-methoxybenzamide (**1g**)

It was synthesized according to the procedure of Ugi-4CR reaction on a 2 mmol scale to afford **1g** (287 mg, 42%) as a white solid. mp: 153–155 °C. *R*_f_ = 0.30 (50% EtOAc/petroleum ether). ^1^H NMR (500 MHz, CDCl_3_) δ 7.45 (dd, *J* = 0.9, 8.8 Hz, 1H), 7.23 (t, *J* = 5.1 Hz, 1H), 7.05 (dd, *J* = 1.0, 3.1 Hz, 1H), 6.83 (ddd, *J* = 0.9, 3.2, 8.8 Hz, 1H), 6.55 (s, 1H), 4.08 (d, *J* = 5.2 Hz, 2H), 3.79 (d, *J* = 0.9 Hz, 3H), 1.37 (d, *J* = 1.1 Hz, 9H). ^13^C{^1^H} NMR (126 MHz, CDCl_3_) δ 167.8, 167.6, 158.9, 137.7, 134.3, 118.0, 114.6, 109.6, 55.7, 51.7, 44.5, 28.8. HRMS (ESI) *m*/*z*: [M + H]^+^ calcd for C_14_H_20_BrN_2_O_3_, 343.0657, found, 343.0645.

##### 2-Iodo-*N*-(2-oxo-2-(phenethylamino)ethyl)benzamide (**1h**)

It was synthesized according to the procedure of Ugi-4CR reaction on a 2 mmol scale to afford **1h** (318 mg, 39%) as a white solid. mp: 167–169 °C. *R*_f_ = 0.44 (50% EtOAc/petroleum ether). ^1^H NMR (500 MHz, CDCl_3_) δ 7.87–7.83 (m, 1H), 7.38–7.34 (m, 2H), 7.30–7.26 (m, 2H), 7.23–7.16 (m, 3H), 7.11 (ddd, *J* = 3.1, 6.1, 7.9 Hz, 1H), 6.80 (t, *J* = 5.3 Hz, 1H), 6.63 (t, *J* = 5.9 Hz, 1H), 4.10 (d, *J* = 5.2 Hz, 2H), 3.55 (td, *J* = 5.8, 7.2 Hz, 2H), 2.83 (t, *J* = 7.2 Hz, 2H). ^13^C{^1^H} NMR (126 MHz, CDCl_3_) δ 169.7, 168.4, 141.2, 140.1, 138.7, 131.6, 128.9, 128.8, 128.5, 128.3, 126.7, 92.6, 43.8, 40.9, 35.7. HRMS (ESI) *m*/*z*: [M + H]^+^ calcd for C_17_H_18_IN_2_O_2_, 409.0413, found, 409.0400.

##### 2-Iodo-*N*-(2-((2-methoxyphenyl)amino)-2-oxoethyl)benzamide (**1i**)

It was synthesized according to the procedure of Ugi-4CR reaction on a 2 mmol scale to afford **1i** (238 mg, 29%) as a white solid. 181–183 °C. *R*_f_ = 0.54 (50% EtOAc/petroleum ether). ^1^H NMR (500 MHz, CDCl_3_) δ 8.30 (dd, *J* = 1.7, 8.0 Hz, 1H), 8.26 (s, 1H), 7.89 (dd, *J* = 1.1, 8.0 Hz, 1H), 7.47 (dd, *J* = 1.9, 7.6 Hz, 1H), 7.40 (td, *J* = 1.3, 7.6 Hz, 1H), 7.13 (td, *J* = 1.8, 7.6 Hz, 1H), 7.08 (td, *J* = 1.7, 7.9 Hz, 1H), 6.96 (td, *J* = 1.4, 7.9 Hz, 1H), 6.92–6.87 (m, 1H), 6.73 (s, 1H), 4.33 (d, *J* = 5.2 Hz, 2H), 3.89 (s, 3H). ^13^C{^1^H} NMR (126 MHz, CDCl_3_) δ 169.6, 166.2, 148.0, 141.3, 140.1, 131.5, 128.5, 128.2, 126.9, 124.4, 121.1, 120.0, 110.1, 92.5, 55.8, 44.5. HRMS (ESI) *m*/*z*: [M + H]^+^ calcd for C_16_H_16_IN_2_O_3_, 411.0206, found, 411.0190.

##### Methyl (2-iodobenzoyl)glycylglycinate (**1j**)

It was synthesized according to the procedure of Ugi-4CR reaction on a 2 mmol scale to afford **1j** (293 mg, 39%) as a white solid. 100–102 °C. *R*_f_ = 0.24 (3% MeOH/DCM). ^1^H NMR (500 MHz, CDCl_3_) δ 7.83 (d, *J* = 8.0 Hz, 1H), 7.39 (dd, *J* = 7.6, 1.8 Hz, 1H), 7.35 (t, *J* = 7.5 Hz, 1H), 7.30 (d, *J* = 6.4 Hz, 1H), 7.15–7.02 (m, 2H), 4.22 (d, *J* = 5.3 Hz, 2H), 4.04 (d, *J* = 5.5 Hz, 2H), 3.71 (s, 3H). ^13^C{^1^H} NMR (126 MHz, CDCl_3_) δ 170.1, 169.9, 169.1, 141.2, 140.0, 131.5, 128.5, 128.3, 92.6, 52.5, 43.6. HRMS (ESI) *m*/*z*: [M + H]^+^ calcd for C_12_H_14_IN_2_O_4_I, 376.9998, found, 376.9988.

##### N-(*tert*-Butyl)-2-(1-oxo-3-phenylisoquinolin-2(1H)-yl)acetamide (**3a**)

It was synthesized according to procedure A on a 0.3 mmol scale to yield final compound **3a** (79 mg, 79%) as a yellow solid. mp: 203–205 °C. *R*_f_ = 0.48 (30% EtOAc/petroleum ether). ^1^H NMR (500 MHz, CDCl_3_) δ 8.42 (d, *J* = 8.0 Hz, 1H), 7.68–7.62 (m, 1H), 7.51–7.43 (m, 7H), 6.48 (s, 1H), 5.92 (s, 1H), 4.43 (s, 2H), 1.31 (s, 9H). ^13^C{^1^H} NMR (126 MHz, CDCl_3_) δ 167.0, 163.5, 143.9, 136.7, 135.7, 132.8, 129.4, 129.3, 128.7, 128.2, 126.9, 126.1, 124.8, 108.2, 51.5, 50.4, 28.8. HRMS (ESI) *m*/*z*: [M + H]^+^ calcd for C_21_H_23_O_2_N_2_, 335.1754, found, 335.1746.

##### 2-(3-(4-Bromophenyl)-1-oxoisoquinolin-2(1H)-yl)-*N*-(*tert*-butyl)acetamide (**3b**)

It was synthesized according to procedure A on a 0.2 mmol scale to yield final compound **3b** (58 mg, 70%) as a white solid. mp: 214-216 °C. *R*_f_ = 0.31 (25% EtOAc/petroleum ether). ^1^H NMR (500 MHz, CDCl_3_) δ 8.43 (dd, *J* = 8.1, 1.3 Hz, 1H), 7.67 (td, *J* = 7.4, 1.5 Hz, 1H), 7.59 (d, *J* = 8.5 Hz, 2H), 7.54–7.47 (m, 2H), 7.38 (d, *J* = 8.4 Hz, 2H), 6.46 (s, 1H), 5.87 (s, 1H), 4.38 (s, 2H), 1.33 (s, 9H). ^13^C{^1^H} NMR (126 MHz, CDCl_3_) δ 166.9, 163.5, 142.8, 136.6, 134.6, 133.0, 132.0, 131.2, 128.3, 127.2, 126.2, 125.0, 123.9, 108.4, 51.7, 50.6, 28.8. HRMS (ESI) *m*/*z*: [M + H]^+^ calcd for C_21_H_22_O_2_N_2_Br, 413.0859, found, 413.0858.

##### N-(*tert*-Butyl)-2-(4-methyl-1-oxo-3-phenylisoquinolin-2(1H)-yl)acetamide (**3c**)

It was synthesized according to procedure A on a 0.3 mmol scale to yield the final compound **3c** (65 mg, 62%) as a white solid. mp: 178-180 °C. *R*_f_ = 0.29 (33% EtOAc/petroleum ether). ^1^H NMR (500 MHz, CDCl_3_) δ 8.52 (dt, *J* = 8.0, 1.3 Hz, 1H), 7.77–7.67 (m, 2H), 7.54 (ddd, *J* = 8.2, 6.4, 1.9 Hz, 1H), 7.47 (dd, *J* = 5.0, 1.9 Hz, 3H), 7.32–7.28 (m, 2H), 5.65 (s, 1H), 4.34 (s, 2H), 2.02 (s, 3H), 1.28 (s, 9H). ^13^C{^1^H} NMR (126 MHz, CDCl_3_) δ 166.9, 162.8, 140.0, 137.6, 135.1, 132.8, 130.0, 129.1, 129.0, 128.5, 126.8, 125.2, 123.5, 111.3, 51.5, 50.5, 28.9, 28.8, 15.1. HRMS (ESI) *m*/*z*: [M + H]^+^ calcd for C_22_H_25_O_2_N_2_, 349.1911, found, 349.1910. [M + Na]^+^ calcd for C_22_H_24_O_2_N_2_Na, 371.1731, found, 371.1729.

##### N-(*tert*-Butyl)-2-(3-(naphthalen-1-yl)-1-oxoisoquinolin-2(1H)-yl)acetamide (**3d**)

It was synthesized according to procedure A on a 0.3 mmol scale to yield final compound **3d** (75 mg, 65%) as a white solid. mp: 217–219 °C. *R*_f_ = 0.43 (30% EtOAc/petroleum ether). ^1^H NMR (500 MHz, CDCl_3_) δ 8.51 (d, *J* = 8.0 Hz, 1H), 7.97 (d, *J* = 8.1 Hz, 1H), 7.93 (d, *J* = 7.1 Hz, 1H), 7.69 (t, *J* = 7.5 Hz, 1H), 7.63–7.58 (m, 2H), 7.54 (dd, *J* = 13.4, 8.1 Hz, 4H), 7.46 (t, *J* = 6.9 Hz, 1H), 6.59 (s, 1H), 5.46 (s, 1H), 1.61 (s, 2H), 1.21 (s, 9H). ^13^C{^1^H} NMR (126 MHz, CDCl_3_) δ 166.5, 163.5, 141.5, 136.8, 133.4, 132.9, 132.4, 132.0, 129.9, 128.8, 128.7, 128.4, 127.4, 127.1, 126.6, 126.2, 125.5, 125.1, 109.1, 51.5, 49.6, 28.7. HRMS (ESI) *m*/*z*: [M + H]^+^ calcd for C_25_H_25_O_2_N_2_, 385.1911, found, 385.1913.

##### N-(*tert*-Butyl)-2-(1-oxo-3,4-diphenylisoquinolin-2(1H)-yl)acetamide (**3e**)

It was synthesized according to procedure A on a 0.3 mmol scale to yield final compound **3e** (86 mg, 70%) as a white solid. mp: 209–211 °C. *R*_f_ = 0.19(20% EtOAc/petroleum ether). ^1^H NMR (500 MHz, CDCl_3_) δ 8.53 (dd, *J* = 8.0, 1.7 Hz, 1H), 7.52 (dddd, *J* = 27.0, 8.2, 7.1, 1.5 Hz, 2H), 7.22–7.12 (m, 9H), 7.11–7.05 (m, 2H), 5.73 (s, 1H), 4.43 (s, 2H), 1.30 (s, 9H). ^13^C{^1^H} NMR (126 MHz, CDCl3) δ 166.8, 162.9, 141.2, 137.6, 136.5, 134.4, 132.6, 131.6, 130.6, 128.6, 128.2, 128.2, 128.0, 127.0, 126.8, 125.7, 124.9, 119.6, 51.6, 50.4, 28.8. HRMS (ESI) *m*/*z*: [M + H]^+^ calcd for C_27_H_27_O_2_N_2_, 411.2054,found, 411.2059.

##### N-(*tert*-Butyl)-2-(6-oxo-2,3,4,6-tetrahydrophenanthridin-5(1H)-yl)acetamide (**3f**)

It was synthesized according to procedure A on a 0.3 mmol scale to yield final compound **3f** (70 mg, 75%) as a white solid. m.p.: 204–206 °C. *R*_f_ = 0.52(20% EtOAc/petroleum ether). ^1^H NMR (500 MHz, CDCl_3_) δ 8.43 (dd, *J* = 8.1, 1.7 Hz, 1H), 7.67 (dd, *J* = 6.8, 1.4 Hz, 1H), 7.62 (dd, *J* = 8.3, 1.3 Hz, 1H), 7.45 (s, 1H), 6.57 (s, 1H), 4.67 (s, 2H), 2.82–2.74 (m, 4H), 1.86 (ddd, *J* = 13.2, 4.7, 3.0 Hz, 4H), 1.30 (s, 9H).; ^13^C{^1^H} NMR (126 MHz, CDCl3) δ 167.8, 163.2, 137.3, 137.2, 132.7, 128.3, 126.2, 124.2, 121.8, 111.8, 51.5, 48.8, 28.8, 27.6, 24.4, 22.8, 22.0. HRMS (ESI) *m*/*z*: [M + H]^+^ calcd for C_19_H_25_O_2_N_2_, 313.1911, found, 313.1911.

##### N-(*tert*-Butyl)-2-(5-oxo-1,2,3,5-tetrahydro-4H-cyclopenta[*c*]isoquinolin-4-yl)acetamide (**3g**)

It was synthesized according to procedure A on a 0.3 mmol scale to yield final compound **3g** (47 mg, 53%) as a white solid. mp: 227–229 °C. *R*_f_ = 0.36 (30% EtOAc/petroleum ether). ^1^H NMR (500 MHz, CDCl_3_) δ 8.39 (d, *J* = 8.1 Hz, 1H), 7.65 (t, *J* = 7.5 Hz, 1H), 7.49–7.36 (m, 2H), 6.53 (s, 1H), 4.57 (s, 2H), 3.14–3.03 (m, 2H), 2.97 (t, *J* = 7.4 Hz, 2H), 2.22 (q, *J* = 7.5 Hz, 2H), 1.30 (s, 9H). ^13^C{^1^H} NMR (126 MHz, CDCl_3_) δ 167.2, 163.7, 142.6, 135.5, 132.7, 128.8, 125.8, 124.3, 123.1, 116.0, 51.5, 50.8, 32.6, 29.0, 28.8, 21.5. HRMS (ESI) *m*/*z*: [M + H]^+^ calcd for C_18_H_23_O_2_N_2_, 299.1754, found, 299.1754.

##### N-(*tert*-Butyl)-2-(3-methyl-1-oxoisoquinolin-2(1H)-yl)acetamide (**3h**)

It was synthesized according to procedure A on a 0.3 mmol scale to yield final compound **3h** (73 mg, 90%) as a white solid. mp: 176-178 °C. *R*_f_ = 0.3 (25% EtOAc/petroleum ether). ^1^H NMR (500 MHz, CDCl_3_) δ 8.43–8.19 (m, 1H), 7.61 (td, *J* = 7.6, 1.6 Hz, 1H), 7.46–7.39 (m, 2H), 6.41 (s, 1H), 6.36 (d, *J* = 5.4 Hz, 1H), 4.67 (s, 2H), 2.48 (d, *J* = 0.9 Hz, 3H), 1.31 (s, 9H). ^13^C{^1^H} NMR (126 MHz, CDCl_3_) δ 167.4, 163.8, 139.6, 137.1, 132.8, 128.1, 126.3, 125.4, 124.2, 106.7, 51.6, 49.4, 28.8, 20.9. HRMS (ESI) *m*/*z*: [M + H]^+^ calcd for C_16_H_21_O_2_N_2_, 273.1598; found, 273.1598. [M + Na]^+^ calcd for C_16_H_20_O_2_N_2_Na, 295.1417, found, 295.1414.

##### N-(*tert*-Butyl)-2-(1-oxoisoquinolin-2(1H)-yl)acetamide (**3i**)

It was synthesized according to procedure A on a 0.2 mmol scale to yield final compound **3i** (31 mg, 60%) as a white solid. mp: 202-204 °C. *R*_f_ = 0.12 (25% EtOAc/petroleum ether). ^1^H NMR (500 MHz, CDCl_3_) δ 8.42 (dd, *J* = 8.1, 1.3 Hz, 1H), 7.66 (ddd, *J* = 8.4, 7.1, 1.4 Hz, 1H), 7.54 (dd, *J* = 8.0, 1.6 Hz, 1H), 7.50 (ddd, *J* = 8.2, 7.0, 1.3 Hz, 1H), 7.17 (d, *J* = 7.4 Hz, 1H), 6.56 (d, *J* = 7.3 Hz, 1H), 6.44 (s, 1H), 4.52 (s, 2H), 1.31 (s, 9H). ^13^C{^1^H} NMR (126 MHz, CDCl_3_) δ 166.9, 162.7, 137.4, 132.7, 132.1, 128.0, 127.2, 126.3, 125.9, 107.0, 54.2, 51.7, 28.8. HRMS (ESI) *m*/*z*: [M + H]^+^ calcd for C_15_H_19_O_2_N_2_, 259.1441, found, 259.1441.

##### 2-(6-Bromo-1-oxo-3-phenylisoquinolin-2(1H)-yl)-*N*-(*tert*-butyl)acetamide (**3j**)

It was synthesized according to procedure A on a 0.2 mmol scale to yield final compound **3j** (44 mg, 53%) as a white solid. mp: 215-217 °C. *R*_f_ = 0.36 (20% EtOAc/petroleum ether). ^1^H NMR (500 MHz, CDCl_3_) 8.28 (d, *J* = 8.6 Hz, 1H), 7.58 (s, 1H), 7.58 (ddd, *J* = 8.6, 2.0, 0.9 Hz, 1H), 7.48–7.43 (m, 5H), 6.38 (s, 1H), 5.65 (s, 1H), 4.39 (s, 2H), 1.32 (s, 9H). ^13^C{^1^H} NMR (126 MHz, CDCl_3_) δ 166.6, 163.0, 145.3, 138.1, 135.3, 130.14, 130.09, 129.5, 129.3, 128.8, 128.5, 127.9, 123.5, 106.9, 51.7, 50.3, 28.8. HRMS (ESI) *m*/*z*: [M + H]^+^ calcd for C_21_H_22_O_2_N_2_Br, 413.0859, found, 413.0858.

##### 2-(1-Oxo-3-phenylisoquinolin-2(1H)-yl)-*N*-(2,4,4-trimethylpentan-2-yl)acetamide (**3k**)

It was synthesized according to procedure A on a 0.3 mmol scale to yield final compound **3k** (54 mg, 46%) as a white solid. mp: 203–206 °C. *R*_f_ = 0.26 (20% EtOAc/petroleum ether). ^1^H NMR (500 MHz, CDCl_3_) δ 8.51–8.32 (m, 1H), 7.64 (ddd, *J* = 8.2, 7.1, 1.4 Hz, 1H), 7.53–7.40 (m, 7H), 6.48 (s, 1H), 6.04 (s, 1H), 4.44 (s, 2H), 1.67 (s, 2H), 1.36 (s, 6H), 0.89 (s, 9H).; ^13^C{^1^H} NMR (126 MHz, CDCl_3_) δ 166.6, 163.4, 143.9, 136.6, 135.6, 132.8, 129.5, 129.3, 128.6, 128.1, 126.9, 126.1, 124.8, 108.3, 55.5, 51.8, 50.5, 31.6, 31.5, 29.1. HRMS (ESI) *m*/*z*: [M + H]^+^ calcd for C_25_H_31_O_2_N_2_, 391.2380, found, 391.2377.

##### 2-(1-Oxo-3,4-diphenylisoquinolin-2(1H)-yl)-*N*-(2,4,4-trimethylpentan-2-yl)acetamide (**3l**)

It was synthesized according to procedure A on a 0.3 mmol scale to yield final compound **3l** (63 mg, 45%) as a white solid. mp: 184-186 °C. *R*_f_ = 0.68 (30% EtOAc/petroleum ether). ^1^H NMR (500 MHz, CDCl_3_) δ 8.53 (dd, *J* = 8.0, 1.8 Hz, 1H), 7.52 (dddd, *J* = 25.5, 8.4, 7.1, 1.5 Hz, 2H), 7.21–7.12 (m, 9H), 7.09–7.06 (m, 2H), 5.80 (s, 1H), 4.42 (s, 2H), 1.66 (s, 2H), 1.36 (s, 6H), 0.90 (s, 9H). ^13^C{^1^H} NMR (126 MHz, CDCl_3_) δ 166.4, 162.8, 141.2, 137.6, 136.5, 134.4, 132.6, 131.6, 130.6, 128.6, 128.2, 128.0, 127.0, 126.9, 125.6, 124.9, 120.0, 55.5, 52.0, 50.6, 31.7, 31.5, 29.1. HRMS (ESI) *m*/*z*: [M + H]^+^ calcd for C_31_H_35_O_2_N_2_, 467.2693, found, 467.2689.

##### 2-(3-(Naphthalen-1-yl)-1-oxoisoquinolin-2(1H)-yl)-*N*-(2,4,4-trimethylpentan-2-yl)acetamide (**3m**)

It was synthesized according to procedure A on a 0.3 mmol scale to yield final compound **3m** (86 mg, 65%) as a white solid. mp: 218–220 °C. *R*_f_ = 0.63 (30% EtOAc/petroleum ether). ^1^H NMR (500 MHz, CDCl_3_) δ 8.55–8.39 (m, 1H), 7.95 (dd, *J* = 20.3, 7.7 Hz, 2H), 7.69 (d, *J* = 1.6 Hz, 1H), 7.62–7.57 (m, 2H), 7.57–7.49 (m, 4H), 7.46 (s, 1H), 6.60 (s, 1H), 5.62 (s, 1H), 4.80 (d, *J* = 15.3 Hz, 1H), 3.76 (d, *J* = 15.6 Hz, 1H), 1.62 (s, 2H), 1.27 (d, *J* = 3.0 Hz, 6H), 0.83 (s, 9H). ^13^C{^1^H} NMR (126 MHz, CDCl_3_) δ 166.1, 163.4, 141.6, 136.8, 133.5, 132.9, 132.4, 132.0, 130.0, 128.8, 128.7, 128.3, 127.4, 127.1, 126.6, 126.2, 125.5, 125.2, 125.0, 109.1, 55.4, 51.9, 49.8, 31.6, 31.4, 29.0 (d, *J* = 6.0 Hz). HRMS (ESI) *m*/*z*: [M + H]^+^ calcd for C_29_H_33_O_2_N_2_, 441.2537, found, 441.2534.

##### 2-(5-Oxo-1,2,3,5-tetrahydro-4H-cyclopenta[*c*]isoquinolin-4-yl)-*N*-(2,4,4-trimethylpentan-2-yl)acetamide (**3n**)

It was synthesized according to procedure A on a 0.3 mmol scale to yield final compound **3n** (54 mg, 51%) as a white solid. mp: 164–166 °C. *R*_f_ = 0.22 (20% EtOAc/petroleum ether). ^1^H NMR (500 MHz, CDCl_3_) δ 8.40 (dd, *J* = 8.1, 1.7 Hz, 1H), 7.72–7.60 (m, 1H), 7.50–7.39 (m, 2H), 6.57 (s, 1H), 4.58 (s, 2H), 3.11 (t, *J* = 7.9 Hz, 1H), 2.99 (t, *J* = 7.4 Hz, 1H), 2.22 (p, *J* = 7.4 Hz, 2H), 1.66 (s, 2H), 1.36 (s, 6H), 0.85 (s, 9H). ^13^C{^1^H} NMR (126 MHz, CDCl_3_) δ 166.9, 163.8, 142.5, 135.5, 132.8, 128.8, 125.9, 124.4, 123.2, 116.2, 77.4, 77.2, 76.9, 55.4, 51.7, 51.3, 32.6, 31.6, 31.3, 29.2, 29.1, 21.7. HRMS (ESI) *m*/*z*: [M + H]^+^ calcd for C_22_H_31_O_2_N_2_, 355.2380, found, 355.2377.

##### 2-(3-Methyl-1-oxoisoquinolin-2(1H)-yl)-*N*-(2,4,4-trimethylpentan-2-yl)acetamide (**3o**)

It was synthesized according to procedure A on a 0.2 mmol scale to yield final compound **3o** (47 mg, 72%) as a yellow oil. mp: 145–147 °C. *R*_f_ = 0.68 (5% Acetone/DCM). ^1^H NMR (500 MHz, CDCl_3_) δ 8.35 (dd, *J* = 8.0, 1.3 Hz, 1H), 7.62 (ddd, *J* = 8.2, 7.1, 1.3 Hz, 1H), 7.45–7.40 (m, 2H), 6.56 (s, 1H), 6.41 (s, 1H), 4.67 (s, 2H), 2.50 (d, *J* = 0.9 Hz, 3H), 1.66 (s, 2H), 1.36 (s, 6H), 0.84 (s, 9H). ^13^C{^1^H} NMR (126 MHz, CDCl_3_) δ 167.0, 163.8, 139.5, 136.9, 132.8, 128.1, 126.3, 125.4, 124.1, 106.8, 55.4, 51.7, 49.8, 31.6, 31.3, 29.2, 21.0. HRMS (ESI) *m*/*z*: [M + H]^+^ calcd for C_20_H_29_O_2_N_2_, 329.2224, found, 329.2220.

##### N-Cyclohexyl-2-(1-oxo-3,4-diphenylisoquinolin-2(1H)-yl)acetamide (**3p**)

It was synthesized according to procedure A on a 0.3 mmol scale to yield final compound **3p** (63 mg, 48%) as a white solid. mp: 149–151 °C. *R*_f_ = 0.28 (30% EtOAc/petroleum ether). ^1^H NMR (500 MHz, CDCl_3_) δ 8.54 (dd, *J* = 8.0, 1.8 Hz, 1H), 7.54 (ddd, *J* = 19.5, 8.0, 1.4 Hz, 2H), 7.23–7.13 (m, 9H), 7.09–7.06 (m, 2H), 5.80 (d, *J* = 7.7 Hz, 1H), 4.45 (s, 2H), 3.73 (dddd, *J* = 14.7, 11.8, 8.0, 4.0 Hz, 1H), 1.91–1.88 (m, 1H), 1.67–1.61 (m, 3H), 1.32–1.28 (m, 2H), 1.15–1.09 (m, 3H), 0.88–0.85 (m, 2H). ^13^C{^1^H} NMR (126 MHz, CDCl_3_) δ 166.9, 162.9, 141.2, 137.6, 136.5, 134.3, 132.7, 131.6, 130.6, 128.7, 128.24, 128.18, 128.1, 127.03, 126.95, 125.7, 124.9, 119.7, 50.3, 48.6, 33.1, 25.6, 24.9. HRMS (ESI) *m*/*z*: [M + H]^+^ calcd for C_29_H_29_O_2_N_2_, 437.2224, found, 437.2222.

##### (Z)-2-(1-Benzylidene-3-oxoisoindolin-2-yl)-*N*-(*tert*-butyl)acetamide (**5a**)

It was synthesized according to procedure B on a 0.3 mmol scale to yield final compound **5a** (93 mg, 93%) as a white solid. mp: 174–176 °C. *R*_f_ = 0.40 (30% EtOAc/petroleum ether). ^1^H NMR (500 MHz, CDCl_3_) δ 7.86 (s, 1H), 7.76 (d, *J* = 7.7 Hz, 1H), 7.61 (td, *J* = 1.4, 7.6 Hz, 1H), 7.50 (t, *J* = 7.5 Hz, 1H), 7.39–7.31 (m, 5H), 6.81 (s, 1H), 5.05 (s, 1H), 4.16 (s, 2H), 1.22 (s, 9H). ^13^C{^1^H} NMR (126 MHz, CDCl_3_) δ 169.3, 166.0, 138.5, 135.1, 134.4, 132.4, 129.7, 129.2, 128.5, 127.82, 127.78, 123.7, 119.7, 107.1, 51.4, 46.2, 28.8, 28.7. HRMS (ESI) *m*/*z*: [M + H]^+^ calcd for C_21_H_23_O_2_N_2_, 335.1754, found, 335.1744.

##### (Z)-2-(1-(4-Bromobenzylidene)-3-oxoisoindolin-2-yl)-*N*-(*tert*-butyl)acetamide (**5b**)

It was synthesized according to procedure B on a 0.2 mmol scale to yield final compound **5b** (67 mg, 81%) as a white solid. mp: 184–186 °C. *R*_f_ = 0.25 (20% EA/PE). ^1^H NMR (500 MHz, CDCl_3_) δ 7.86 (d, *J* = 7.6 Hz, 1H), 7.74 (d, *J* = 7.7 Hz, 1H), 7.61 (t, *J* = 7.5 Hz, 1H), 7.53–7.47 (m, 3H), 7.22 (d, *J* = 8.0 Hz, 2H), 6.68 (s, 1H), 5.11 (s, 1H), 4.16 (s, 2H), 1.24 (s, 9H).^13^C{^1^H} NMR (126 MHz, CDCl_3_) δ 169.3, 165.8, 138.4, 135.7, 133.4, 132.6, 131.7, 131.4, 129.5, 127.8, 123.8, 121.9, 119.8, 105.5, 51.6, 46.2, 28.7. HRMS (ESI) *m*/*z*: [M + H]^+^ calcd for C_21_H_22_O_2_N_2_Br, 413.0859, found, 413.0857.

##### (Z)-*N*-(*tert*-Butyl)-2-(1-oxo-3-(pyridin-2-ylmethylene)isoindolin-2-yl)acetamide (**5c**)

It was synthesized according to procedure B on a 0.2 mmol scale to yield final compound **5c** (56 mg, 83%) as a white solid. mp: 212–214 °C. *R*_f_ = 0.26 (50% EtOAc/petroleum ether). ^1^H NMR (500 MHz, CDCl_3_) δ 8.64–8.61 (m, 1H), 7.88 (d, *J* = 7.6 Hz, 1H), 7.79 (d, *J* = 6.9 Hz, 1H), 7.68 (td, *J* = 1.9, 7.7 Hz, 1H), 7.63 (t, *J* = 7.6 Hz, 1H), 7.55–7.50 (m, 1H), 7.40 (d, *J* = 7.7 Hz, 1H), 7.17 (dd, *J* = 4.9, 7.6 Hz, 1H), 6.69 (s, 1H), 5.31 (s, 1H), 4.78 (s, 2H), 1.17 (s, 9H). ^13^C{^1^H} NMR (126 MHz, CDCl_3_) δ 169.3, 167.2, 153.5, 149.3, 139.0, 137.0, 136.6, 132.6, 129.7, 127.8, 125.6, 123.9, 121.9, 119.8, 106.1, 51.2, 47.8, 28.7. HRMS (ESI) *m*/*z*: [M + H]^+^ calcd for C_20_H_22_O_2_N_3_, 336.1707, found, 336.1708.

##### (Z)-*N*-(*tert*-Butyl)-2-(1-oxo-3-((trimethylsilyl)methylene)isoindolin-2-yl)acetamide (**5d**)

It was synthesized according to procedure B on a 0.3 mmol scale to yield final compound **5d** (15 mg, 15%) as a yellow oil. *R*_f_ = 0.28 (EA/PE/DCM = 1:2:1). ^1^H NMR (500 MHz, CDCl_3_) δ 8.35 (t, *J* = 5.1 Hz, 1H), 8.08–8.00 (m, 1H), 7.59–7.51 (m, 1H), 7.47–7.37 (m, 2H), 6.14 (s, 1H), 4.09 (d, *J* = 5.2 Hz, 2H), 1.36 (s, 9H), 0.28 (s, 9H).^13^C{^1^H} NMR (126 MHz, CDCl_3_) δ 167.6, 166.4, 135.0, 134.1, 130.9, 130.2, 129.1, 120.0, 103.0, 102.7, 51.6, 44.9, 28.9. HRMS (ESI) *m*/*z*: [M + H]^+^ calcd for C_16_H_25_ON_5_Si, 331,1823, found, 331,1823.

##### (Z)-2-(1-Benzylidene-5-methoxy-3-oxoisoindolin-2-yl)-*N*-(*tert*-butyl)acetamide (**5e**)

It was synthesized according to procedure B on a 0.3 mmol scale to yield final compound **5e** (98 mg, 90%) as a white solid. mp: 174–176 °C. *R*_f_ = 0.58 (50% EtOAc/petroleum ether). ^1^H NMR (500 MHz, CDCl_3_) δ 7.65 (d, *J* = 8.4 Hz, 1H), 7.39–7.34 (m, 2H), 7.34–7.30 (m, 4H), 7.17 (dd, *J* = 2.4, 8.5 Hz, 1H), 6.69 (s, 1H), 5.00 (s, 1H), 4.15 (s, 2H), 3.89 (s, 3H), 1.23 (s, 9H). ^13^C{^1^H} NMR (126 MHz, CDCl_3_) δ 169.3, 166.1, 161.1, 135.0, 134.6, 131.3, 129.7, 129.3, 128.5, 127.7, 121.2, 121.1, 106.1, 105.9, 55.9, 51.5, 46.4, 28.7. HRMS (ESI) *m*/*z*: [M + H]^+^ calcd for C_22_H_25_O_3_N_2_, 365.1784, found, 365.1781.

##### (Z)-2-(1-Benzylidene-3-oxoisoindolin-2-yl)-*N*-(*tert*-butyl)propenamide (**5f**)

It was synthesized according to procedure B on a 0.3 mmol scale to yield final compound **5f** (75 mg, 72%) as a white solid. mp: 166–168 °C. *R*_f_ = 0.56 (30% EtOAc/petroleum ether). ^1^H NMR (500 MHz, CDCl_3_) δ 7.86 (d, *J* = 7.6 Hz, 1H), 7.77 (d, *J* = 7.7 Hz, 1H), 7.64 (t, *J* = 7.6 Hz, 1H), 7.52 (t, *J* = 7.5 Hz, 1H), 7.38–7.31 (m, 5H), 6.82 (s, 1H), 6.27 (s, 1H), 4.34 (q, *J* = 7.2 Hz, 1H), 1.45 (d, *J* = 7.1 Hz, 3H), 1.36 (s, 9H). ^13^C{^1^H} NMR (126 MHz, CDCl_3_) δ 169.9, 169.6, 139.1, 135.4, 134.0, 132.6, 129.38, 129.36, 128.8, 128.2, 123.4, 119.6, 113.8, 107.7, 55.9, 51.3, 28.8, 15.0. HRMS (ESI) *m*/*z*: [M + H]^+^ calcd for C_22_H_25_O_2_N_2_, 349.1911, found, 349.1909.

##### (Z)-2-(1-Benzylidene-3-oxoisoindolin-2-yl)-*N*-(2,4,4-trimethylpentan-2-yl)acetamide (**5g**)

It was synthesized according to procedure B on a 0.2 mmol scale to yield final compound **5g** (69 mg, 88%) as a white oil. *R*_f_ = 0.15 (1% MeOH/DCM). ^1^H NMR (500 MHz, CDCl_3_) δ 7.88 (d, *J* = 7.6 Hz, 1H), 7.77 (d, *J* = 7.9 Hz, 1H), 7.62 (t, *J* = 6.9 Hz, 1H), 7.51 (t, *J* = 7.5 Hz, 1H), 7.40–7.32 (m, 5H), 6.82 (s, 1H), 5.18 (s, 1H), 4.16 (s, 2H), 1.57 (s, 2H), 1.31 (s, 6H), 0.89 (s, 9H). ^13^C{^1^H} NMR (126 MHz, CDCl_3_) δ 169.3, 165.8, 138.5, 135.0, 134.2, 132.5, 129.7, 129.3, 128.6, 127.9, 127.8, 123.7, 119.7, 107.3, 55.5, 52.4, 46.5, 31.6, 31.5, 28.7. HRMS (ESI) *m*/*z*: [M + H]^+^ calcd for C_25_H_31_O_2_N_2_, 391.2380, found, 391.2382.

##### (Z)-2-(1-Benzylidene-3-oxoisoindolin-2-yl)-*N*-cyclohexylacetamide (**5h**)

It was synthesized according to procedure B on a 0.2 mmol scale to yield final compound **5h** (54 mg, 75%) as a white solid. mp: 206–208 °C. *R*_f_ = 0.15 (30% EtOAc/petroleum ether). ^1^H NMR (500 MHz, CDCl_3_) δ 7.89 (d, *J* = 7.6 Hz, 1H), 7.78 (d, *J* = 7.9 Hz, 1H), 7.65 (td, *J* = 1.3, 7.6 Hz, 1H), 7.53 (td, *J* = 0.9, 7.4 Hz, 1H), 7.39–7.30 (m, 5H), 6.84 (s, 1H), 5.23 (d, *J* = 8.4 Hz, 1H), 4.20 (s, 2H), 3.64 (dtd, *J* = 4.0, 6.9, 10.9 Hz, 1H), 1.83 (dt, *J* = 4.2, 12.1 Hz, 2H), 1.69–1.63 (m, 2H), 1.57 (dt, *J* = 3.9, 12.9 Hz, 1H), 1.36–1.24 (m, 3H), 1.13–1.03 (m, 2H). ^13^C{^1^H} NMR (126 MHz, CDCl_3_) δ 169.4, 166.1, 138.5, 135.0, 134.1, 132.6, 129.5, 129.4, 128.6, 128.0, 127.8, 123.8, 119.7, 107.5, 48.5, 46.1, 33.0, 25.6, 24.9. HRMS (ESI) *m*/*z*: [M + H]^+^ calcd for C_23_H_25_O_2_N_2_, 361.1911, found, 361.1911.

##### (Z)-*N*-Cyclohexyl-2-(1-(4-methylbenzylidene)-3-oxoisoindolin-2-yl)acetamide (**5i**)

It was synthesized according to procedure B on a 0.2 mmol scale to yield final compound **5i** (102 mg, 91%) as a white solid. mp: 213–215 °C. *R*_f_ = 0.74 (50% EtOAc/petroleum ether). ^1^H NMR (500 MHz, CDCl_3_) δ 7.88 (d, *J* = 7.6 Hz, 1H), 7.77 (d, *J* = 7.7 Hz, 1H), 7.64 (td, *J* = 1.3, 7.6 Hz, 1H), 7.52 (td, *J* = 1.0, 7.5 Hz, 1H), 7.22–7.15 (m, 4H), 6.81 (s, 1H), 5.31 (d, *J* = 10.6 Hz, 1H), 4.22 (s, 2H), 3.72–3.62 (m, 1H), 2.37 (s, 3H), 1.84 (dd, *J* = 4.0, 12.5 Hz, 2H), 1.67 (t, *J* = 3.9 Hz, 1H), 1.58 (dt, *J* = 3.9, 13.1 Hz, 1H), 1.37–1.23 (m, 3H), 1.15–0.99 (m, 3H). ^13^C{^1^H} NMR (126 MHz, CDCl_3_) δ 169.5, 166.3, 138.6, 137.9, 134.6, 132.6, 131.0, 129.4, 129.34, 129.26, 127.7, 123.7, 119.7, 107.8, 48.4, 46.2, 33.0, 25.6, 24.9, 21.5. HRMS (ESI) *m*/*z*: [M + H]^+^ calcd for C_24_H_27_O_2_N_2_, 375.2067, found, 375.2063.

##### (Z)-2-(1-(4-Bromobenzylidene)-3-oxoisoindolin-2-yl)-*N*-cyclohexylacetamide (**5j**)

It was synthesized according to procedure B on a 0.2 mmol scale to yield final compound **5j** (110 mg, 84%) as a white solid. mp: 214–216 °C. *R*_f_ = 0.52 (30% EtOAc/petroleum ether). ^1^H NMR (500 MHz, CDCl_3_) δ 7.88 (dt, *J* = 1.0, 7.6 Hz, 1H), 7.77–7.74 (m, 1H), 7.64 (td, *J* = 1.3, 7.6 Hz, 1H), 7.53 (td, *J* = 1.0, 7.5 Hz, 1H), 7.49 (d, *J* = 8.4 Hz, 2H), 7.22–7.19 (m, 2H), 6.70 (s, 1H), 5.28 (d, *J* = 8.2 Hz, 1H), 4.19 (s, 2H), 3.65 (tdt, *J* = 4.0, 7.9, 10.9 Hz, 1H), 1.84 (dt, *J* = 3.9, 12.3 Hz, 2H), 1.66 (dt, *J* = 3.9, 14.2 Hz, 2H), 1.58 (dt, *J* = 3.8, 12.9 Hz, 1H), 1.31 (ddt, *J* = 4.2, 13.2, 14.8 Hz, 2H), 1.15–1.00 (m, 3H). ^13^C{^1^H} NMR (126 MHz, CDCl_3_) δ 169.4, 165.9, 138.4, 135.6, 133.2, 132.7, 131.7, 131.2, 129.6, 127.7, 123.8, 122.1, 119.7, 105.9, 48.6, 46.0, 33.0, 25.6, 24.9. HRMS (ESI) *m*/*z*: [M + H]^+^ calcd for C_23_H_24_O_2_N_2_Br, 439.1016, found, 439.1014.

##### N-Cyclohexyl-2-(1-oxo-3-(pyridin-2-ylmethylene)isoindolin-2-yl)acetamide (Z:E=51:49) (**5k**)

It was synthesized according to procedure B on a 0.3 mmol scale to yield final compound **5k** (79 mg, 73%) as a white solid. mp: 224-224 °C. *R*_f_ = 0.24 (50% EtOAc/petroleum ether). (Z) ^1^H NMR (500 MHz, CDCl_3_) δ 8.60 (d, *J* = 5.5 Hz, 1H), 7.91–7.85 (m, 1H), 7.80 (d, *J* = 7.7 Hz, 1H), 7.70–7.60 (m, 2H), 7.54 (t, *J* = 7.5 Hz, 1H), 7.39 (d, *J* = 7.8 Hz, 1H), 7.26–7.23 (m, 1H), 7.16 (dd, *J* = 7.7, 4.7 Hz, 1H), 6.69 (s, 1H), 5.53 (d, *J* = 8.4 Hz, 1H), 4.77 (s, 2H), 3.66 (tdt, *J* = 11.3, 7.7, 3.9 Hz, 1H), 1.65–1.52 (m, 5H), 1.34–1.26 (m, 2H), 1.11–0.92 (m, 3H). ^13^C{^1^H} NMR (126 MHz, CDCl_3_) δ 169.4, 167.4, 153.2, 149.3, 139.0, 136.8, 136.6, 132.7, 129.8, 127.7, 125.5, 123.9, 122.0, 119.8, 106.4, 48.2, 47.8, 33.0, 25.6, 24.9. (E) ^1^H NMR (500 MHz, CDCl_3_) δ 8.73 (d, *J* = 5.0 Hz, 1H), 8.57–8.52 (m, 1H), 7.95–7.85 (m, 2H), 7.74 (td, *J* = 7.7, 2.0 Hz, 1H), 7.57–7.51 (m, 2H), 7.39 (d, *J* = 8.0 Hz, 1H), 6.53 (s, 1H), 5.88 (d, *J* = 8.5 Hz, 1H), 4.55 (s, 2H), 3.78 (tdt, *J* = 11.8, 8.0, 4.0 Hz, 1H), 1.85–1.74 (m, 2H), 1.65–1.52 (m, 3H), 1.34–1.26 (m, 2H), 1.11–0.92 (m, 3H). ^13^C{^1^H} NMR (126 MHz, CDCl_3_) δ 167.2, 166.6, 154.0, 149.4, 138.4, 136.8, 135.0, 132.9, 130.3, 129.8, 126.2, 125.9, 123.5, 122.5, 111.7, 48.6, 44.6, 33.0, 25.4, 24.9. HRMS (ESI) *m*/*z*: [M + H]^+^ calcd for C_22_H_24_O_2_N_3_, 362.1863, found, 362.1862.

##### (Z)-*N*-Cyclohexyl-2-(1-oxo-3-(pyridin-2-ylmethylene)isoindolin-2-yl)acetamide (**5l**)

It was purified from **5k** on a 0.2 mmol scale to yield final compound **5l** (24 mg, 33%) as a white solid. mp: 232-234 °C. *R*_f_ = 0.40 (25% EtOAc/DCM). ^1^H NMR (500 MHz, CDCl_3_) δ 8.60 (d, *J* = 3.7 Hz, 1H), 7.89 (d, *J* = 7.6 Hz, 1H), 7.80 (d, *J* = 7.7 Hz, 1H), 7.70–7.61 (m, 2H), 7.54 (t, *J* = 7.5 Hz, 1H), 7.38 (d, *J* = 7.8 Hz, 1H), 7.16 (dd, *J* = 7.6, 4.9 Hz, 1H), 6.69 (s, 1H), 5.52 (d, *J* = 8.2 Hz, 1H), 4.78 (s, 2H), 3.66 (tdt, *J* = 11.3, 8.1, 4.0 Hz, 1H), 1.76 (d, *J* = 4.1 Hz, 2H), 1.64–1.49 (m, 3H), 1.33–1.20 (m, 2H), 1.06 (tt, *J* = 12.4, 3.5 Hz, 1H), 1.02–0.91 (m, 2H). ^13^C{^1^H} NMR (126 MHz, CDCl_3_) δ 169.4, 167.4, 153.2, 149.4, 139.0, 136.9, 136.6, 132.7, 129.8, 127.7, 125.5, 123.9, 122.0, 119.8, 106.4, 48.2, 47.8, 33.0, 25.6, 24.9. HRMS (ESI) *m*/*z*: [M + H]^+^ calcd for C_22_H_24_O_2_N_3_, 362.1863, found, 362.1862.

##### (*E*)-*N*-Cyclohexyl-2-(1-oxo-3-(pyridin-2-ylmethylene)isoindolin-2-yl)acetamide (**5m**)

It was purified from **5k** on a 0.2 mmol scale to yield final compound **5m** (22 mg, 30%) as a white solid. mp: 228-230 °C. *R*_f_ = 0.29 (25% EtOAc/DCM). ^1^H NMR (500 MHz, CDCl_3_) δ 8.74 (d, *J* = 3.9 Hz, 1H), 8.58 (dd, *J* = 6.2, 1.5 Hz, 1H), 7.95–7.85 (m, 1H), 7.75 (td, *J* = 7.7, 1.9 Hz, 1H), 7.61–7.50 (m, 2H), 7.41 (d, *J* = 8.0 Hz, 1H), 6.54 (s, 1H), 5.82 (d, *J* = 8.0 Hz, 1H), 5.30 (s, 1H), 4.56 (s, 2H), 3.78 (tdt, *J* = 10.7, 8.1, 4.1 Hz, 1H), 1.86 (dd, *J* = 12.5, 3.5 Hz, 2H), 1.69–1.61 (m, 2H), 1.55 (d, *J* = 3.9 Hz, 1H), 1.35–1.26 (m, 2H), 1.14–1.01 (m, 3H).^13^C{^1^H} NMR (126 MHz, CDCl_3_) δ 167.2, 166.7, 154.0, 149.5, 138.4, 136.9, 135.0, 132.9, 130.4, 129.7, 126.2, 125.9, 123.6, 122.5, 111.7, 48.7, 44.7, 33.0, 25.5, 24.9. HRMS (ESI) *m*/*z*: [M + H]^+^ calcd for C_22_H_24_O_2_N_3_, 362.1863, found, 362.1862.

##### (Z)-2-(1-Benzylidene-3-oxoisoindolin-2-yl)-*N*-phenethylacetamide (**5n**)

It was synthesized according to procedure B on a 0.2 mmol scale to yield final compound **5n** (61 mg, 80%) as a white solid. mp: 180–182 °C. *R*_f_ = 0.76 (20% EtOAc/petroleum ether). ^1^H NMR (500 MHz, CDCl_3_) δ 7.88 (d, *J* = 7.6 Hz, 1H), 7.76 (d, *J* = 7.7 Hz, 1H), 7.65 (td, *J* = 1.4, 7.6 Hz, 1H), 7.53 (td, *J* = 0.8, 7.6 Hz, 1H), 7.36–7.28 (m, 3H), 7.24–7.14 (m, 5H), 7.08 (d, *J* = 6.5 Hz, 2H), 6.80 (s, 1H), 5.41 (t, *J* = 5.8 Hz, 1H), 4.21 (s, 2H), 3.36–3.30 (m, 2H), 2.70 (t, *J* = 7.1 Hz, 2H). ^13^C{^1^H} NMR (126 MHz, CDCl_3_) δ 169.0, 167.0, 138.7, 138.3, 134.8, 134.0, 132.6, 129.5, 129.4, 128.8, 128.7, 128.5, 128.0, 127.7, 126.6, 123.7, 119.7, 107.5, 45.7, 40.8, 35.5. HRMS (ESI) *m*/*z*: [M + H]^+^ calcd for C_25_H_23_O_2_N_2_, 383.1754, found, 383.1755.

##### (Z)-2-(1-(4-Bromobenzylidene)-3-oxoisoindolin-2-yl)-*N*-(2-methoxyphenyl)acetamide (**5o**)

It was synthesized according to procedure B on a 0.3 mmol scale to yield final compound **5o** (123 mg, 89%) as a white solid. mp: 185–186 °C. *R*_f_ = 0.50 (30% EtOAc/petroleum ether). ^1^H NMR (500 MHz, CDCl_3_) δ 8.21 (d, *J* = 8.0 Hz, 1H), 7.92 (d, *J* = 7.6 Hz, 1H), 7.78 (d, *J* = 7.7 Hz, 1H), 7.65 (t, *J* = 7.6 Hz, 1H), 7.55 (t, *J* = 7.5 Hz, 1H), 7.45 (s, 1H), 7.37 (d, *J* = 8.2 Hz, 2H), 7.15 (d, *J* = 8.0 Hz, 2H), 7.03 (t, *J* = 7.8 Hz, 1H), 6.91 (t, *J* = 7.8 Hz, 1H), 6.84 (d, *J* = 8.2 Hz, 1H), 6.73 (s, 1H), 4.41 (s, 2H), 3.81 (d, *J* = 1.0 Hz, 3H).^13^C{^1^H} NMR (126 MHz, CDCl_3_) δ 169.0, 164.6, 147.6, 138.2, 135.6, 133.3, 132.7, 131.6, 131.2, 129.6, 127.9, 127.1, 124.0, 123.9, 122.1, 121.1, 119.8, 119.5, 109.9, 105.8, 56.0, 46.3. HRMS (ESI) *m*/*z*: [M + H]^+^ calcd for C_24_H_20_O_3_N_2_Br, 463.0652, found, 463.0652.

##### 2-(3-([1,1′-Biphenyl]-4-yl)-1-oxoisoquinolin-2(1H)-yl)-*N*-(*tert*-butyl)acetamide (**6a**)

It was synthesized according to procedure C on a 0.15 mmol scale to yield final compound **6a** (52 mg, 85%) as a white solid. mp: 194–196 °C. *R*_f_ = 0.62 (50% EtOAc/petroleum ether). ^1^H NMR (500 MHz, CDCl_3_) δ 8.45 (d, *J* = 8.0 Hz, 1H), 7.69–7.62 (m, 5H), 7.57–7.46 (m, 6H), 7.39 (t, *J* = 7.3 Hz, 1H), 6.54 (s, 1H), 5.88 (s, 1H), 4.48 (s, 2H), 1.34 (s, 9H). ^13^C{^1^H} NMR (126 MHz, CDCl_3_) δ 167.1, 163.6, 143.7, 142.2, 140.3, 136.7, 134.6, 132.9, 129.9, 129.1, 128.2, 127.9, 127.4, 127.3, 126.9, 126.1, 124.9, 108.4, 51.6, 50.6, 28.8. HRMS (ESI) *m*/*z*: [M + H]^+^ calcd for C_27_H_27_O_2_N_2_, 411,2067, found, 411,2062.

##### (Z)-*N*-(*tert*-Butyl)-2-(1-((4′-methoxy-[1,1′-biphenyl]-4-yl)methylene)-3-oxoisoindolin-2-yl)acetamide(**6b**)

It was synthesized according to procedure C on a 0.15 mmol scale to yield final compound **6b** (60 mg, 91%) as a light yellow oil. *R*_f_ = 0.76 (50% EtOAc/petroleum ether). ^1^H NMR (500 MHz, CDCl_3_) δ 7.89 (dd, *J* = 7.6, 0.9 Hz, 1H), 7.78 (d, *J* = 7.8 Hz, 1H), 7.67–7.44 (m, 7H), 7.39 (d, *J* = 8.0 Hz, 2H), 7.00 (d, *J* = 8.7 Hz, 2H), 6.82 (s, 1H), 5.05 (s, 1H), 4.26 (s, 2H), 3.86 (s, 3H), 1.23 (s, 9H). ^13^C{^1^H} NMR (126 MHz, CDCl_3_) δ 169.4, 166.1, 159.6, 140.2, 138.6, 135.2, 132.9, 132.7, 132.5, 130.2, 129.2, 127.8, 126.6, 123.7, 119.7, 114.5, 106.9, 55.5, 51.5, 46.4. HRMS (ESI) *m*/*z*: [M + H]^+^ calcd for C_28_H_29_O_3_N_2_, 441,2173, found, 441,2172.

#### Methyl(2-(1-benzylidene-3-oxoisoindolin-2-yl)acetyl) glycinate (*Z*/*E* = 26:74) (**6d**)

It was synthesized according to procedure C on a 0.5 mmol scale to yield carboxylic acid **6c**, which was suspended in methanol and cooled to 0 °C. Then, SOCl_2_ was added dropwise and the mixture was further stirred for 4 h. After the reaction, the solvent was removed under reduced pressure and the crude product was extracted with water and DCM, the combined organic layer was dried over Na_2_SO_4_, the solvent was removed, and the crude was purified to give final compound **3d** (92 mg, 53%). White solid. mp: 149–150 °C. *R*_f_ = 0.16 (50% EtOAc/petroleum ether). (Z) ^1^H NMR (500 MHz, CDCl_3_) δ 7.86 (t, *J* = 7.3 Hz, 3H), 7.77 (d, *J* = 7.8 Hz, 1H), 7.63 (t, *J* = 7.6 Hz, 1H), 7.51 (t, *J* = 7.5 Hz, 1H), 7.40–7.36 (m, 3H), 6.86 (s, 1H), 5.97 (t, *J* = 5.2 Hz, 1H), 4.28 (s, 2H), 3.90 (d, *J* = 5.1 Hz, 2H), 3.73 (s, 3H). ^13^C{^1^H} NMR (126 MHz, CDCl_3_) δ 170.1, 169.3, 167.4, 138.4, 134.9, 134.4, 134.1, 132.4, 129.4, 128.5, 128.1, 123.8, 123.7, 119.8, 107.7, 52.5, 45.7, 41.3. (E) ^1^H NMR (500 MHz, CDCl_3_) δ 7.44 (dt, *J* = 13.3, 7.6 Hz, 5H), 7.36–7.30 (m, 4H), 6.64 (s, 1H), 6.63–6.60 (m, 1H), 4.61 (s, 2H), 4.06 (d, *J* = 5.5 Hz, 2H), 3.71 (s, 3H). ^13^C{^1^H} NMR (126 MHz, CDCl_3_) δ 169.9, 168.0, 166.9, 135.8, 135.3, 134.7, 132.7, 129.7, 129.6, 128.8, 128.2, 127.7, 123.7, 123.5, 112.4, 52.5, 43.9, 41.2. HRMS (ESI) *m*/*z*: [M + H]^+^ calcd for C_20_H_19_O_4_N_2_, 351,1345, found, 351,1332.

## Supporting Information Available

Experimental procedure and ^1^H and ^13^C{^1^H} NMR spectra
for all compounds with the X-ray crystallographic data for **3e, 3i, 5a**, ^1^H-^13^C HMBC NMR spectra for **3a** and **5a** (PDF)
