# Supplementary material for: Access to Isoquinolin-2(1H)-yl-acetamides and Isoindolin-2-yl-acetamides from a Common MCR Precursor
Source: J Org Chem. 2022 Oct 25;87(21):14463–75. doi: 10.1021/acs.joc.2c01905 (PMC9639002; doi:10.1021/acs.joc.2c01905)
Supplement: Supplementary file 1 — jo2c01905_si_001.pdf [file jo2c01905_si_001.pdf]

Supporting Information

Access to Isoquinolin-2(1*H*)-yl-acetamides and Isoindolin-2-yl-acetamides From a Common MCR Precursor

Xin Li,[†a] Qian Wang,[†a] Qiang Zheng[†a], Katarzyna Kurpiewska,[b] Justyna Kalinowska-Tluscik,[b] and Alexander Dömling[*a]

[a] University of Groningen, Department of Drug Design, A. Deusinglaan 1, 9713 AV, Groningen, The Netherlands.

[b] Jagiellonian University, Faculty of Chemistry, Department of Crystal Chemistry and Crystal, Physics, Biocrystallography Group, Gronostajowa 2, 30-387 Krakow, Poland.

[†]Xin Li and Qian Wang contributed equally to this work.

* Corresponding author E-mail: a.s.s.domling@rug.nl, www.drugdesign.nl

Contents:

1. X-ray Crystallographic Data for **3e**, **3i**, **5a**--S2–S4

2. Optimization of Reaction Conditions for Isoindolin-2-yl-acetamide------------------------------S5

3. Gram Scale synthesis of **3a** and **5a**---S6

4. Structures of all the Trace and ND products in this work---S6

5. HMBC correlations of **3a** and **5a** ($^2J_{CH}$ and $^3J_{CH}$)---S7

6. Plausible mechanisms---S8

7. The ^{1}H-NMR and ^{13}C{^{1}H} NMR spectrum of final compounds---------------------------S9–S52

8. References---S53

Crystal sample preparation:

Recrystallization in EtOH or MeOH

20-30 mg sample powder was added into the 3 mL vial with around 1 mL EtOH or MeOH, sealed the vial and heated at 70 °C for several minutes, filtered the solution and removed undissolved material, then transfer the filtrate to a new 3 mL glass vial , cap the vial, and kept still for 2-4 weeks in a dark environment.

Crystal structure determination

X-ray diffraction data for single crystals of compounds **3e, 3i** and **5a** were collected using XtalLAB Synergy-S four circle diffractometer with a mirror monochromator and a microfocus CuKα radiation source (λ = 1.5418 Å). The CryoStream cryostat system was used to allow low-temperature experiments, performed at 100(2) K. The obtained data sets were processed with CrysAlisPro software.[1] The phase problem was solved with direct methods using SIR2014.[2] Parameters of the obtained models were refined by full-matrix least-squares on F^2 using SHELXL-2014/6.[3] Calculations were performed using WinGX integrated system (ver. 2014.1).[4] Figures were prepared with Mercury 4.0 software.[5]

All non-hydrogen atoms were refined anisotropically. All hydrogen atoms attached to carbon atoms and N12 in structure **5a** were positioned with the idealised geometry and refined using the riding model with the isotropic displacement parameter $U_{iso}[H] = 1.2\ U_{eq}[C]$ for all but methyl group, for which $U_{iso}[H] = 1.5\ U_{eq}[C]$ was applied. Hydrogen atoms bound to N13 in structures **3e** and **3i** were located on the Fourier difference map and refined with no restraints on U_{iso} parameter. Crystal data and refinement results are shown in Table S1.

Crystallographic data have been deposited with the Cambridge Crystallographic Data Centre (CCDC) as supplementary publication nos.: CCDC 2168278 (**3e**), CCDC 2168279 (**3i**) and CCDC 2168280 (**5a**). Copies of the data can be obtained, free of charge, on application to CCDC, 12 Union Road, Cambridge CB2 1EZ, UK, (e-mail: deposit@ccdc.cam.ac.uk).

Crystal structure description

Compound **3e** crystallises in the centrosymmetric space group $P2_1/c$ while compounds **3i** and **5a** in non-centrosymmetric space groups P $P4_12_12$ and $Pna2_1$, respectively. The observed chirality of the last two mentioned structures is related to the intermolecular interactions motifs observed in the crystal lattice. The atoms of amide fragments of neighbouring molecules form N-H…O hydrogen bond, helical-like chain motif, propagating along [100] and [001] directions for structures **3i** and **5a**, respectively. This helical arrangement is the main cause of the chirality of the crystal.

The asymmetric unit of **3i** and **5a** consists of a single molecule of the investigated compound (Figure S1). The centrosymmetric structure **3e** is a solvate, with a highly disordered ethanol molecule observed in the crystal lattice. (Figure S1). The disorder is complicated and based on two independent factors: the ethanol molecule is located in a special position, corresponding to the inversion centre, and additionally, it adopts two alternative conformations (molecules E and F in Figure S1). The hydroxyl group of the solvent is a donor in the stabilizing O-H…O hydrogen bond, with the acceptor located at the carbonyl oxygen atom of the isocarbostyril moiety. Due to the mentioned intermolecular interaction and the alternative special arrangement of the ethanol molecule, right- and left-handed helical motifs are observed in the crystal, formed by the corresponding N-H…O chains. This is the main reason why compound **3e** crystallises in the centrosymmetric space group. The phenyl substituent in the proximity of amide bond in structures **3e** and **5a** cases the steric hindrance, weakening the N-H…O interaction in these structures. Additionally, the weaker hydrogen bond observed for **3e** and **5a** crystal structures may also correspond to the intramolecular contact of the π-electrons of the closest phenyl substituent with p-electrons resonating in the peptide bond. This stabilizes a particular geometry of the molecule with a high impact on interactions formation and their strength.

Acknowledgements

The crystal structure analysis was performed on the equipment purchased thanks to the financial support of the Ministry of Science and Higher Education, Warsaw, Poland [grant number 6903/IA/SP/2018].

Table S1. Crystal data and final refinement results for structures **3e**, **3i** and **5a**

	**3e**	**3i**	**5a**
Empirical moiety formula	$C_{27} H_{26} N_2 O_2, C_2 H_6 O$	$C_{15} H_{18} N_2 O_2$	$C_{21} H_{22} N_2 O_2$
Formula weight [g/mol]	433.53	258.31	334.40
Crystal system	Monoclinic	Tetragonal	Orthorhombic
Space group	$P2_1/c$	$P4_1 2_1 2$	$Pn a 2_1$
Unite cell dimensions	a = 12.3323(2) Å<br>b = 19.6034(3) Å<br>c = 9.6200(1) Å<br>α=90°<br>β=92.868(1)°<br>γ=90°	a = 12.2394(1) Å<br>b = 12.2394(1) Å<br>c = 18.3566(3) Å<br>α=90°<br>β=90°<br>γ=90°	a = 9. 7308(1) Å<br>b = 14.6973(2) Å<br>c = 12.4811(1) Å<br>α=90°<br>β=90°<br>γ=90°
Volume [$Å^3$]	2322.77(6)	2749. 87 (6)	1787.00(3)
Z	4	8	4
D_{calc} [Mg/m^3]	1.240	1.248	1.244
μ [mm^{-1}]	0.627	0.673	0.640
F(000)	924	1104	712
Crystal size [mm^3]	0.4 x 0.05 x 0.02	0.3 x 0.2 x 0.1	0.6 x 0.3 x 0.1
Θ range	3.59° to 73.30°	4.34° to 76.35°	4.65° to 76.790°
Index ranges	$-15 \le h \le 15$,<br>$-23 \le k \le 23$,<br>$-11 \le l \le 8$	$-15 \le h \le 15$,<br>$-15 \le k \le 15$,<br>$-21 \le l \le 22$	$-10 \le h \le 12$,<br>$-18 \le k \le 18$,<br>$-15 \le l \le 15$
Refl. collected	16837	79948	47397
Independent reflections	4484<br>[R(int) = 0.0435]	2864<br>[R(int) = 0.1214]	3619<br>[R(int) = 0.1468]
Completeness [%] to Θ	99.6 (Θ 67.7°)	100.0 (Θ 67.7°)	100.0 (Θ 67.7°)
Absorption correction	Multi-scan	Multi-scan	Multi-scan
Tmin. and Tmax.	0.779 and 1.000	0.335 and 1.000	0.521 and 1.000
Data/ restraints/parameters	4484 / 6 / 320	2864 / 0 / 180	3619 / 1 / 230
GooF on F2	1.058	1.143	1.114
Final R indices [I>2sigma(I)]	R1= 0.0479,<br>wR2= 0.1142	R1= 0.0391,<br>wR2= 0.0994	R1= 0.0441,<br>wR2= 0.1189
R indices (all data)	R1= 0.0565,<br>wR2= 0.1188	R1= 0.0429,<br>wR2= 0.1150	R1= 0.0481,<br>wR2= 0.1321
$\Delta\rho_{max}$, $\Delta\rho_{min}$ [$e \cdot Å^{-3}$]	0.286 and -0.322	0.206 and -0.196	0.248 and -0.214
Absolute structure parameter	-	-0.11(8)	-0.02(12)

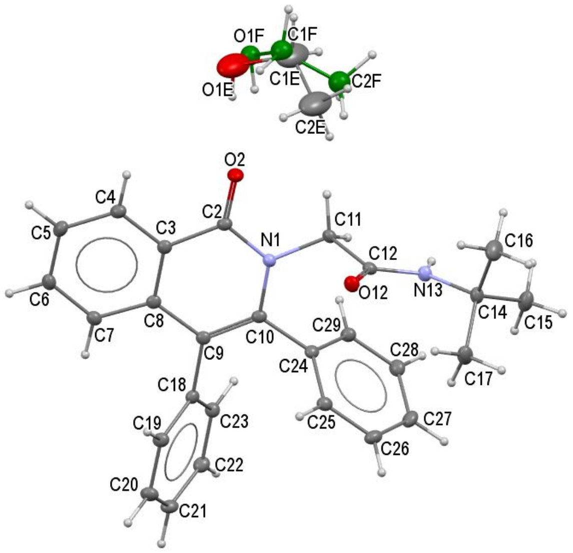

3e

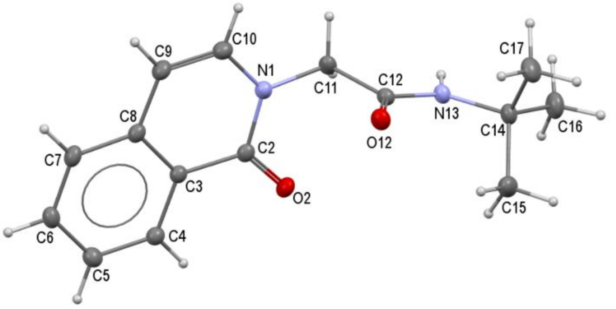

3i

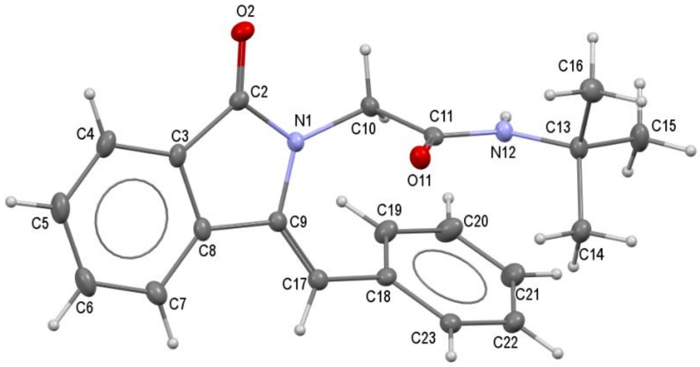

5a

Figure S1. Asymmetric units in crystal structures of compounds **3e**, **3i** and **5a**, presenting the molecular geometry and the atom labelling scheme. Compound **3e** crystallise in a solvate form, with a highly disordered ethanol molecule in the crystal lattice (alternative conformations are marked with E and F letters added to the particular labels; conformer F is shown in ball&stick representation and coloured in green for figure clarity). Displacement ellipsoids of non-hydrogen atoms are drawn at the 30% probability level. H atoms are presented as small spheres with an arbitrary radius.

Table S2. Optimization of Reaction Conditions for Isoindolin-2-yl-acetamide

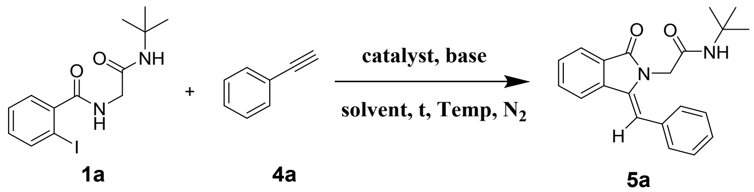

Entry	Catalyst(0.2)	base	solvent	time	T(^{O}C)	Yield(%)
1	$Cu(OAc)_2$	CS_2CO_3	PEG400	12h	90	45[a]
2	$Cu(OAc)_2$	Na_2CO_3	PEG400	12h	90	**68**[b]
3	$Cu(OAc)_2$	K_2CO_3	PEG400	12h	90	**72**[b]
4	$Cu(OAc)_2$	K_2CO_3	DMF	12h	90	**18**[b]
5	$Cu(OAc)_2$	K_2CO_3	DMSO	12h	90	**42**[b]
6	$Cu(OAc)_2$	K_2CO_3	i-PrOH	12h	90	**25**[b]
7	$Cu(OAc)_2$	K_2CO_3	EtOH	12h	90	**34**[b]
8	$Cu(OAc)_2$	K_2CO_3	PEG400	12h	80	**76**[b]
9	$Cu(OAc)_2$	K_2CO_3	PEG400	12h	100	**81**[b]
10	$Cu(OAc)_2$	K_2CO_3	PEG400	2h	100	**90**[a]
11	$Cu(OAc)_2$	CS_2CO_3	PEG400	4h	100	**48**[b]
12	$Cu(OAc)_2$	CS_2CO_3	PEG400	8h	100	**56**[b]
13	CuBr	K_2CO_3	PEG400	2h	100	**93%**[a]
14	CuCl	K_2CO_3	PEG400	2h	100	**72**[b]
15	CuI	K_2CO_3	PEG400	2h	100	**75**[b]

Table S2. [a]Reaction conditions: **1a** (0.3 mmol), **4a** (0.45 mmol), catalyst (20 mmol %), base (0.6 mmol), solvent (2 mL), Isolated yields. [b]Reaction conditions: **1a** (0.1 mmol), **2a** (0.15 mmol), catalyst (10 mmol %), base (0.2 mmol), solvent (1 mL), HPLC yields.

A

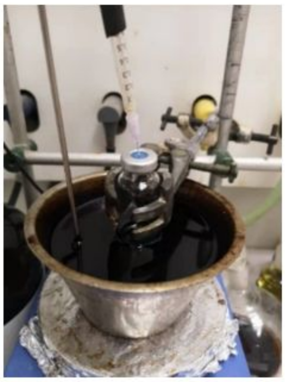

B

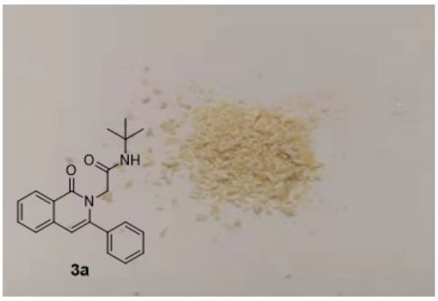

C

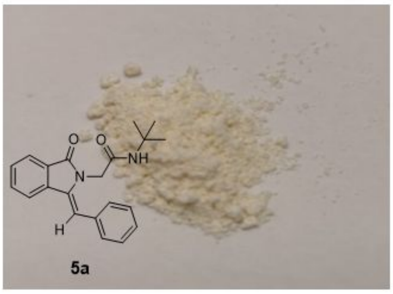

Figure S2. Gram-scale synthesis procedure of **3a** and **5a. (A)** Reaction setup of the Gram-scale synthesis. **(B)** View of **3a**, 8mmol, 1.14g, 43%. **(C)** View of **5a**, 8mmol, 1.74g, 64%.

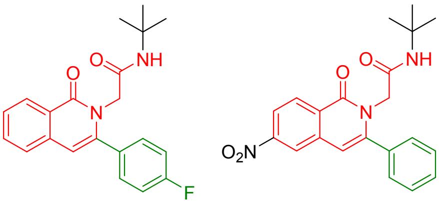

3q, trace **3r, N.D.**

Figure S3. Structures of the trace and not determined products in this work.

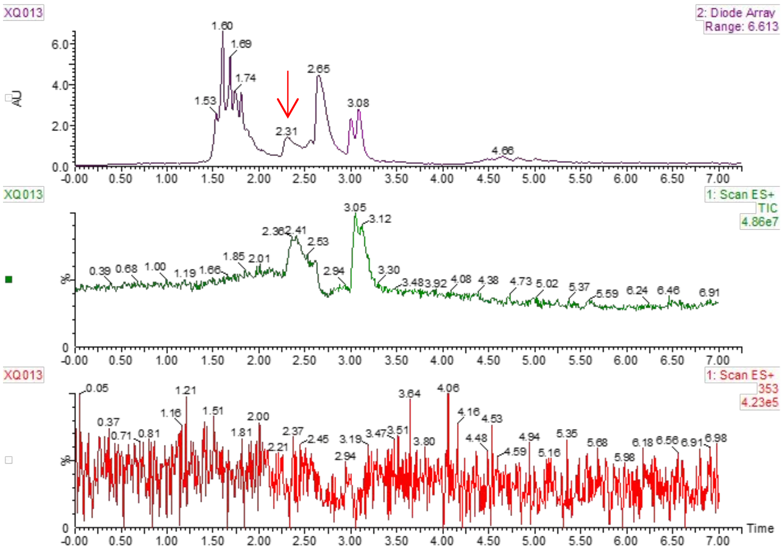

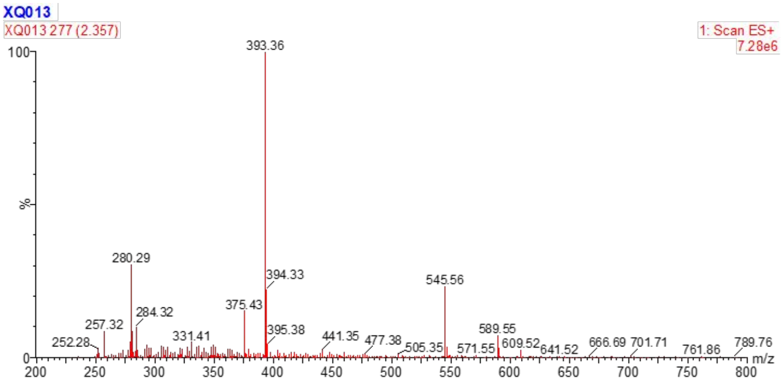

Figure S4. MS spectrum of trace compound **3q** (XQ013).

The structures of **3a** and **5a** were further verified by two dimensional HMBC NMR experiments (Figure 3). In isoquinolin-2(1*H*)-yl-acetamide **3a,** two 17a-protons (4.43 ppm) correlate with C-1 (163.08 ppm, $^3J_{CH}$) and C-3 (143.52 ppm, $^3J_{CH}$) as well as C-18 (166.99 pm, $^2J_{CH}$), furthermore, C-3 was assigned by three cross-peaks with 4a-H (6.48 ppm, $^2J_{CH}$), 12a-H (7.45 ppm, $^3J_{CH}$), 16a-H (7.45 ppm, $^3J_{CH}$). Although the 17b-protons (4.17 ppm) of isoindolin-2-yl-acetamide **5a** could also achieve three ^{1}H-^{13}C correlations with C-1 (169.17 ppm, $^3J_{CH}$) and C-3 (134.83 ppm, $^3J_{CH}$) as well as C-18 (165.69 pm, $^2J_{CH}$), only two adddtional cross-peaks were observed between C-3 (134.83 ppm, $^3J_{CH}$) and 4b-H (7.76 ppm, $^3J_{CH}$) and 10b-H (6.82 ppm, $^2J_{CH}$) (Figure S5).

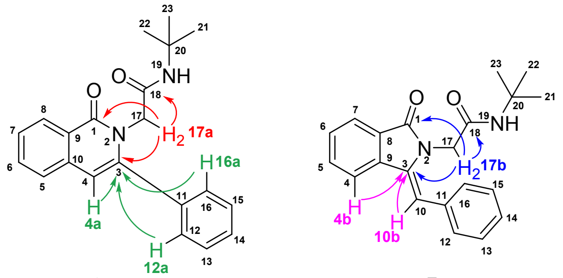

3a **5a**

Figure S5. HMBC correlations of **3a** and **5a** ($^2J_{CH}$ and $^3J_{CH}$).

Scheme S1. Plausible Mechanisms

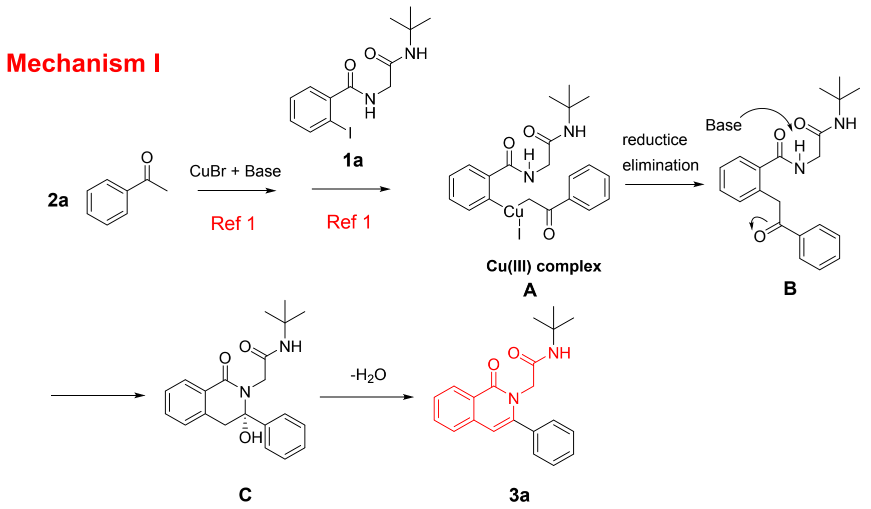

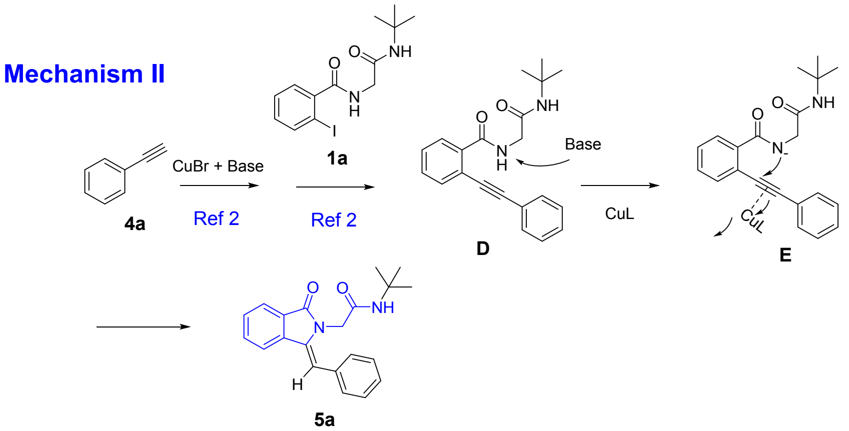

Mechanism I: According to our previous work (ref 1),[6] the Cu(III) complex **A** is first generated from acetophenone **2a** and with 2-iodobenzamide **1a** in the presence of CuBr and Cs_2CO_3. Then the reductive elimination of complex **A** resulted in compound **B**, which can undergo the intramolecular cyclization reaction and beta-elimination, finally yielding product **3a**.

Mechanism II: Similar to the work reported by Li (ref 2),[7] The alternative reaction mechanism II starts from the formation of the intermediate **D** via the intermolecular addition reaction assisted by CuBr, base and ligand PEG.[8] Then, D is converted to the Cu complex **E**, which undergo the 5-exo-dig cyclization to afford **5a**.

^{1}H NMR spectrum of **1a** (500 MHz, $CDCl_3$)

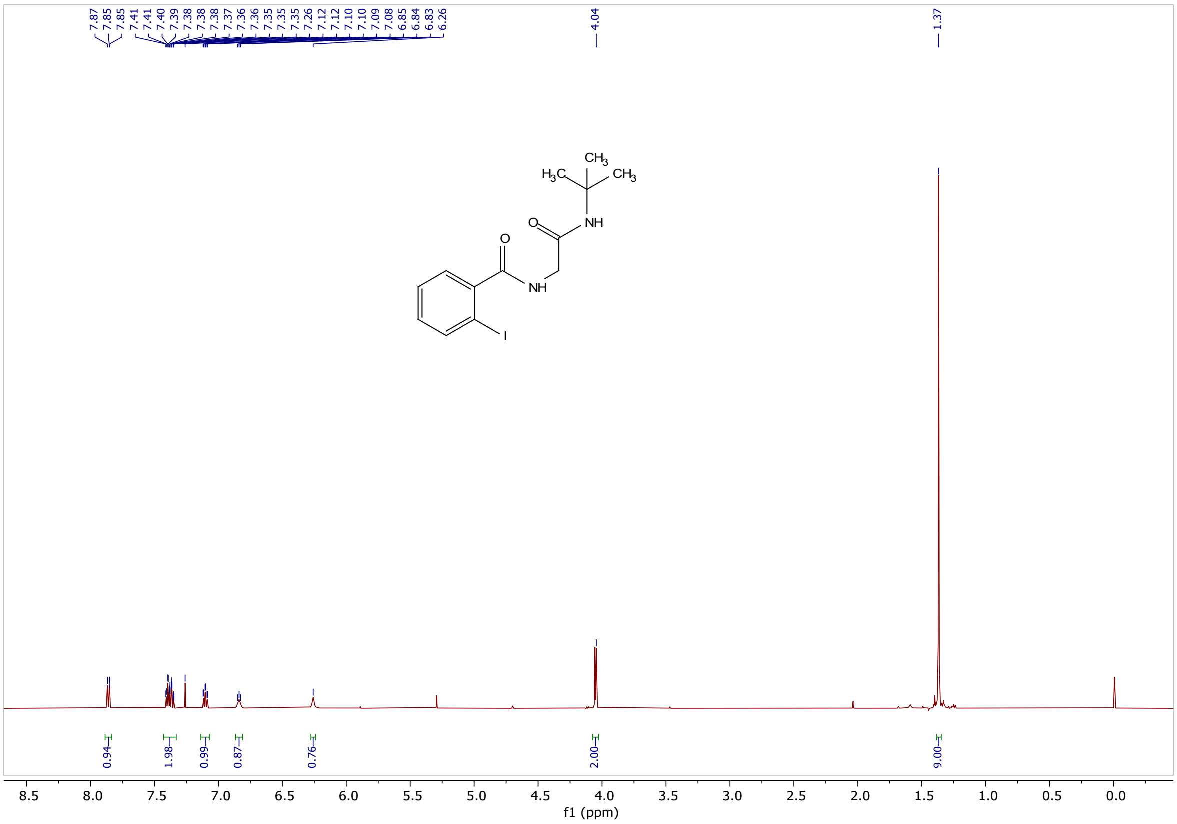

^{1}H NMR spectrum of **1d** (500 MHz, $CDCl_3$)

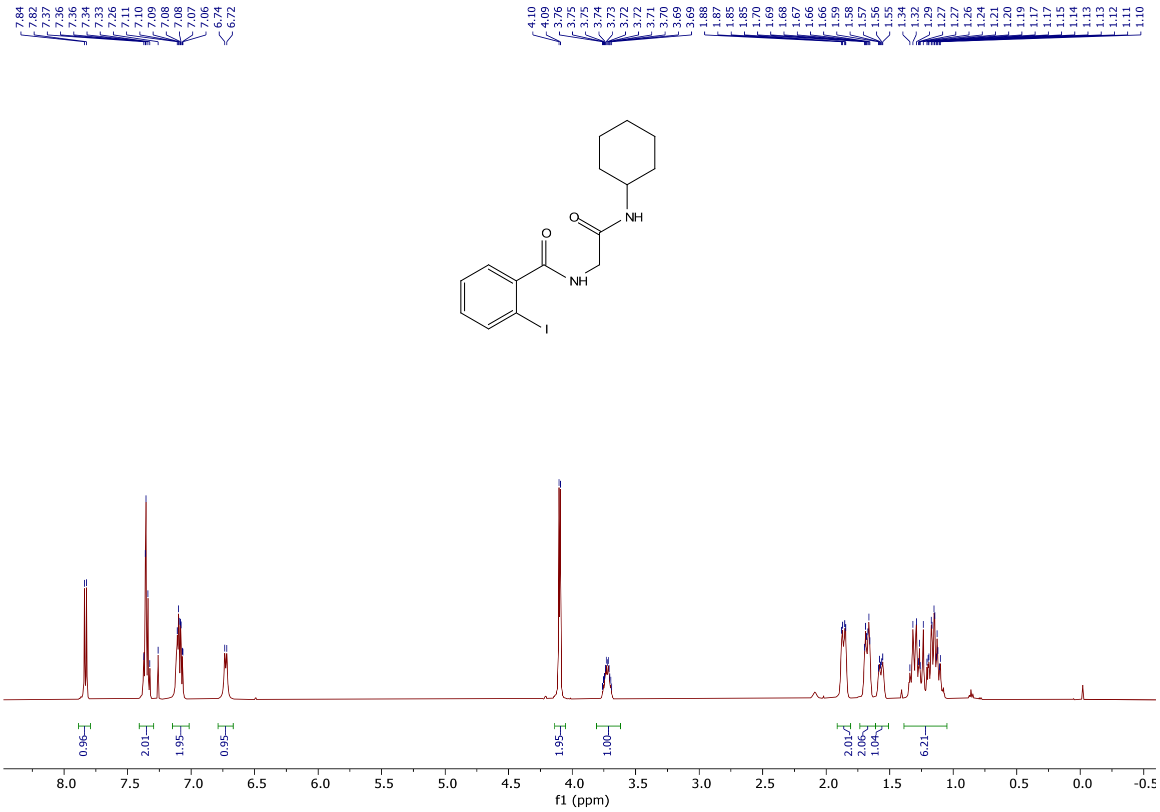

^{1}H NMR spectrum of **1f** (500 MHz, CDCl$_3$)

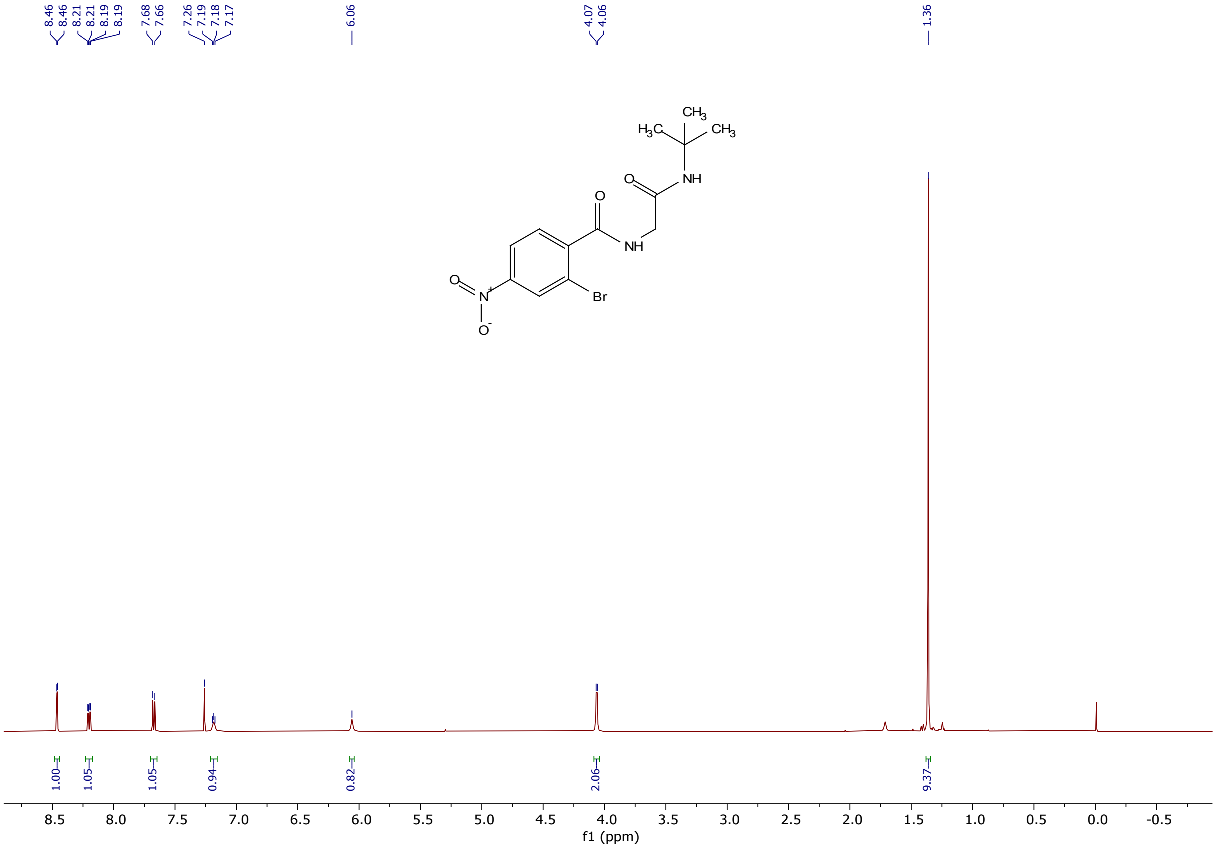

^{1}H NMR spectrum of **1j** (500 MHz, CDCl$_3$)

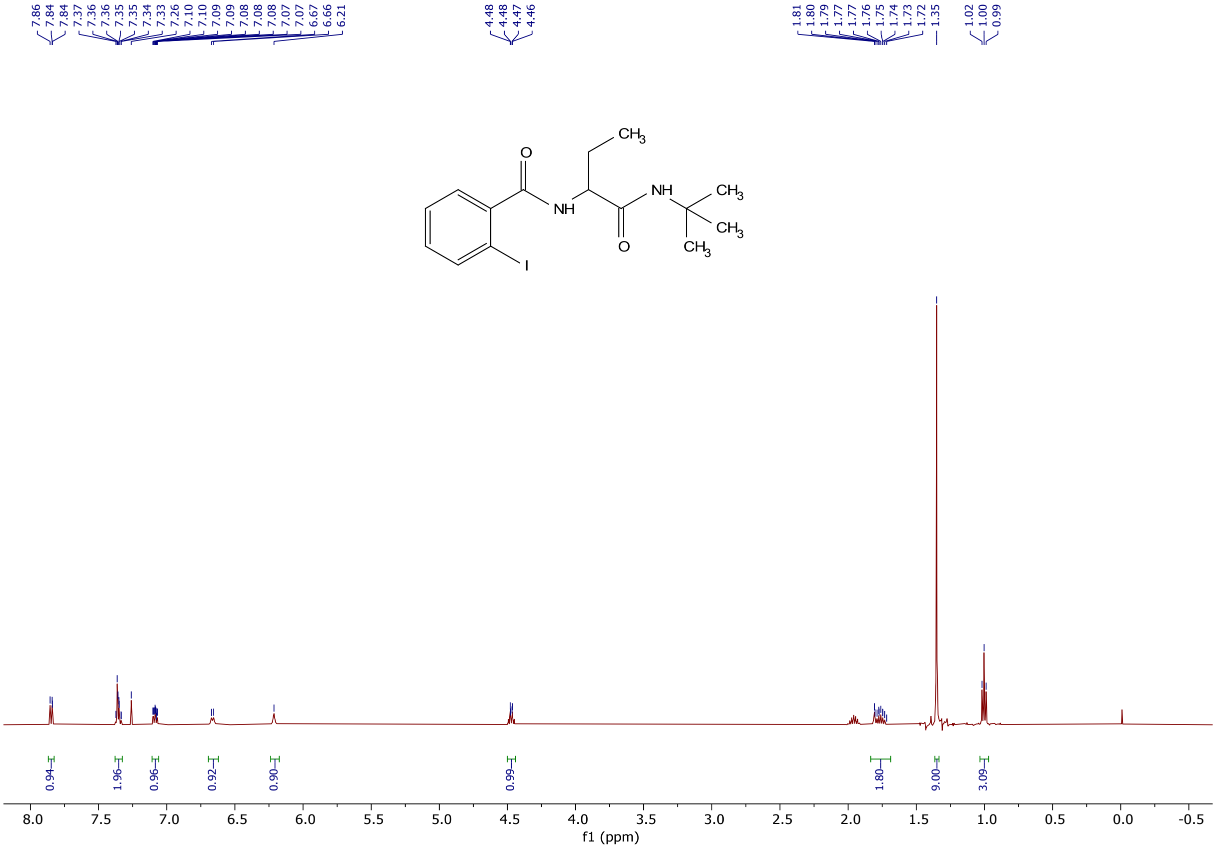

^{1}H NMR spectrum of **1b** (500 MHz, $CDCl_3$)

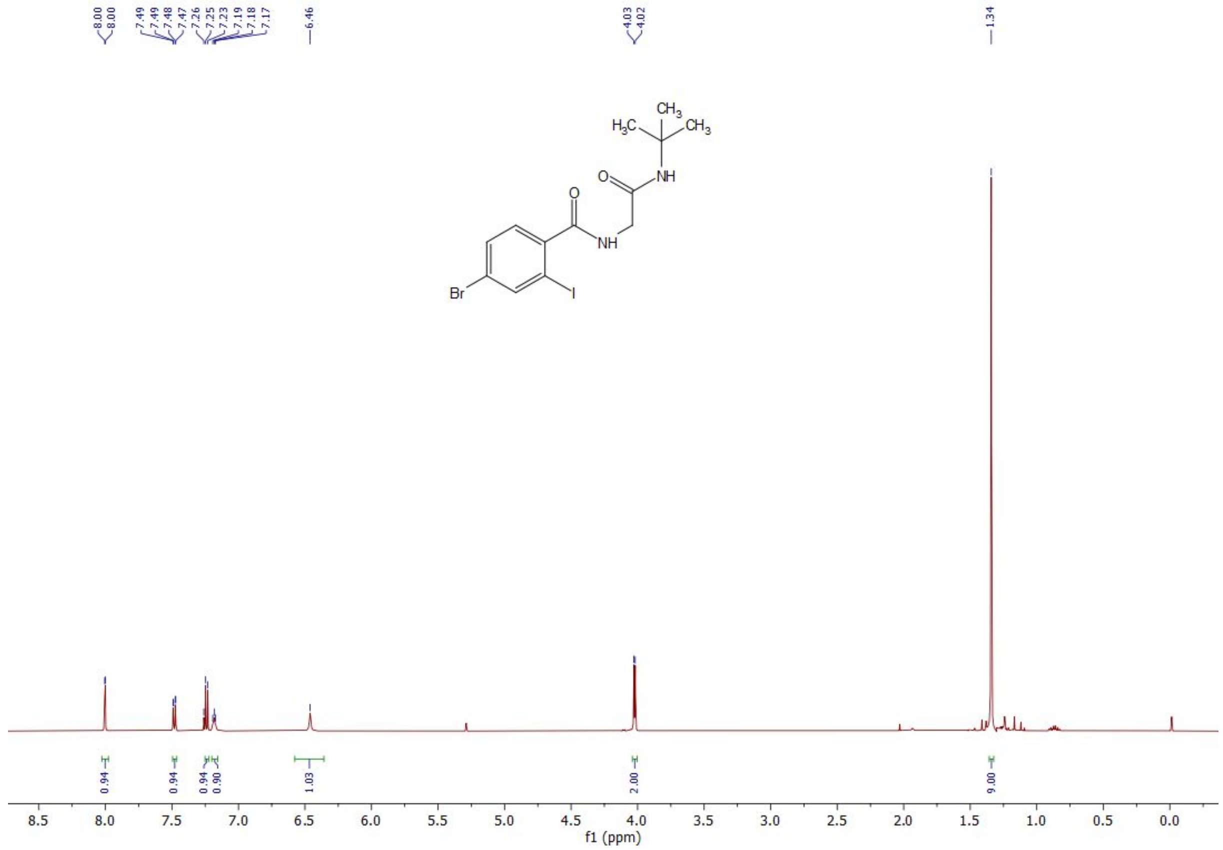

$^{13}C\{^{1}H\}$ NMR spectrum of **1b** (126 MHz, $CDCl_3$)

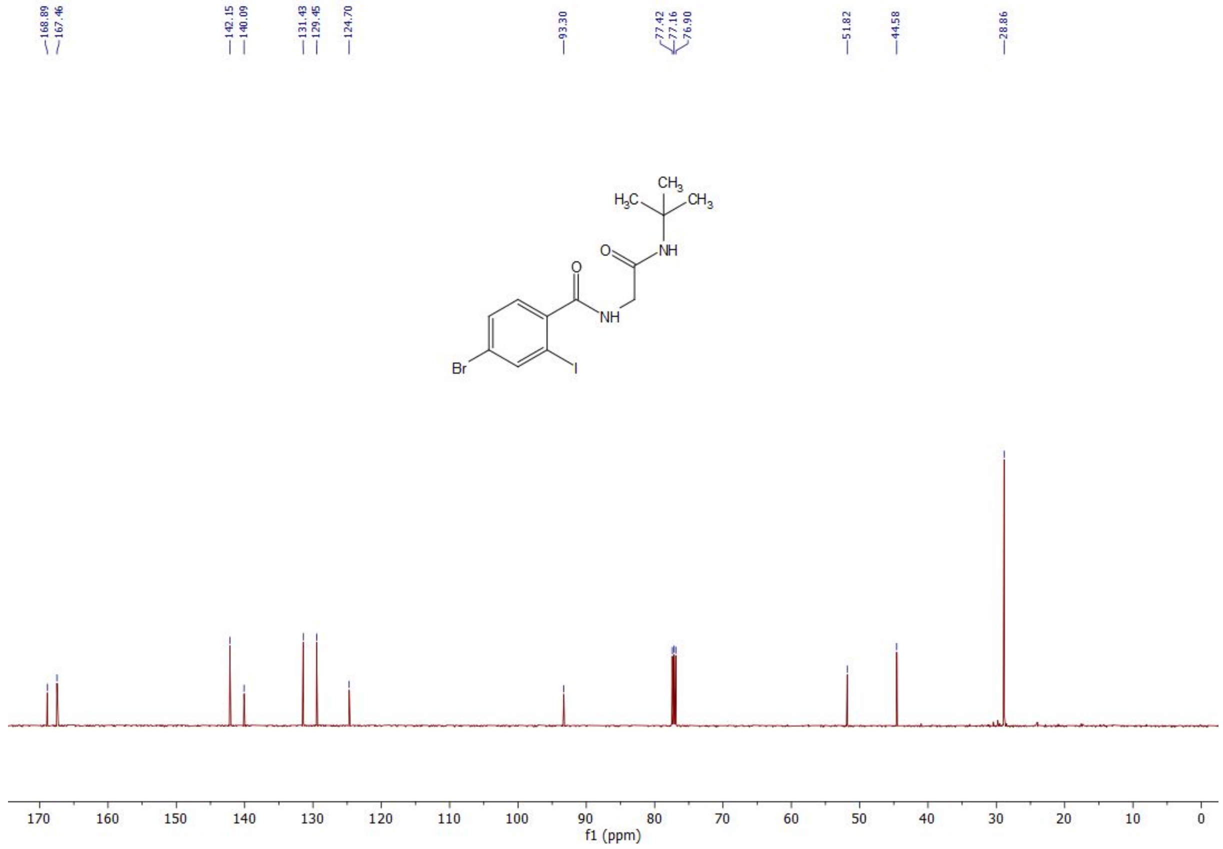

^{1}H NMR spectrum of **1c** (500 MHz, $CDCl_3$)

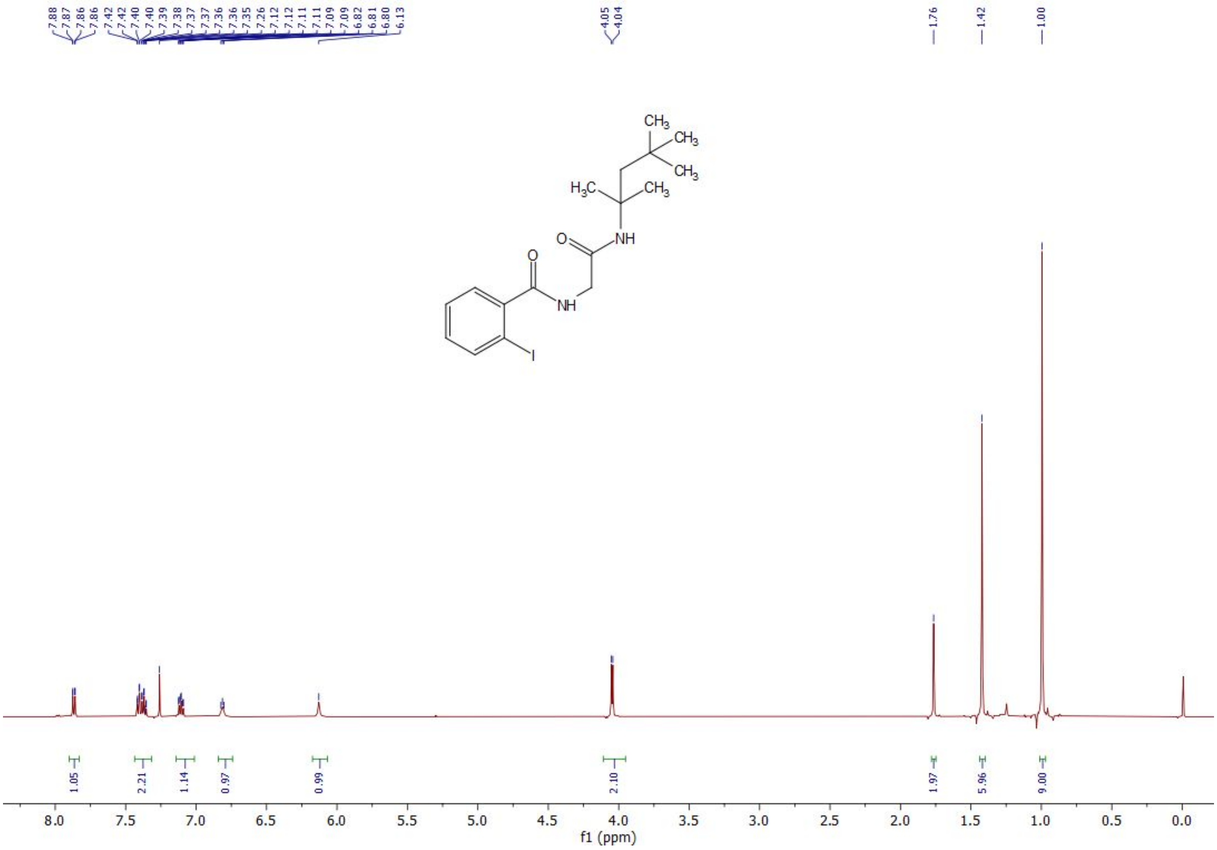

$^{13}C\{^{1}H\}$ NMR spectrum of **1c** (126 MHz, $CDCl_3$)

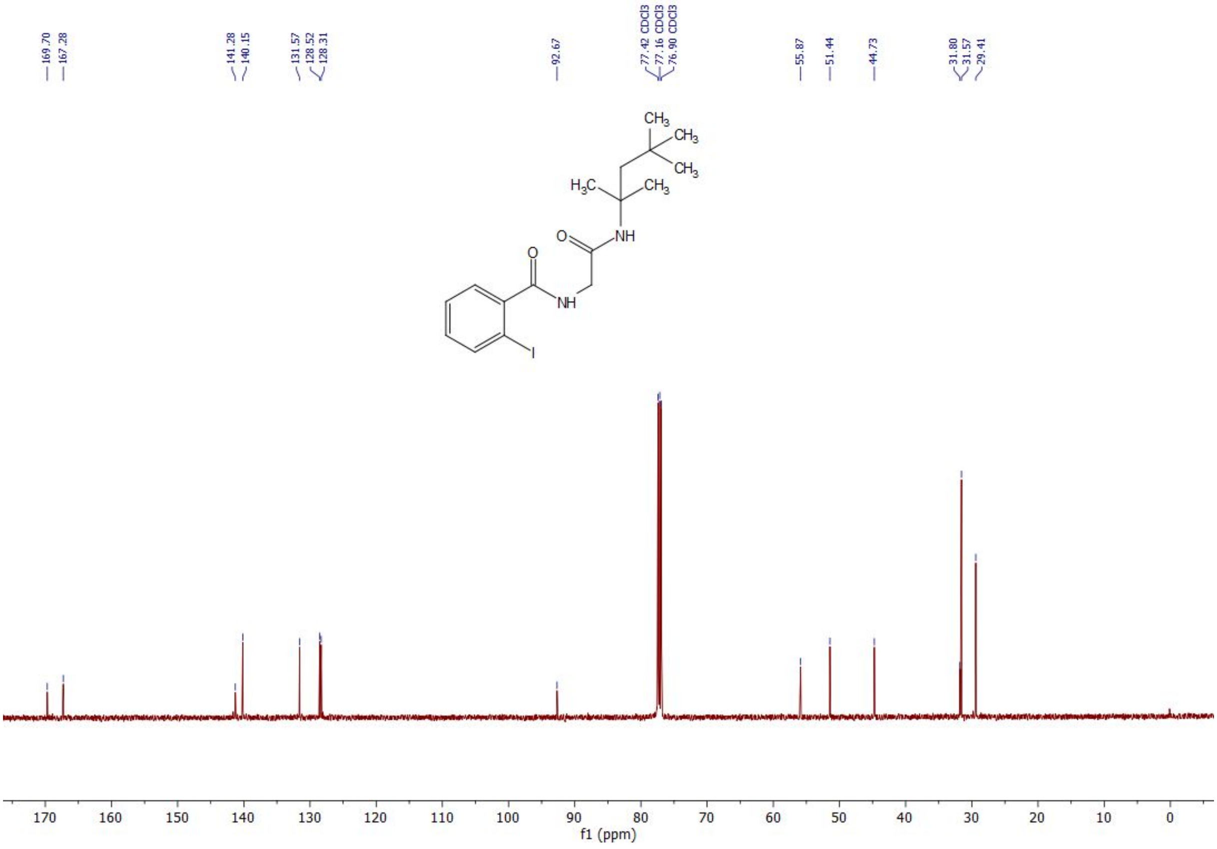

^{1}H NMR spectrum of **1e** (500 MHz, $CDCl_3$)

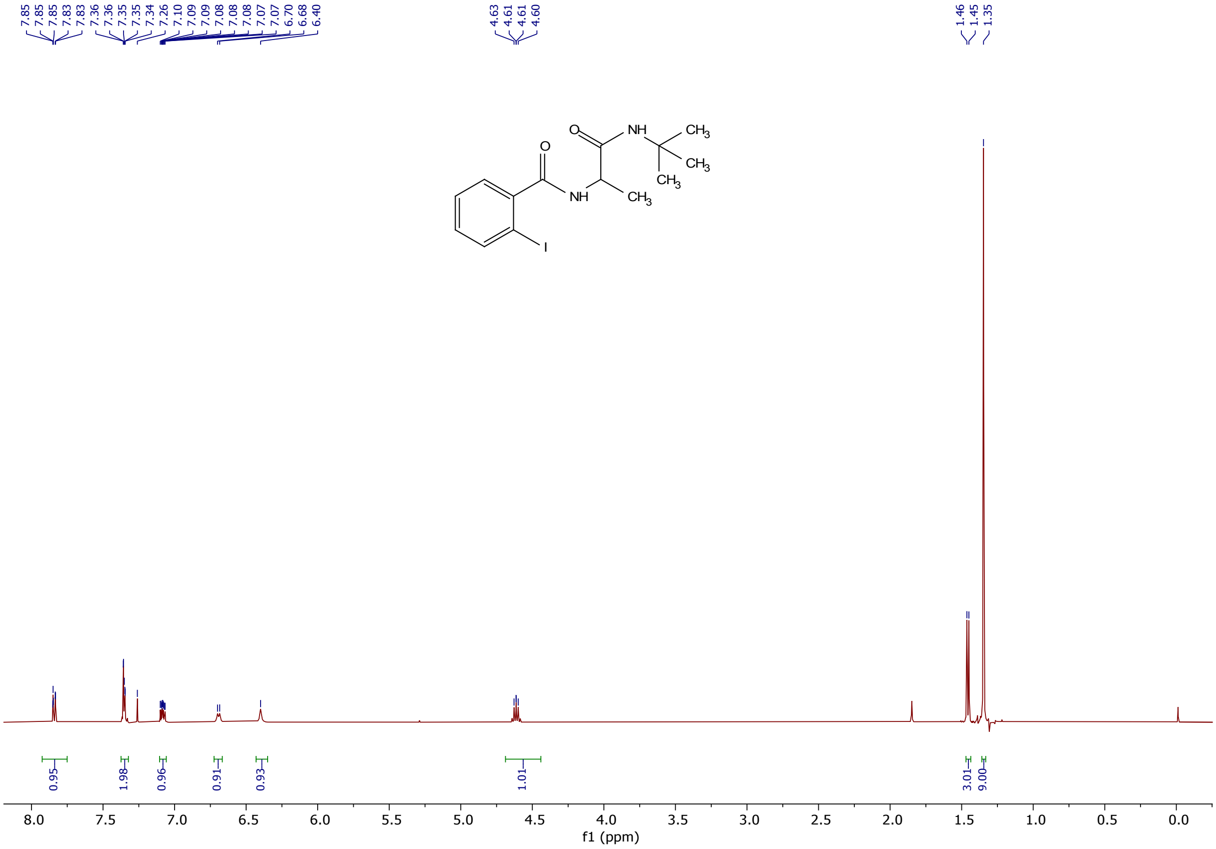

$^{13}C\{^1H\}$ NMR spectrum of **1e** (126 MHz, $CDCl_3$)

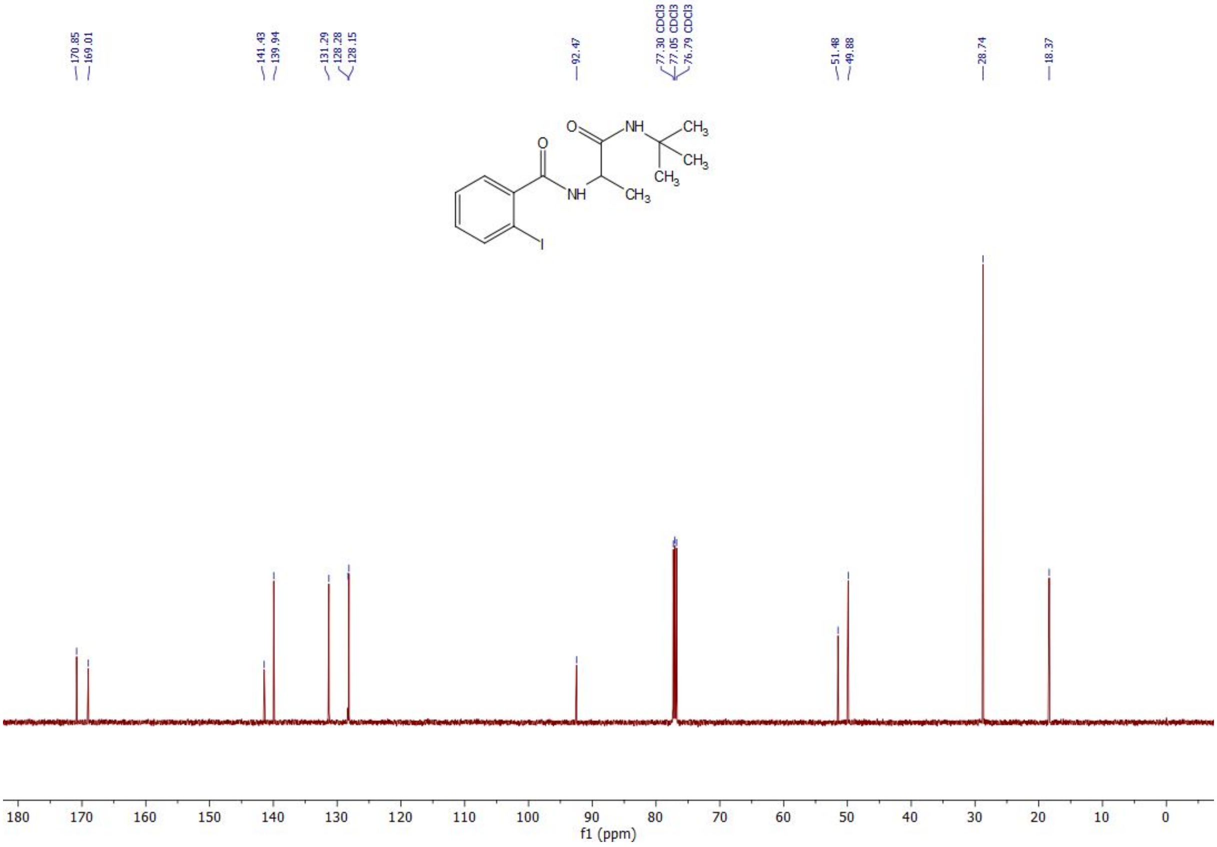

^{1}H NMR spectrum of **1g** (500 MHz, $CDCl_3$)

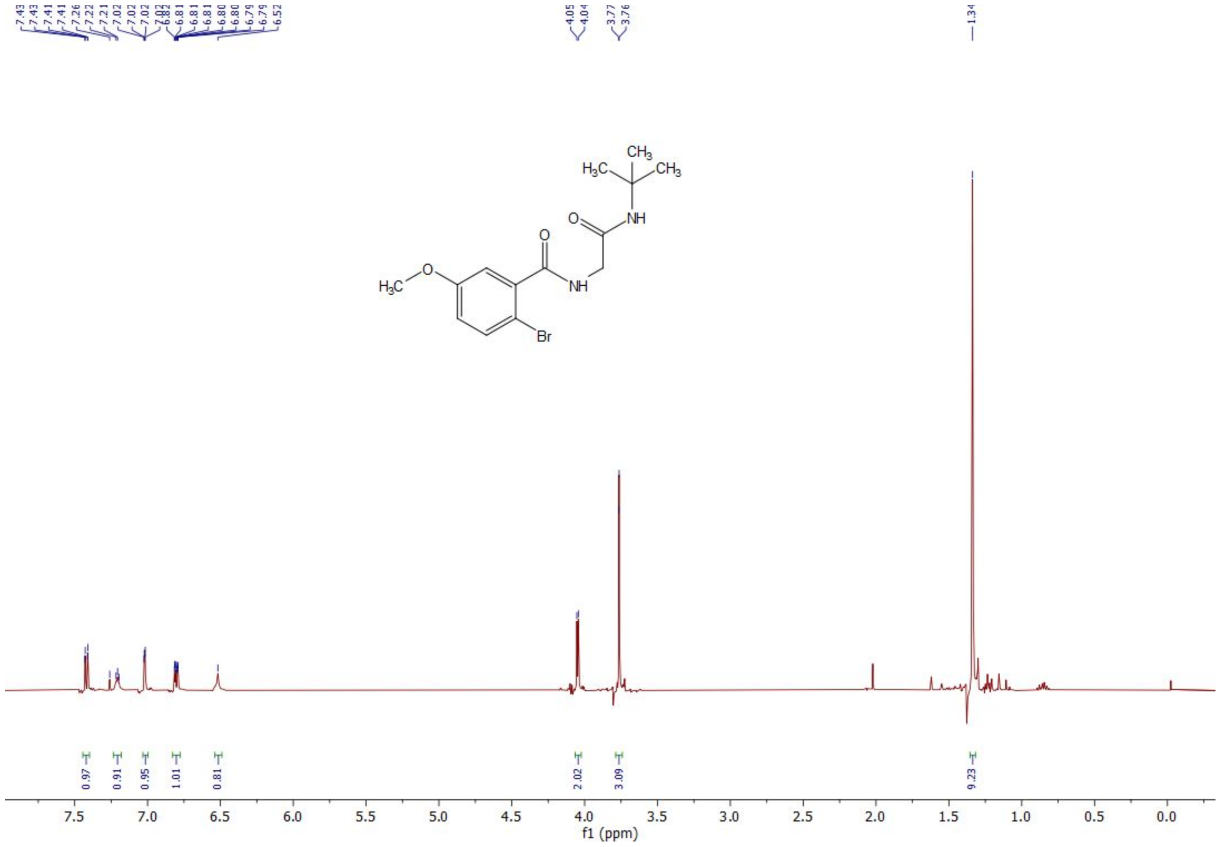

^{13}C{^{1}H} NMR spectrum of **1g** (126 MHz, $CDCl_3$)

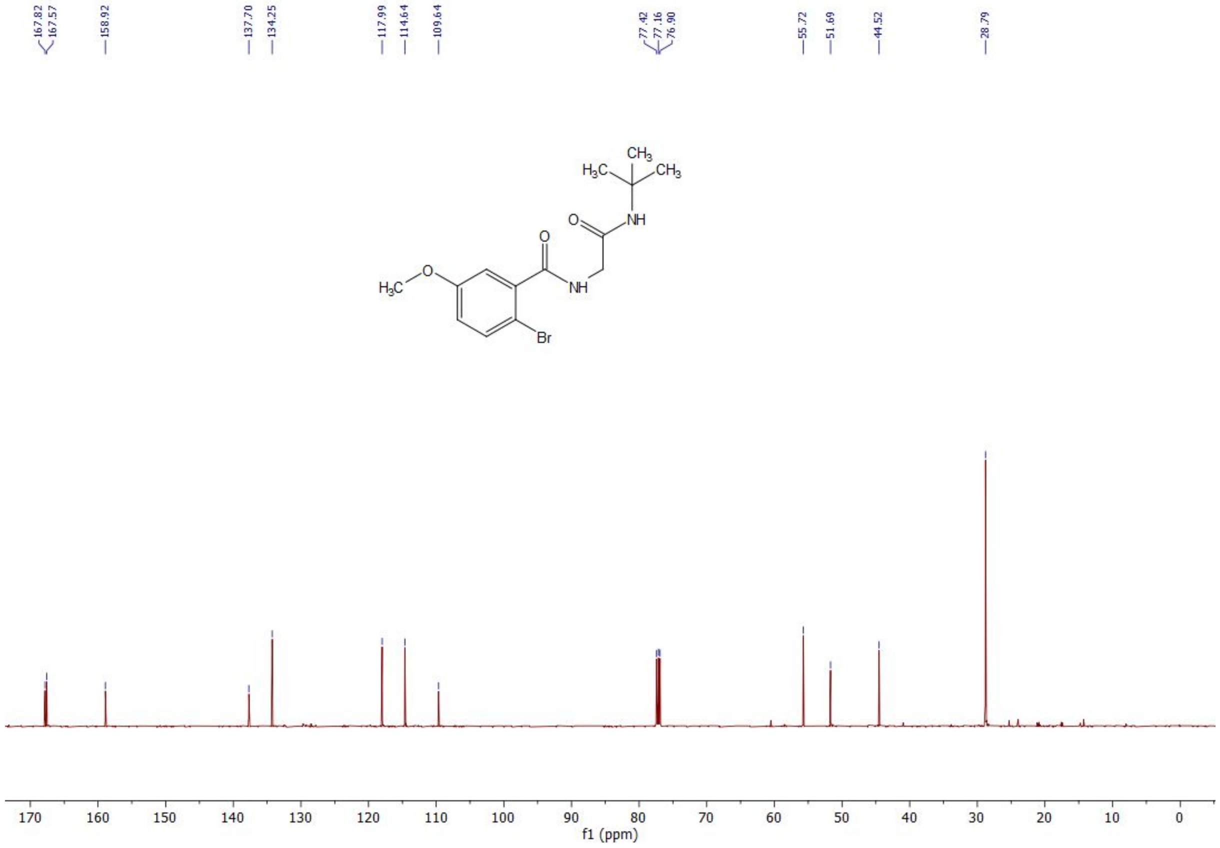

^{1}H NMR spectrum of **1h** (500 MHz, $CDCl_3$)

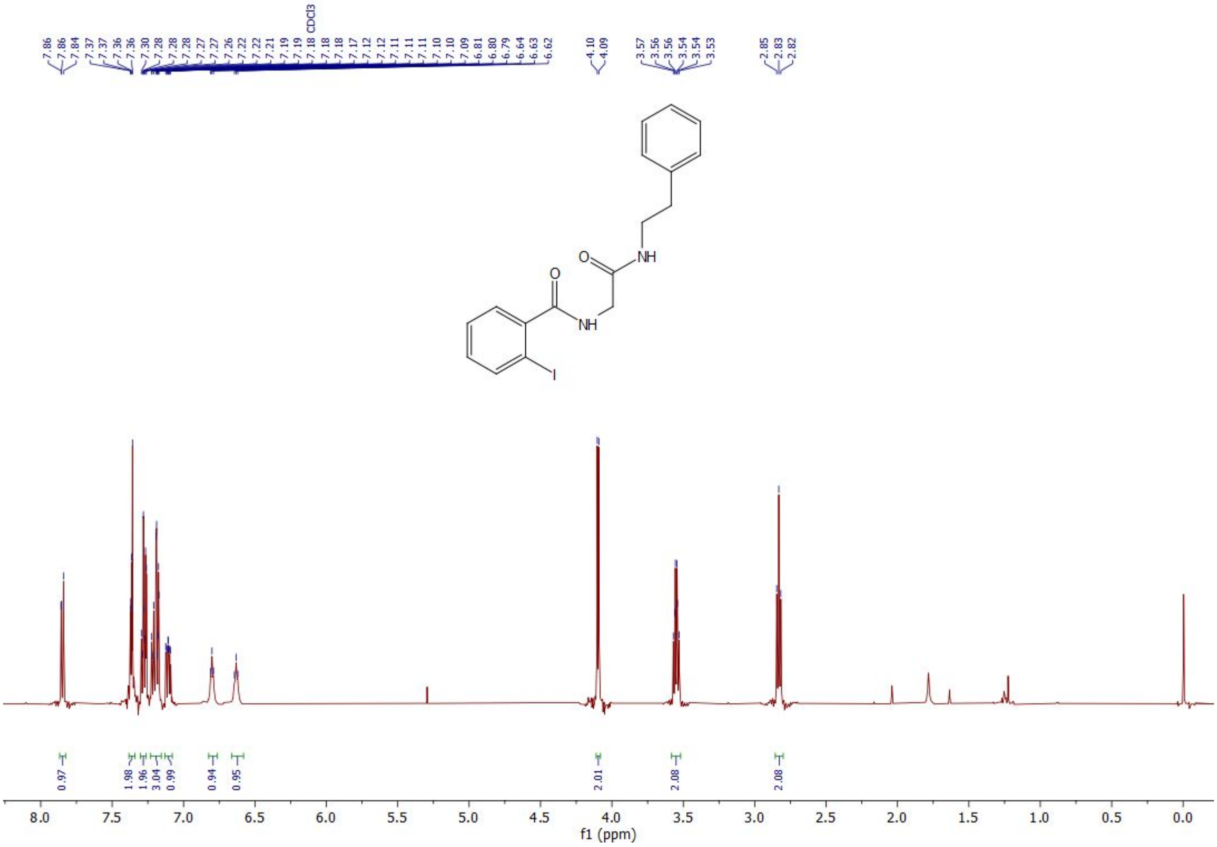

$^{13}C\{^{1}H\}$ NMR spectrum of **1h** (126 MHz, $CDCl_3$)

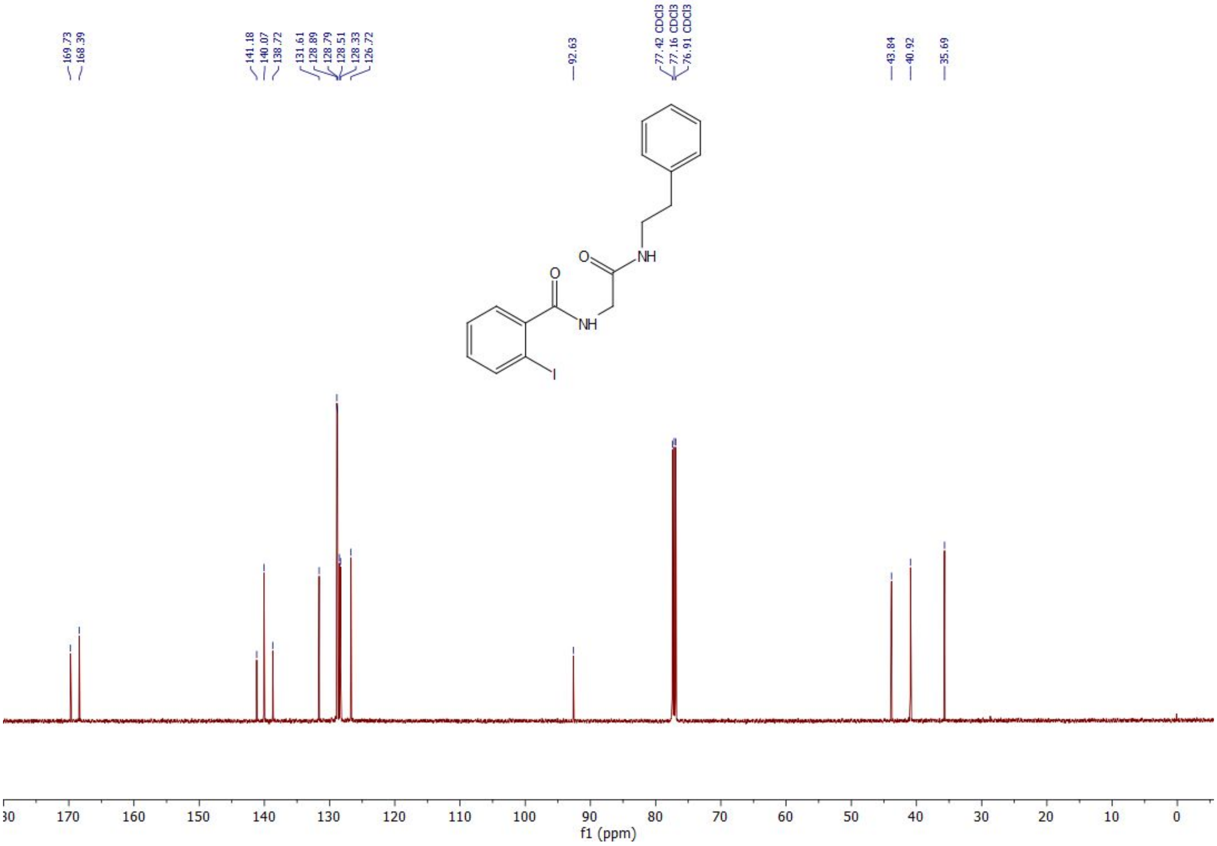

^{1}H NMR spectrum of **1i** (500 MHz, $CDCl_3$)

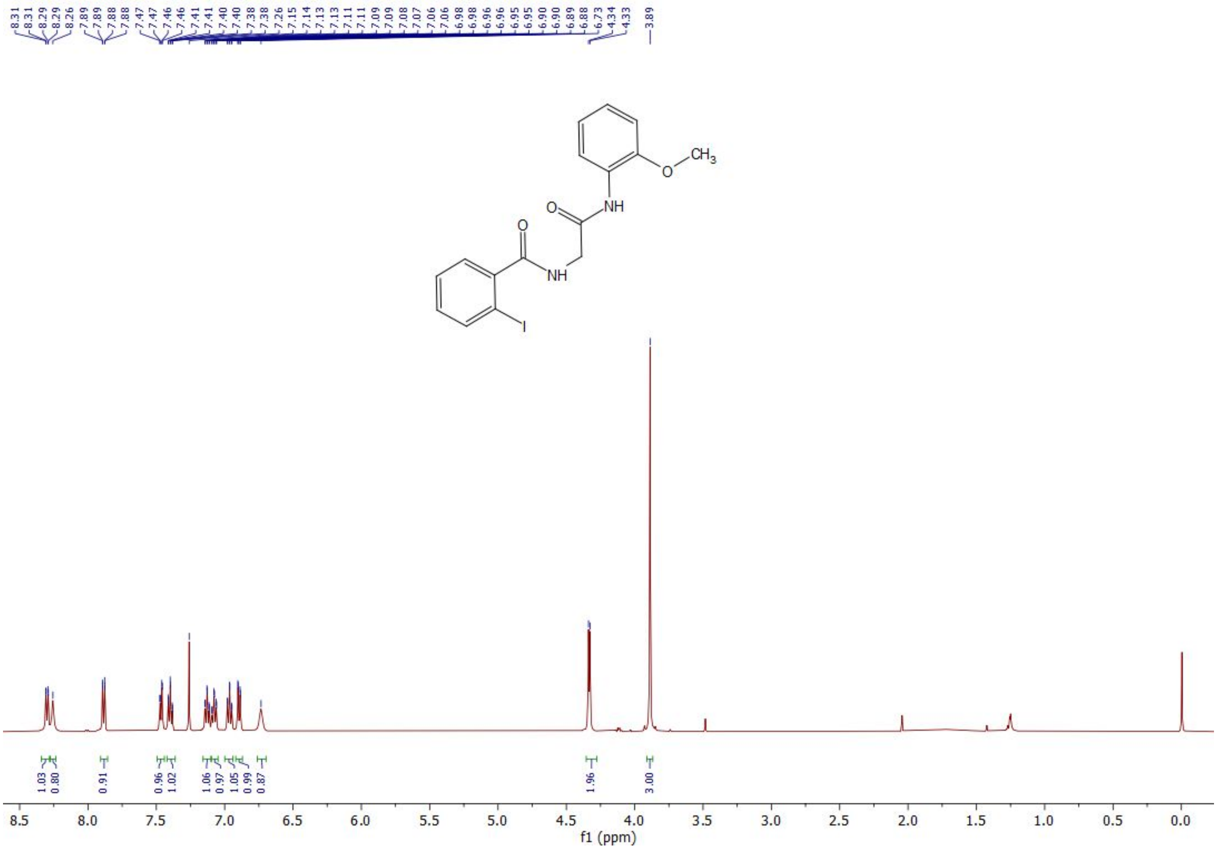

$^{13}C\{^{1}H\}$ NMR spectrum of **1i** (126 MHz, $CDCl_3$)

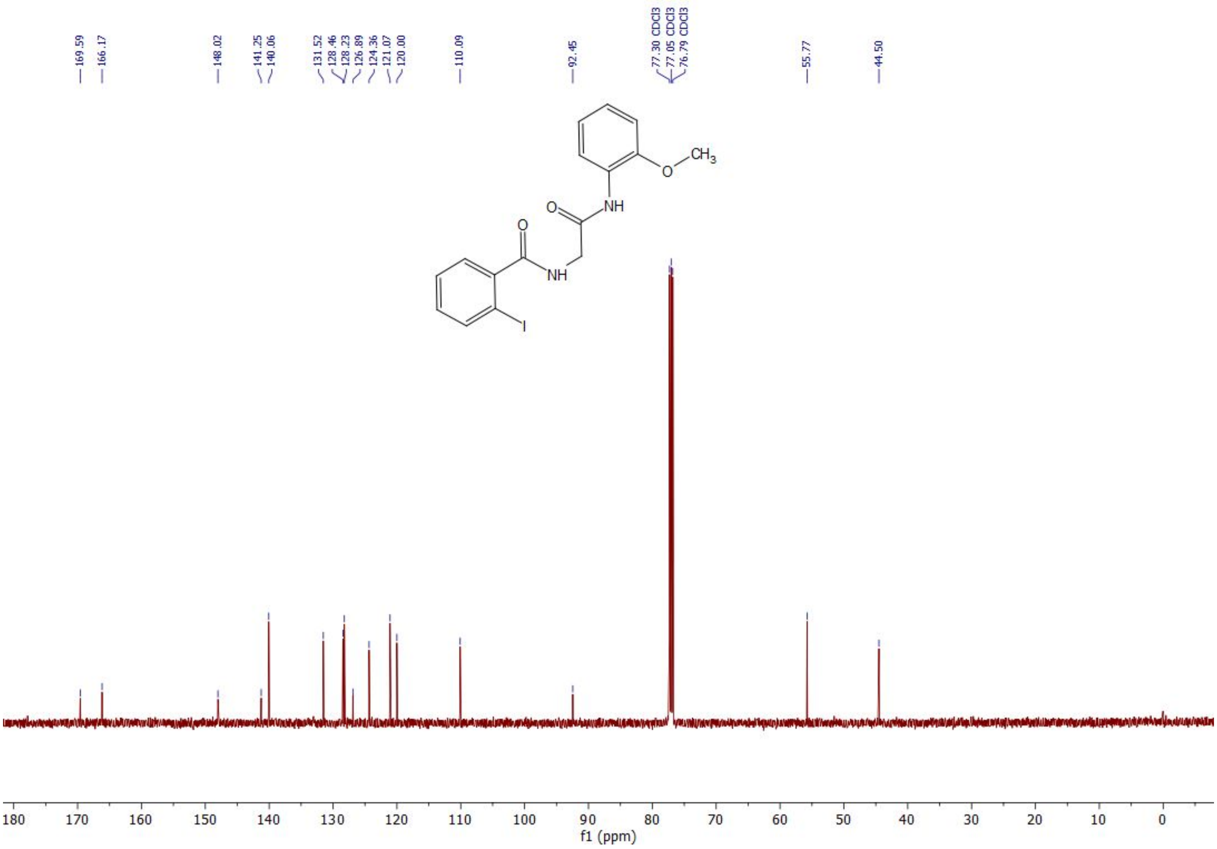

^{1}H NMR spectrum of **1k** (500 MHz, $CDCl_3$)

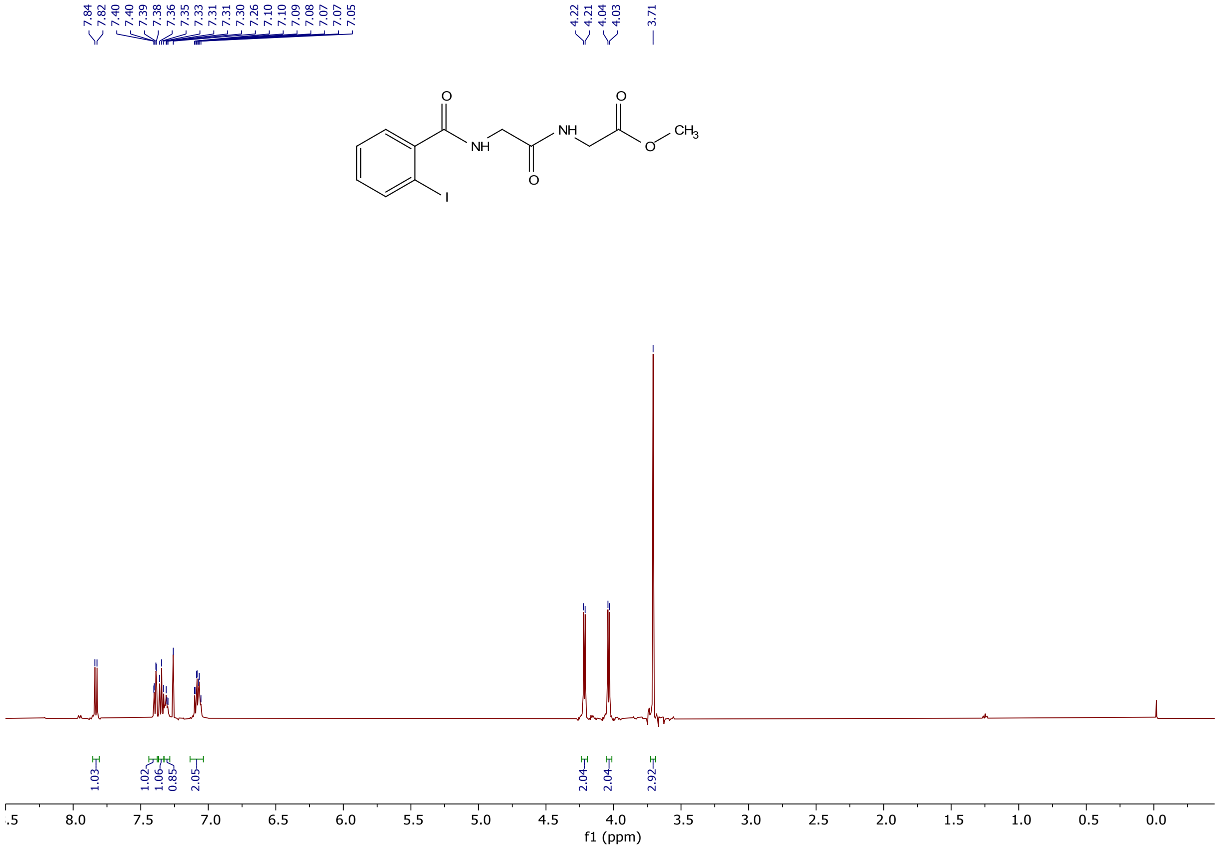

$^{13}C\{^{1}H\}$ NMR spectrum of **1k** (126 MHz, $CDCl_3$)

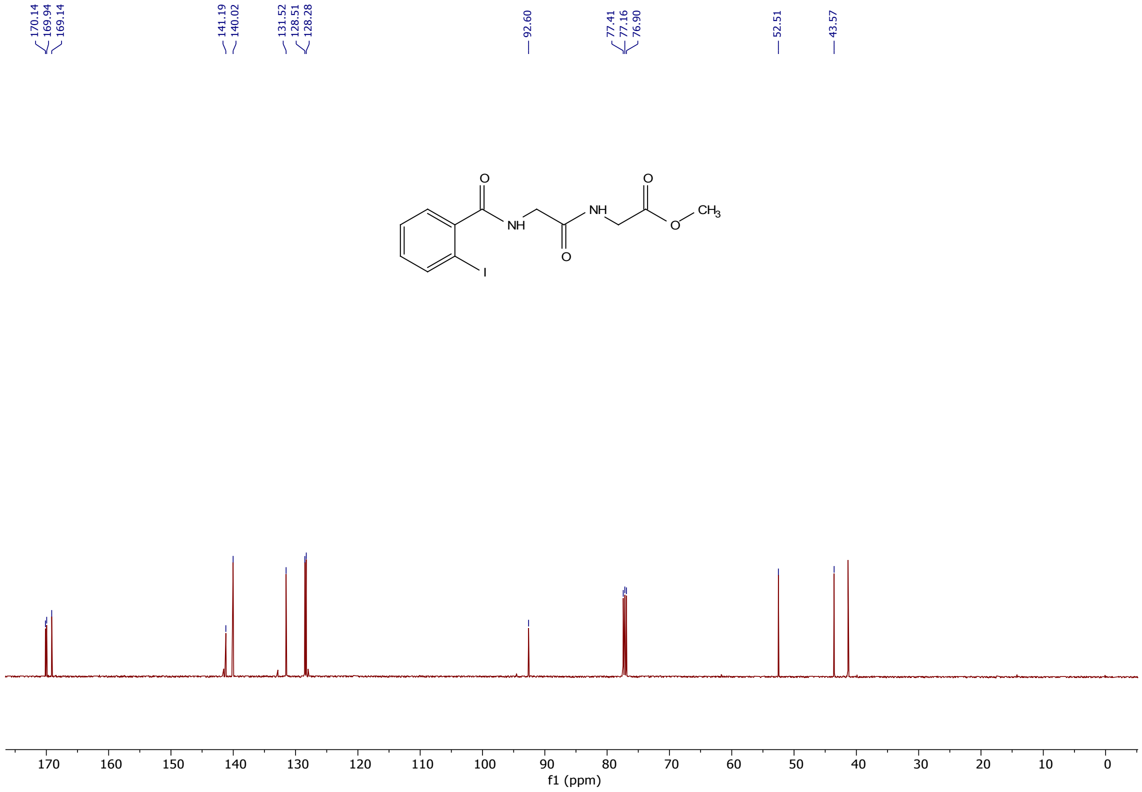

^{1}H NMR spectrum of **3a** (500 MHz, $CDCl_3$)

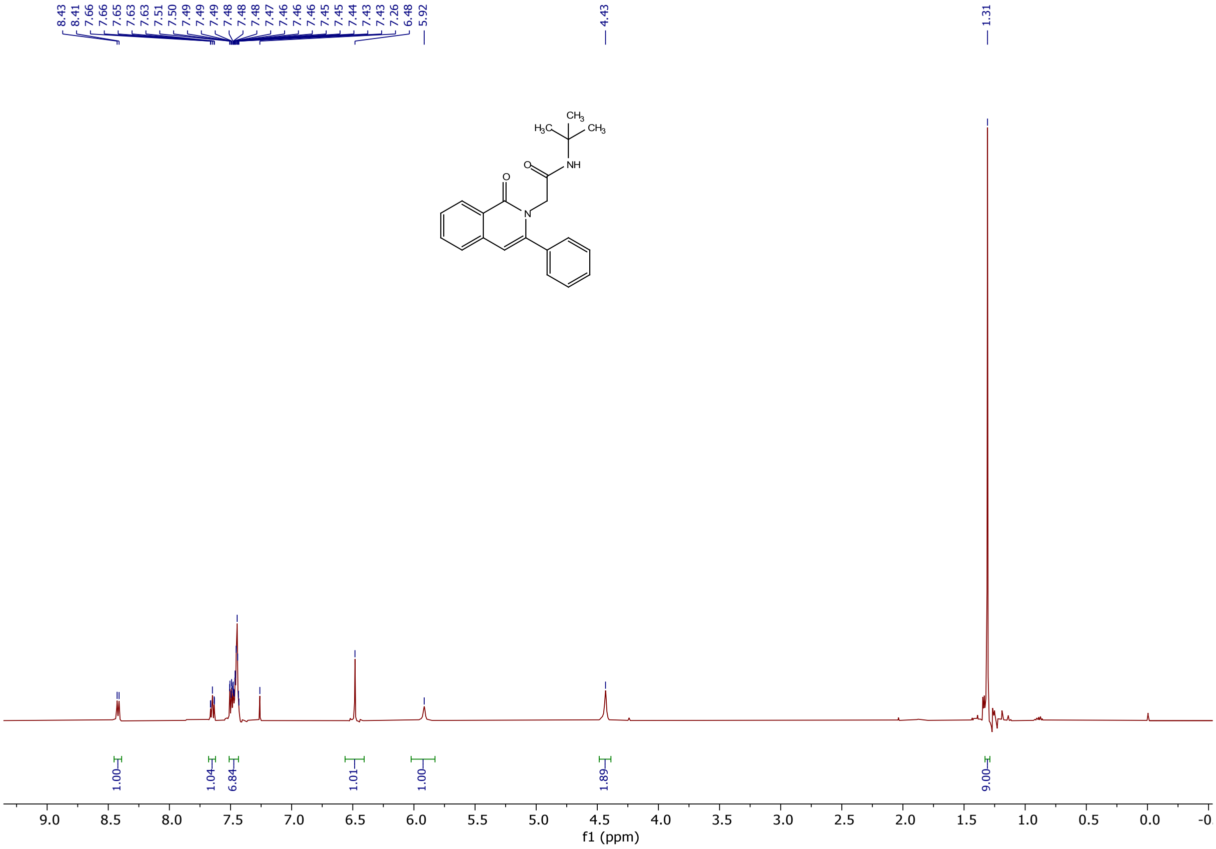

^{13}C{^{1}H} NMR spectrum of **3a** (126 MHz, $CDCl_3$)

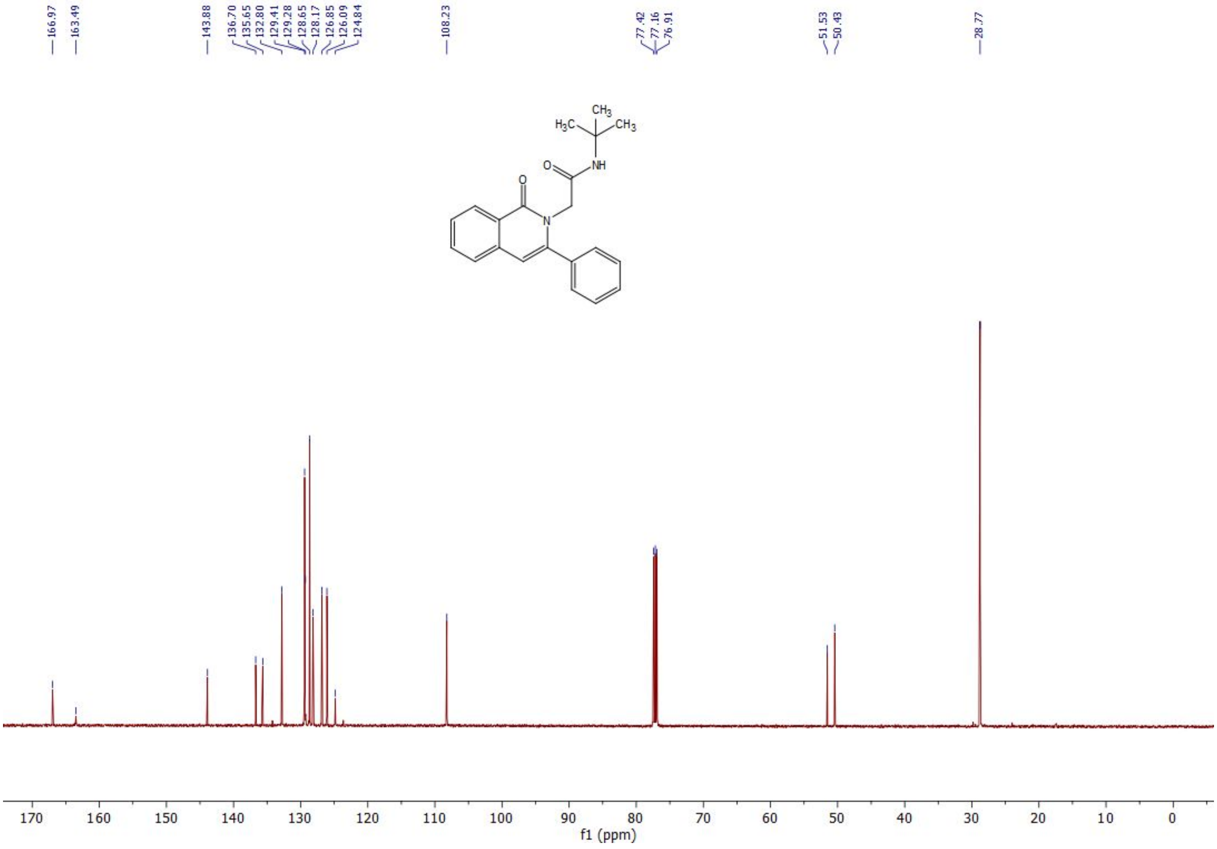

HMBC spectrum of **3a** (500 MHz, $CDCl_3$)

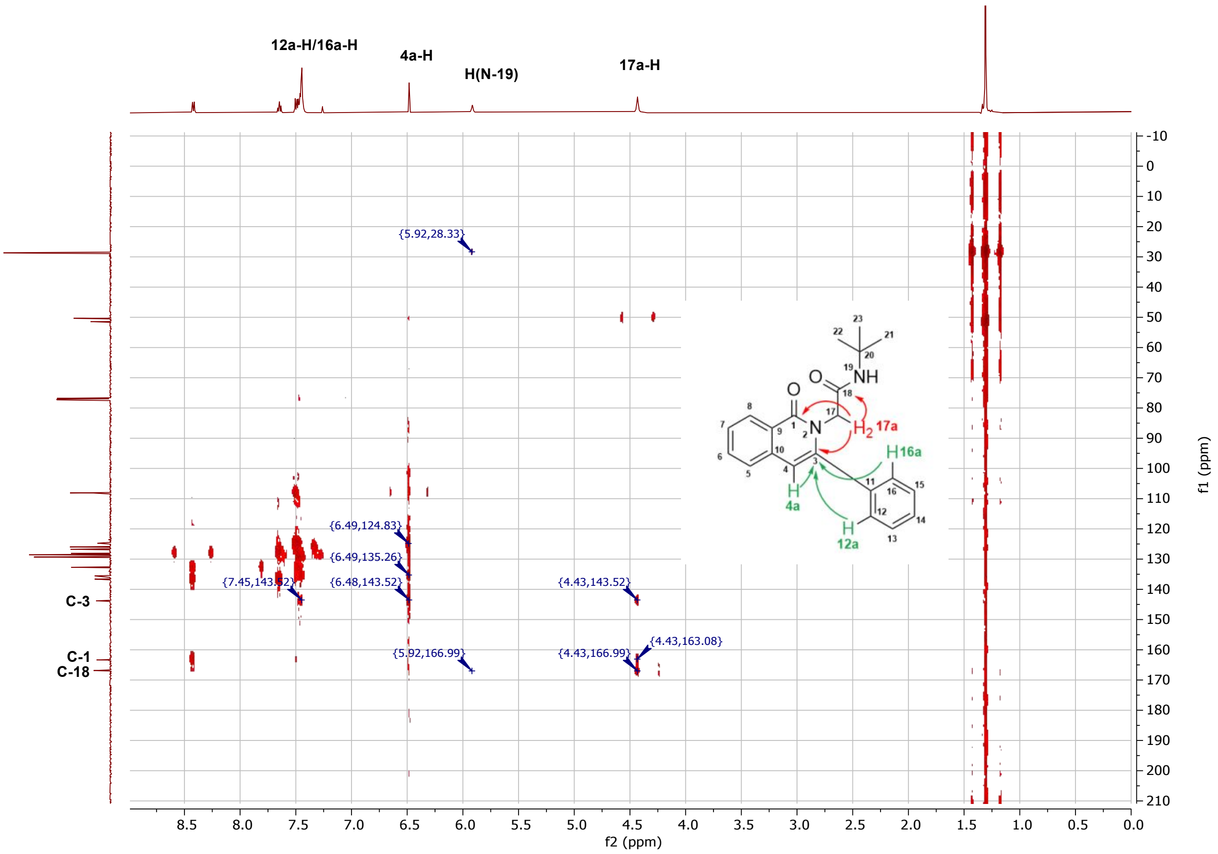

^{1}H NMR spectrum of **3b** (500 MHz, $CDCl_3$)

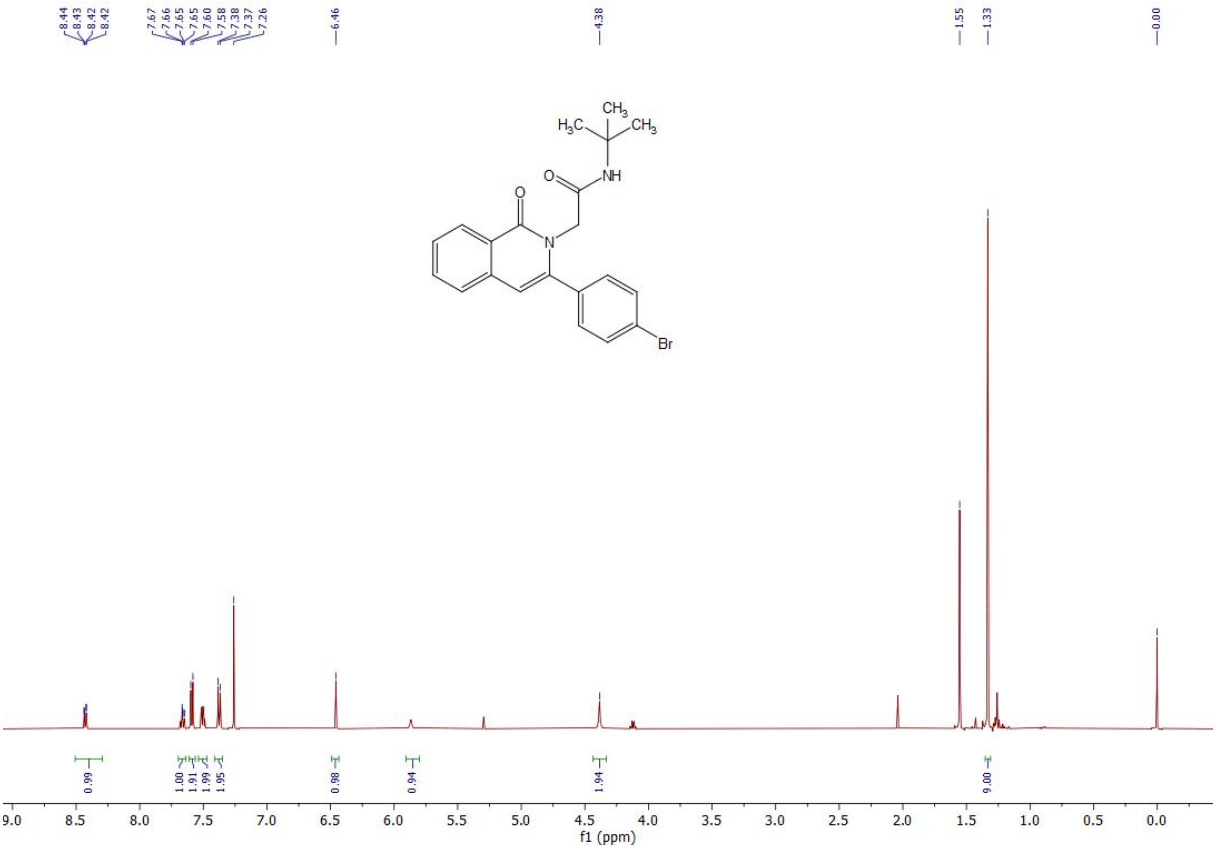

$^{13}C\{^{1}H\}$ NMR spectrum of **3b** (126 MHz, $CDCl_3$)

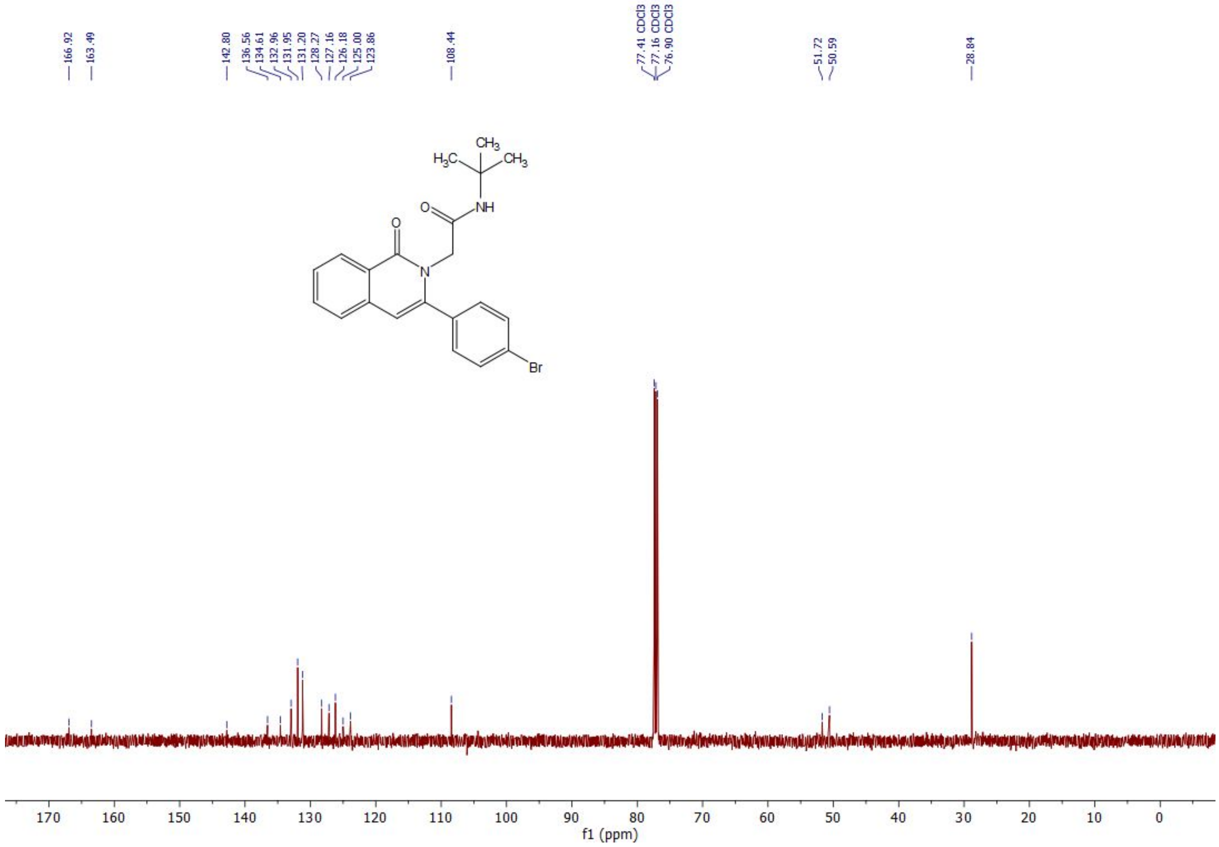

^{1}H NMR spectrum of **3c** (500 MHz, $CDCl_3$)

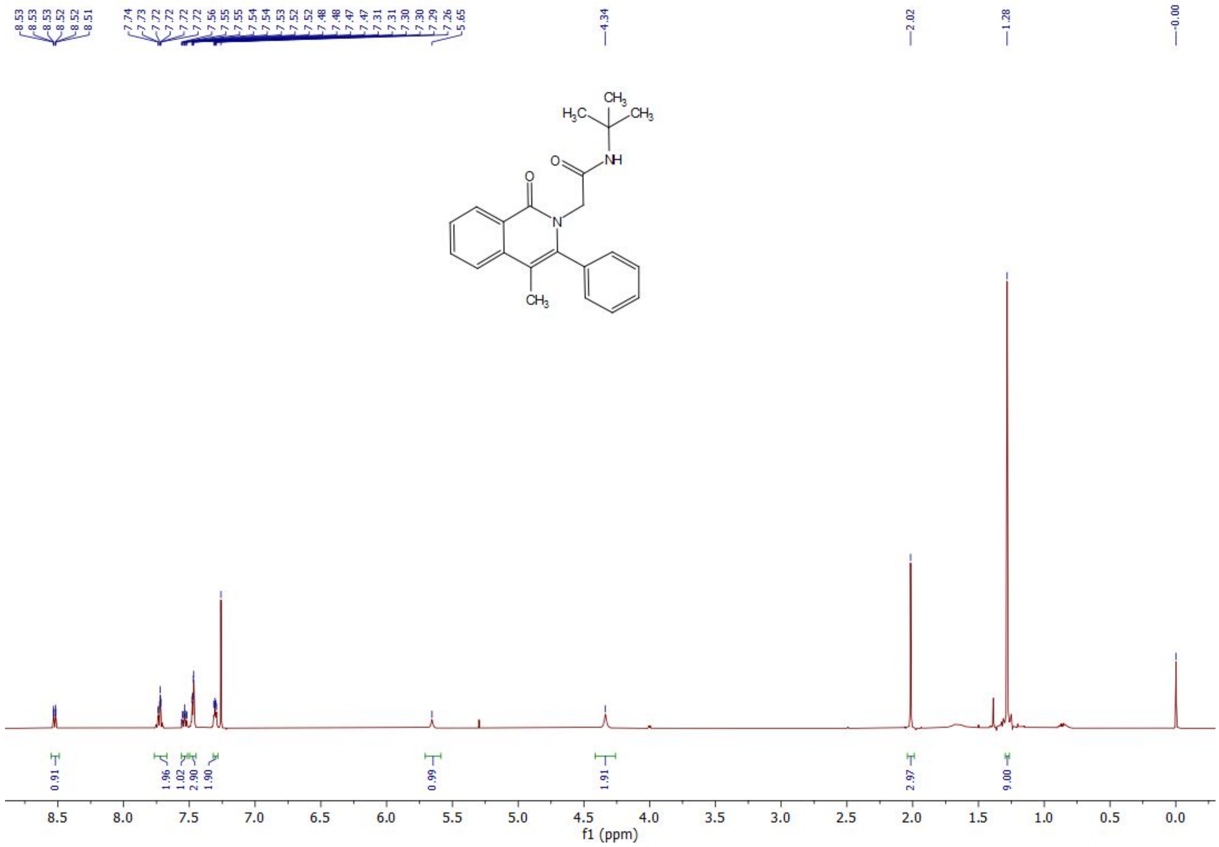

$^{13}C\{^{1}H\}$ NMR spectrum of **3c** (126 MHz, $CDCl_3$)

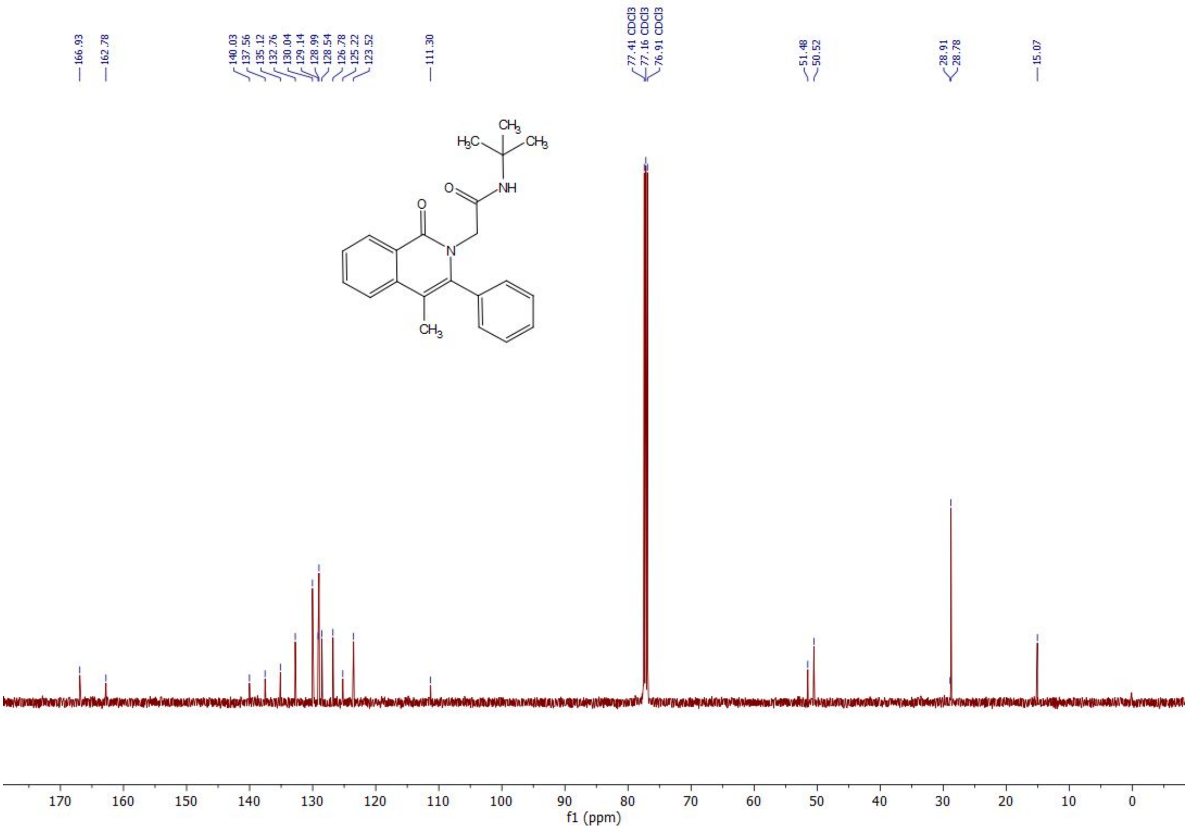

^{1}H NMR spectrum of **3d** (500 MHz, $CDCl_3$)

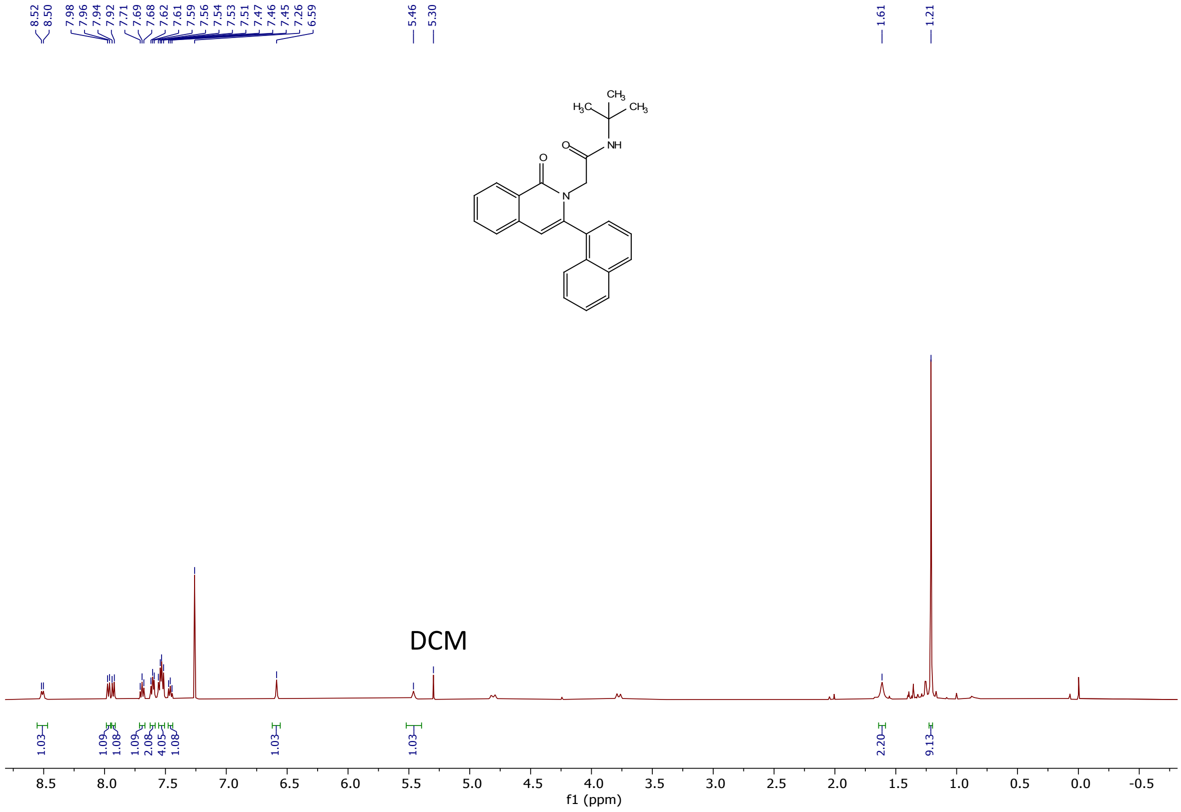

$^{13}C\{^{1}H\}$ NMR spectrum of **3d** (126 MHz, $CDCl_3$)

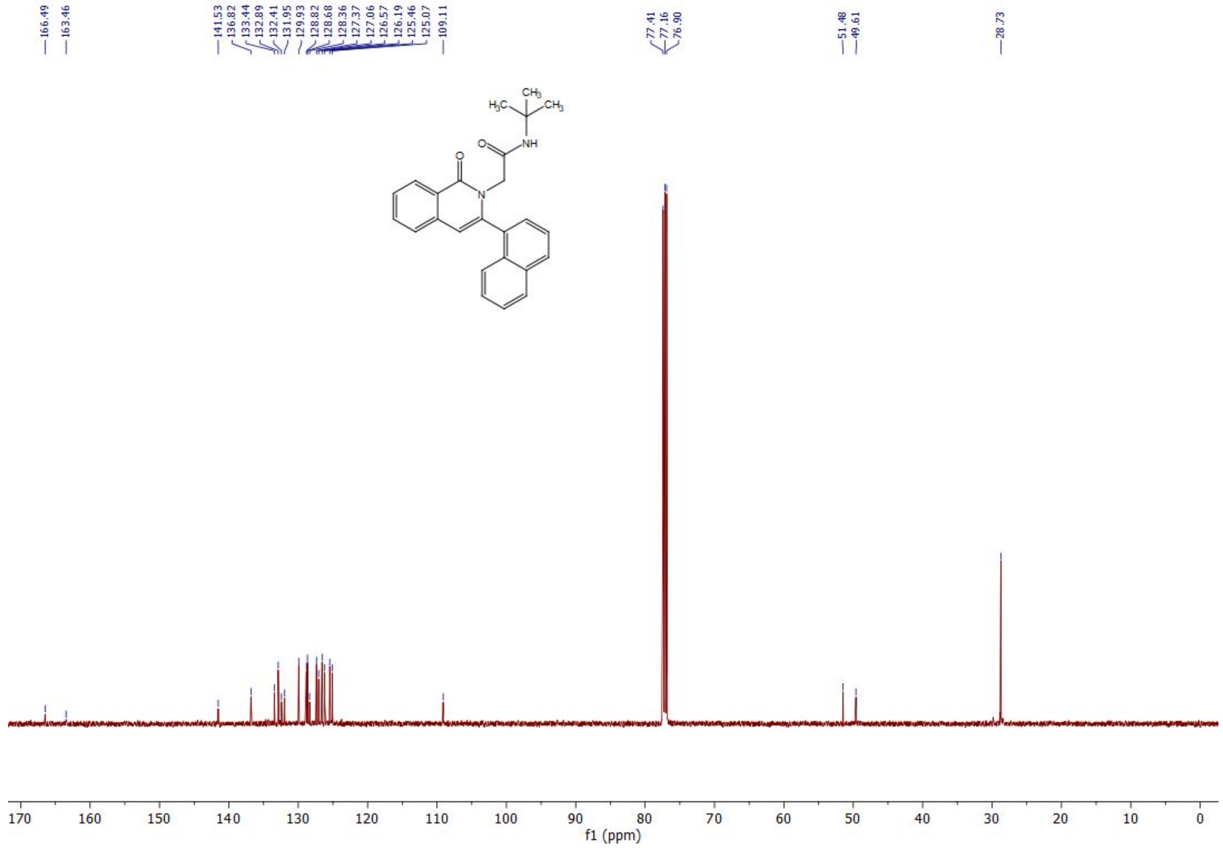

^{1}H NMR spectrum of **3e** (500 MHz, $CDCl_3$)

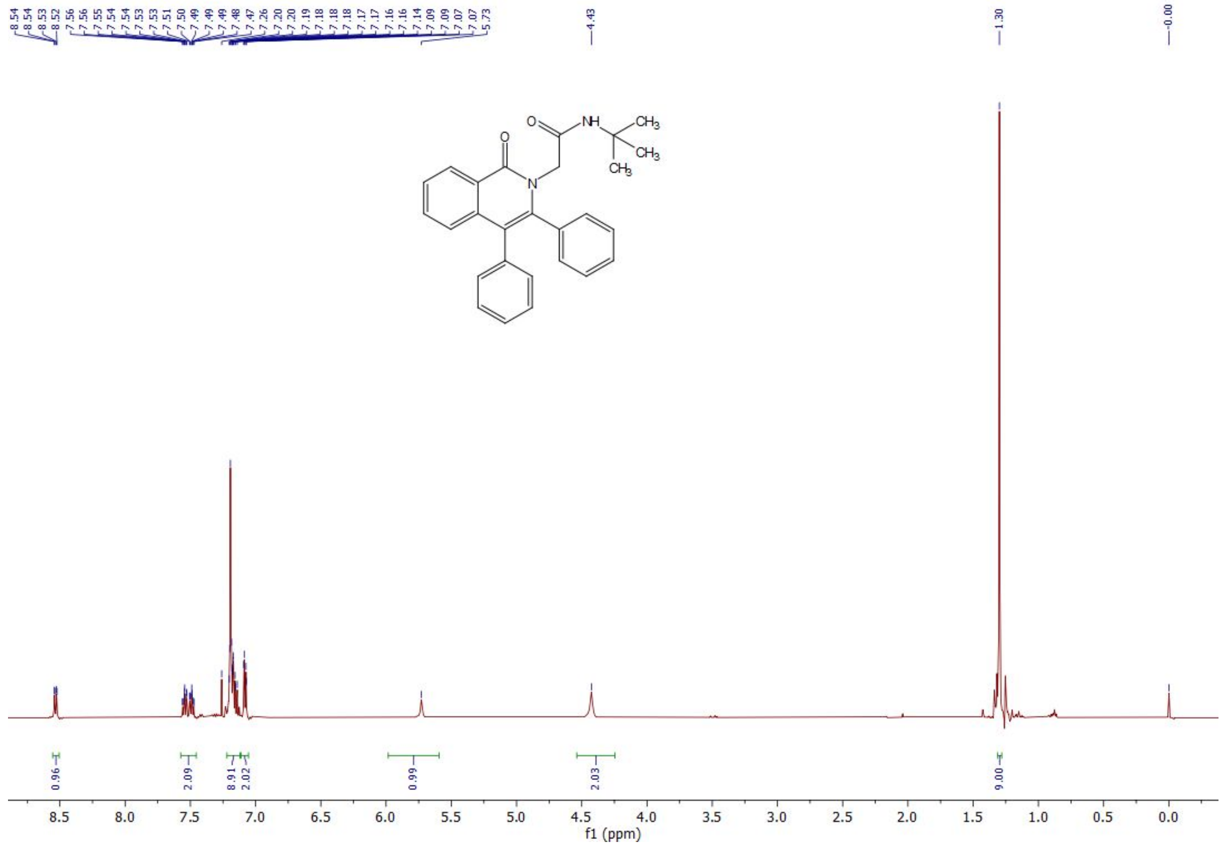

$^{13}C\{^1H\}$ NMR spectrum of **3e** (126 MHz, $CDCl_3$)

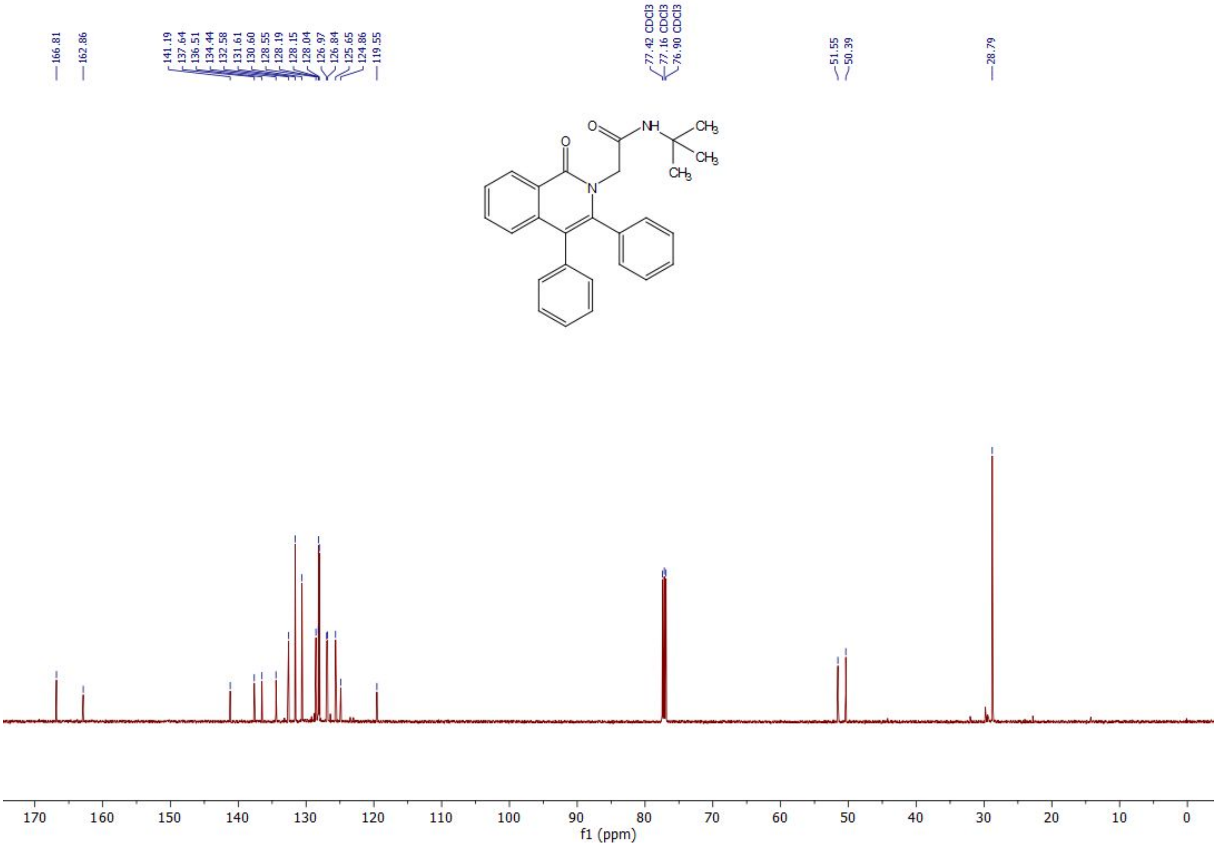

^1H NMR spectrum of **3f** (500 MHz, $CDCl_3$)

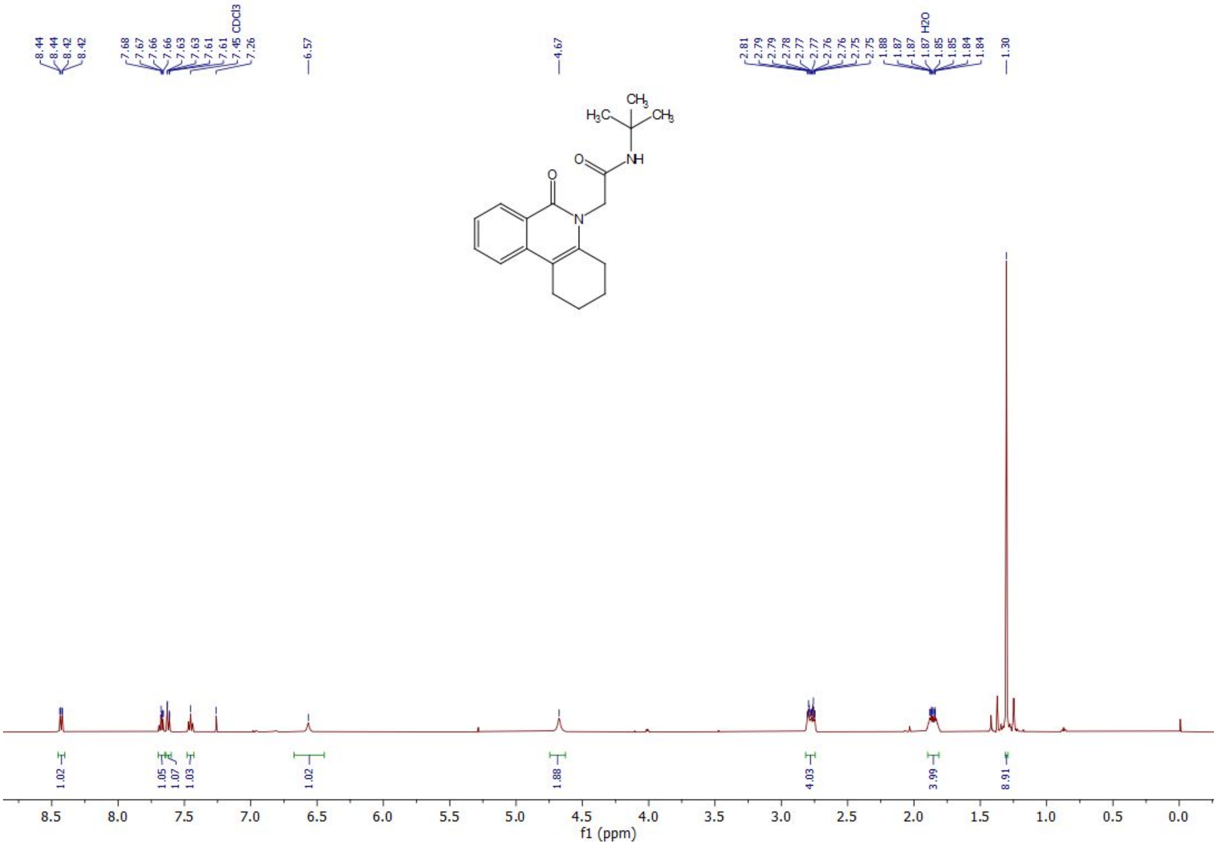

$^{13}C\{^{1}H\}$ NMR spectrum of **3f** (126 MHz, $CDCl_3$)

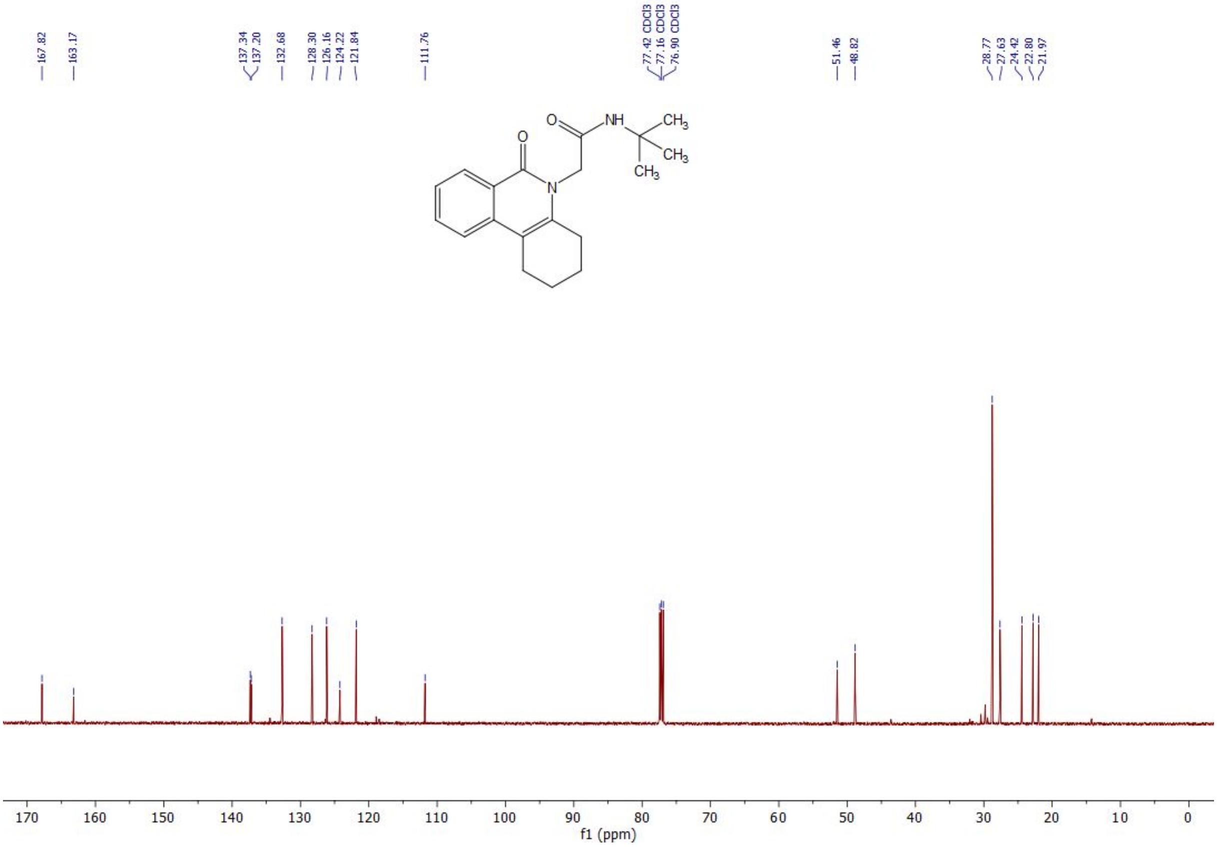

^{1}H NMR spectrum of **3g** (500 MHz, $CDCl_3$)

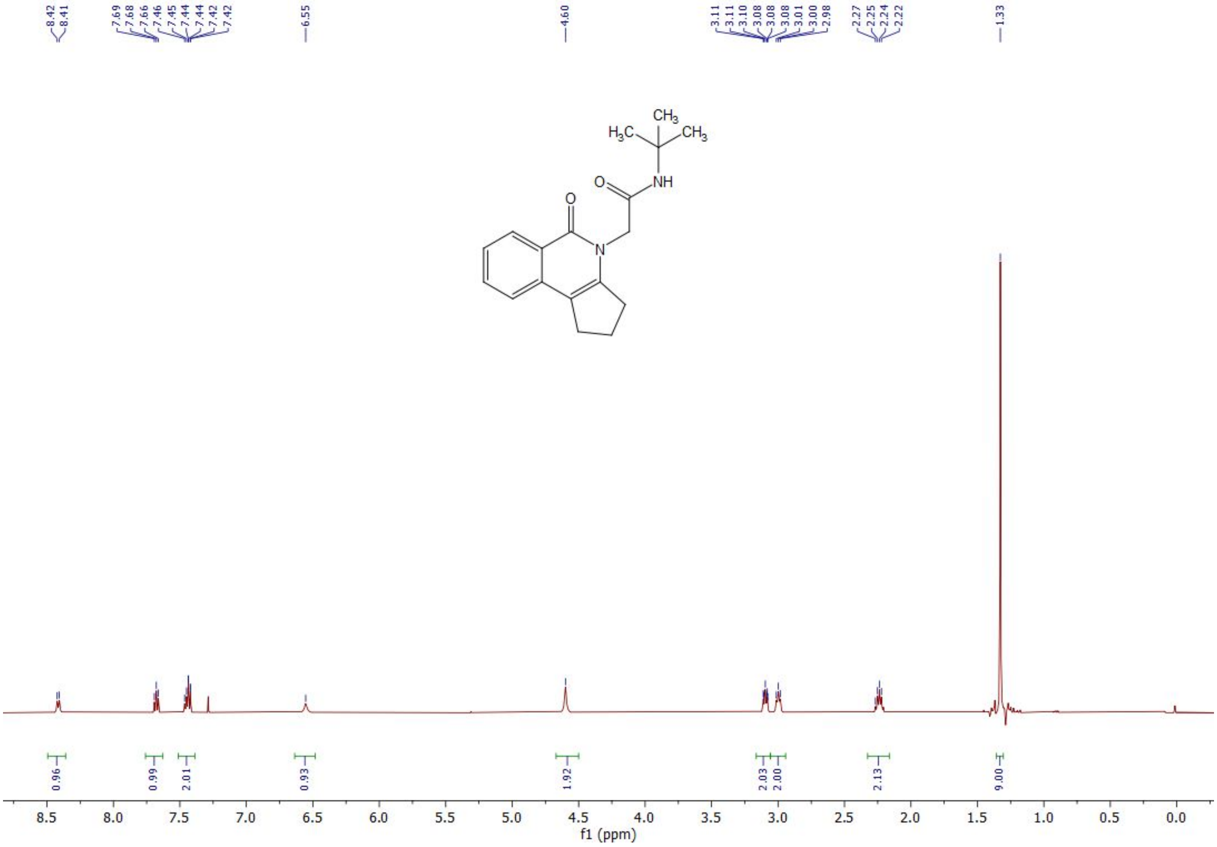

$^{13}C\{^{1}H\}$ NMR spectrum of **3g** (126 MHz, $CDCl_3$)

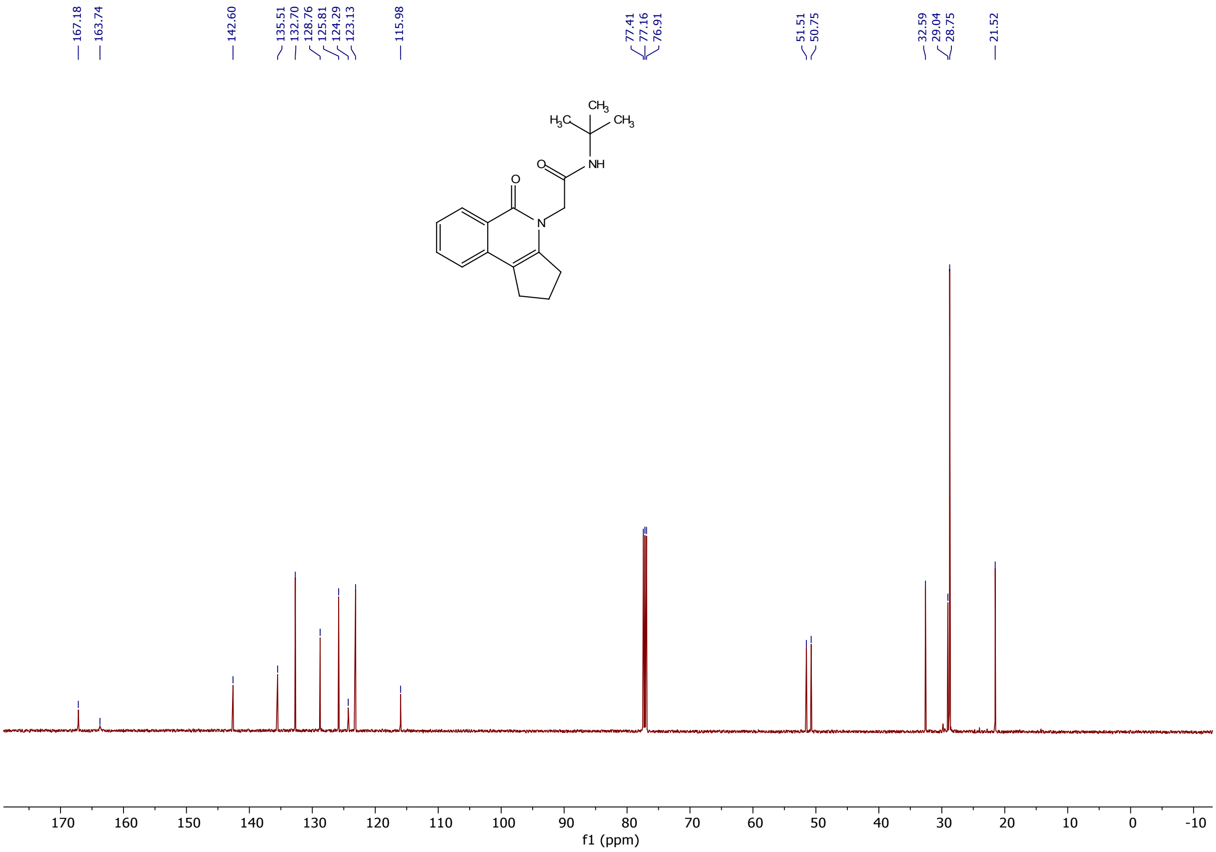

^{1}H NMR spectrum of **3h** (500 MHz, $CDCl_3$)

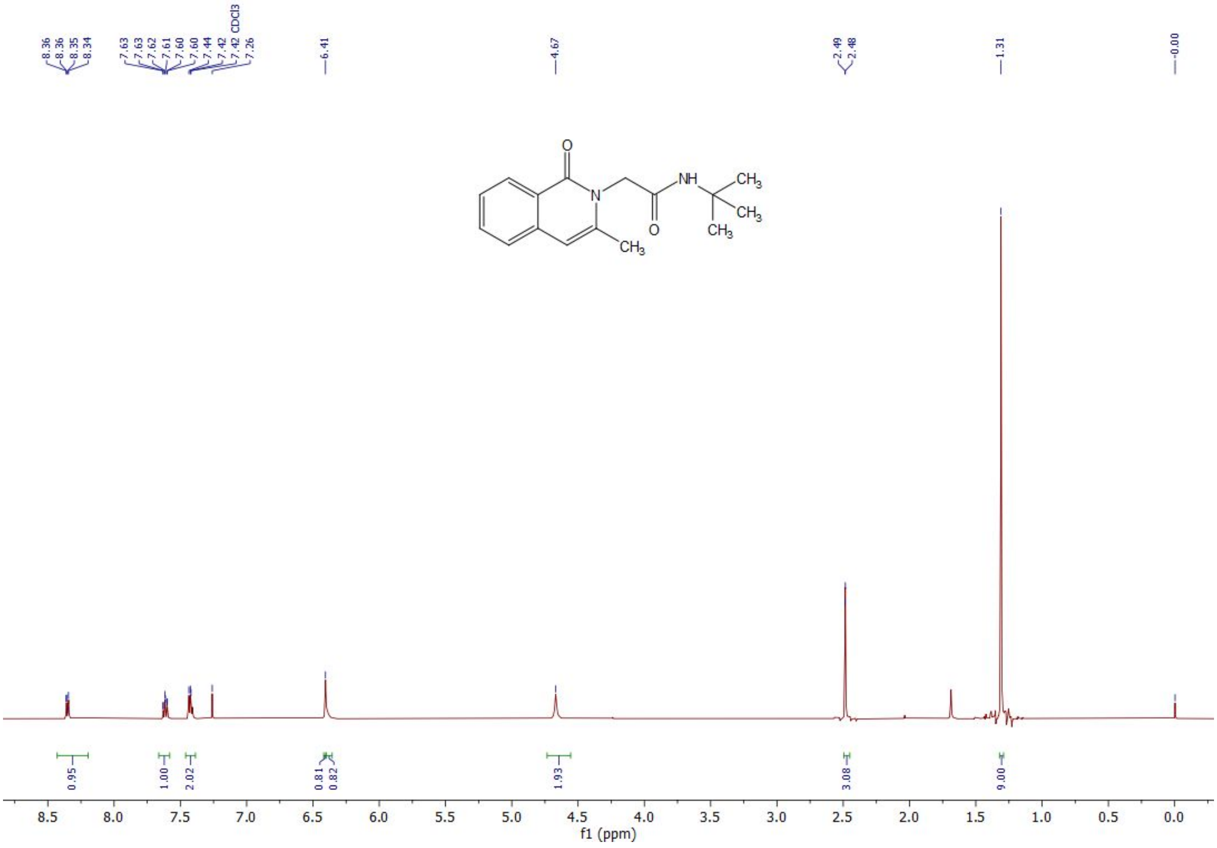

$^{13}C\{^{1}H\}$ NMR spectrum of **3h** (126 MHz, $CDCl_3$)

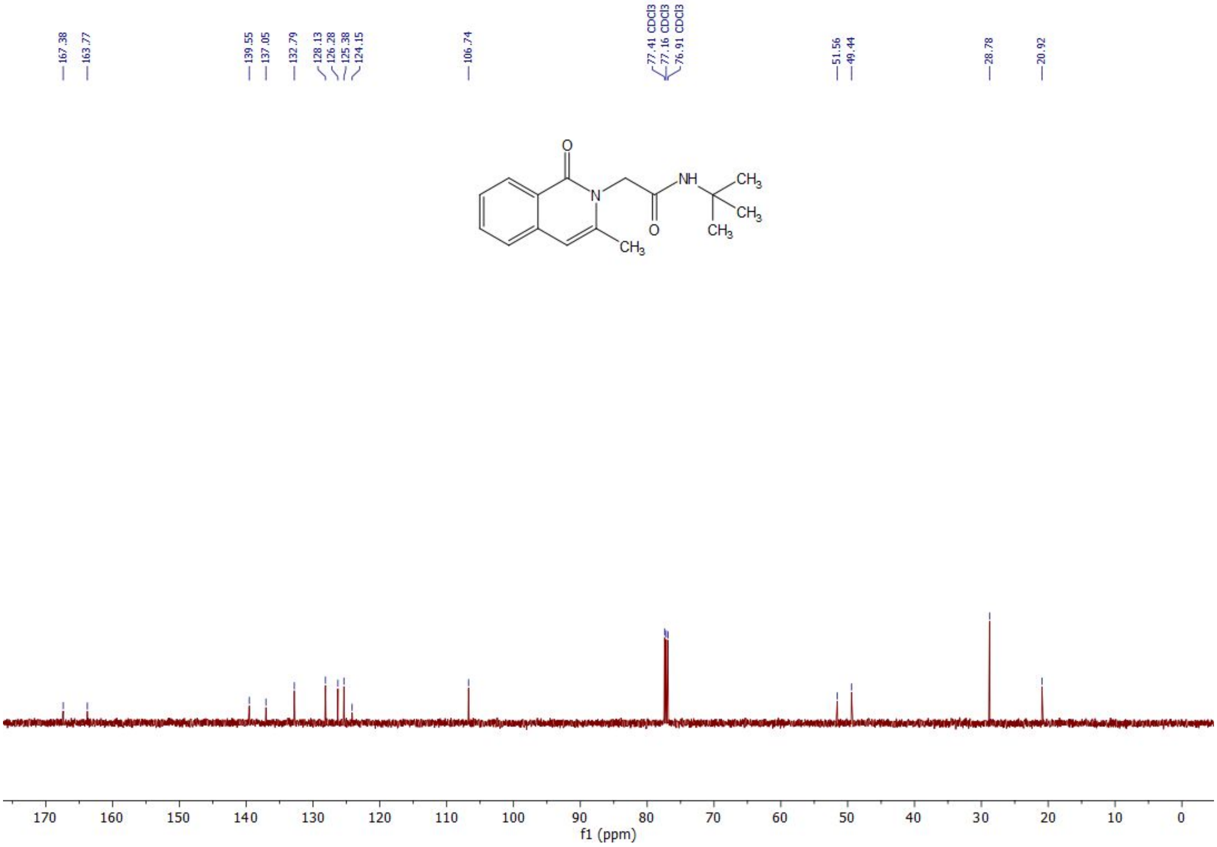

^{1}H NMR spectrum of **3i** (500 MHz, $CDCl_3$)

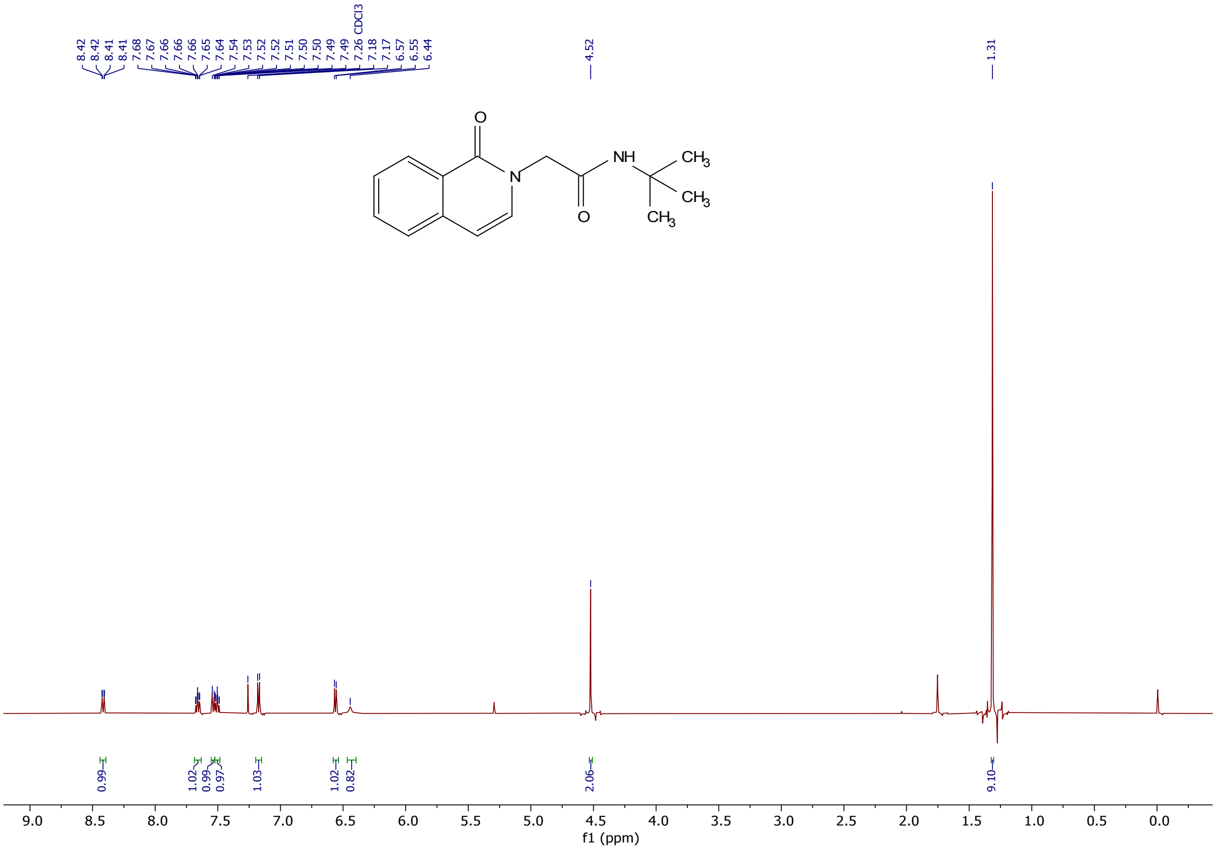

$^{13}C\{^{1}H\}$ NMR spectrum of **3i** (126 MHz, $CDCl_3$)

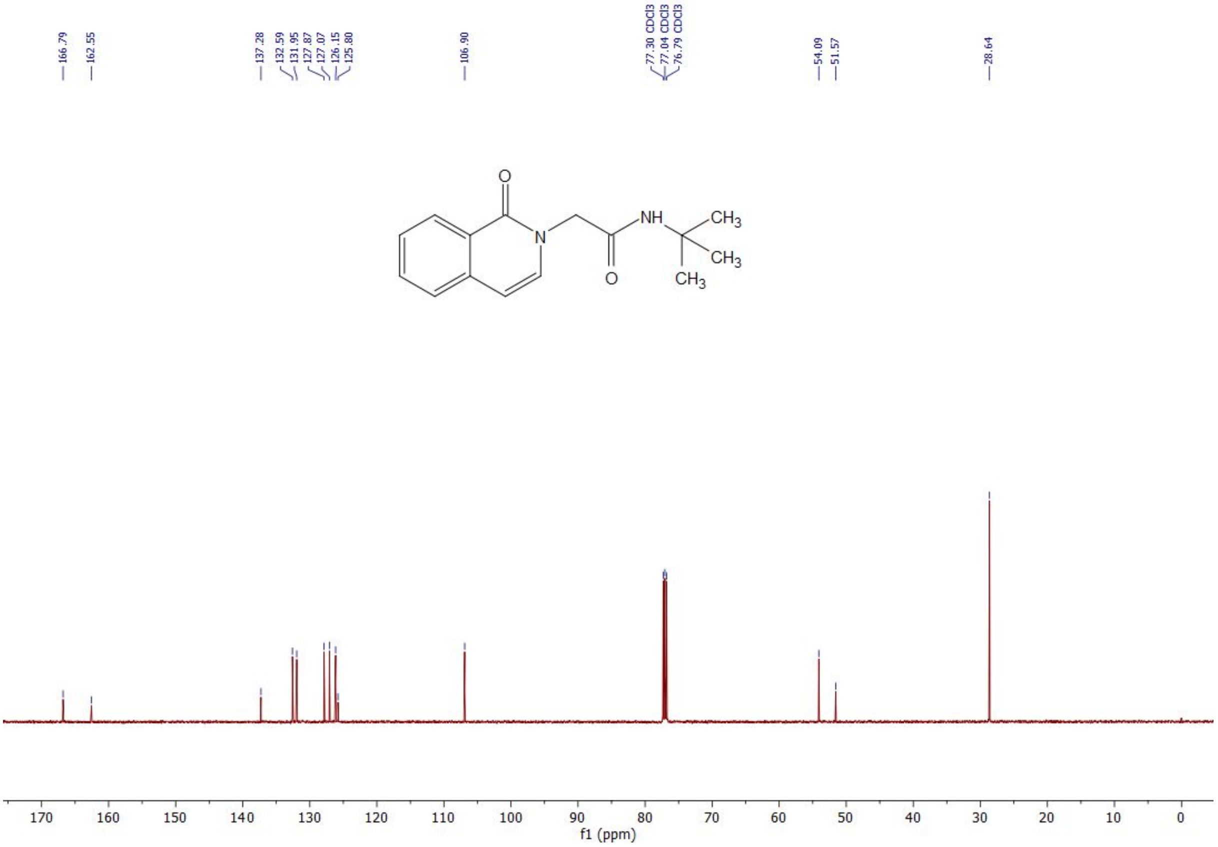

^{1}H NMR spectrum of **3j** (500 MHz, $CDCl_3$)

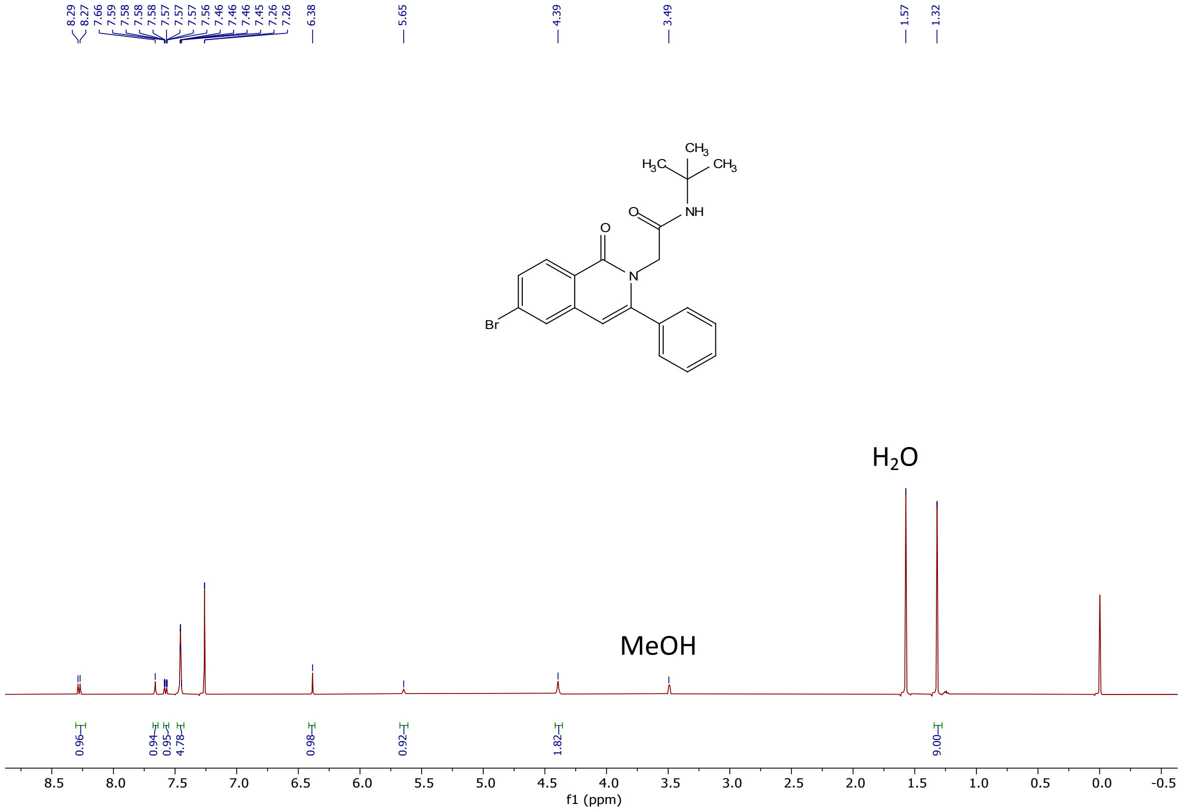

$^{13}C\{^1H\}$ NMR spectrum of **3j** (126 MHz, $CDCl_3$)

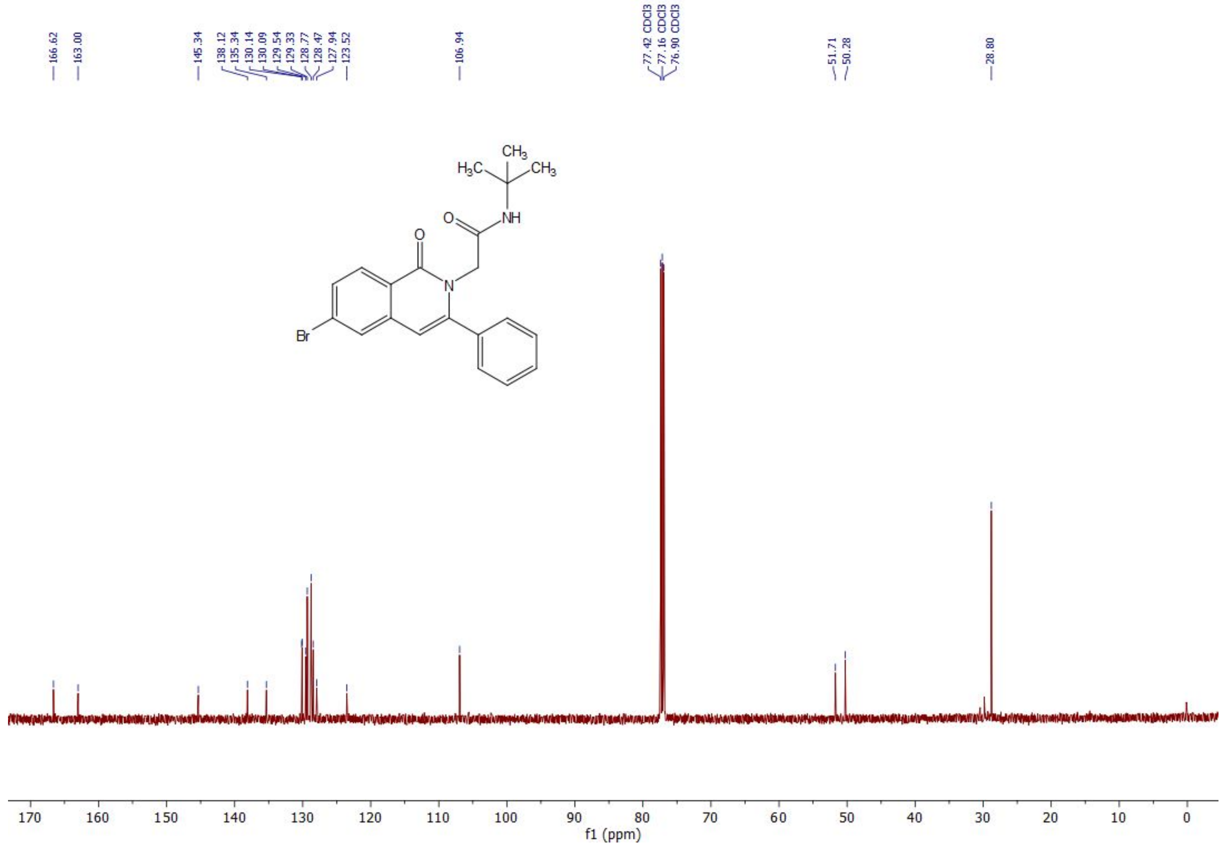

^1H NMR spectrum of **3k** (500 MHz, $CDCl_3$)

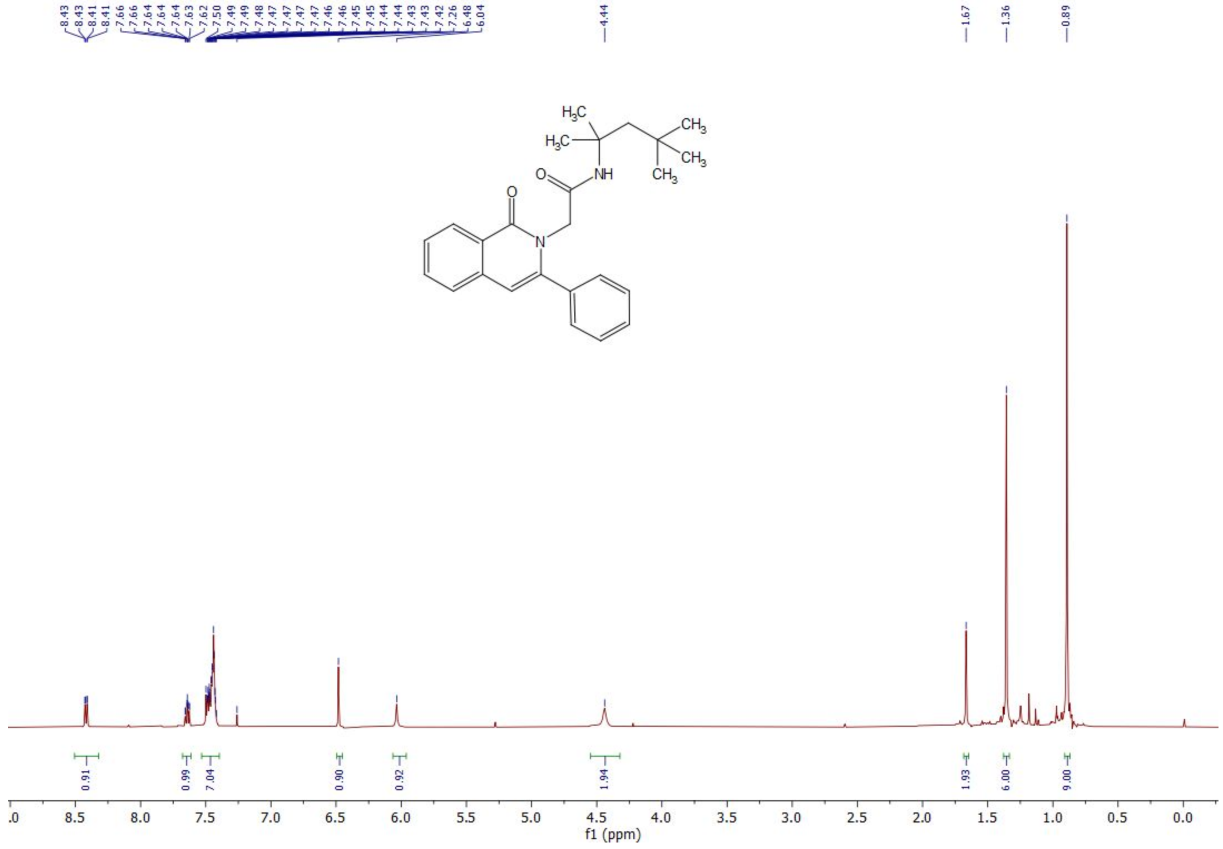

$^{13}C\{^{1}H\}$ NMR spectrum of **3k** (126 MHz, $CDCl_3$)

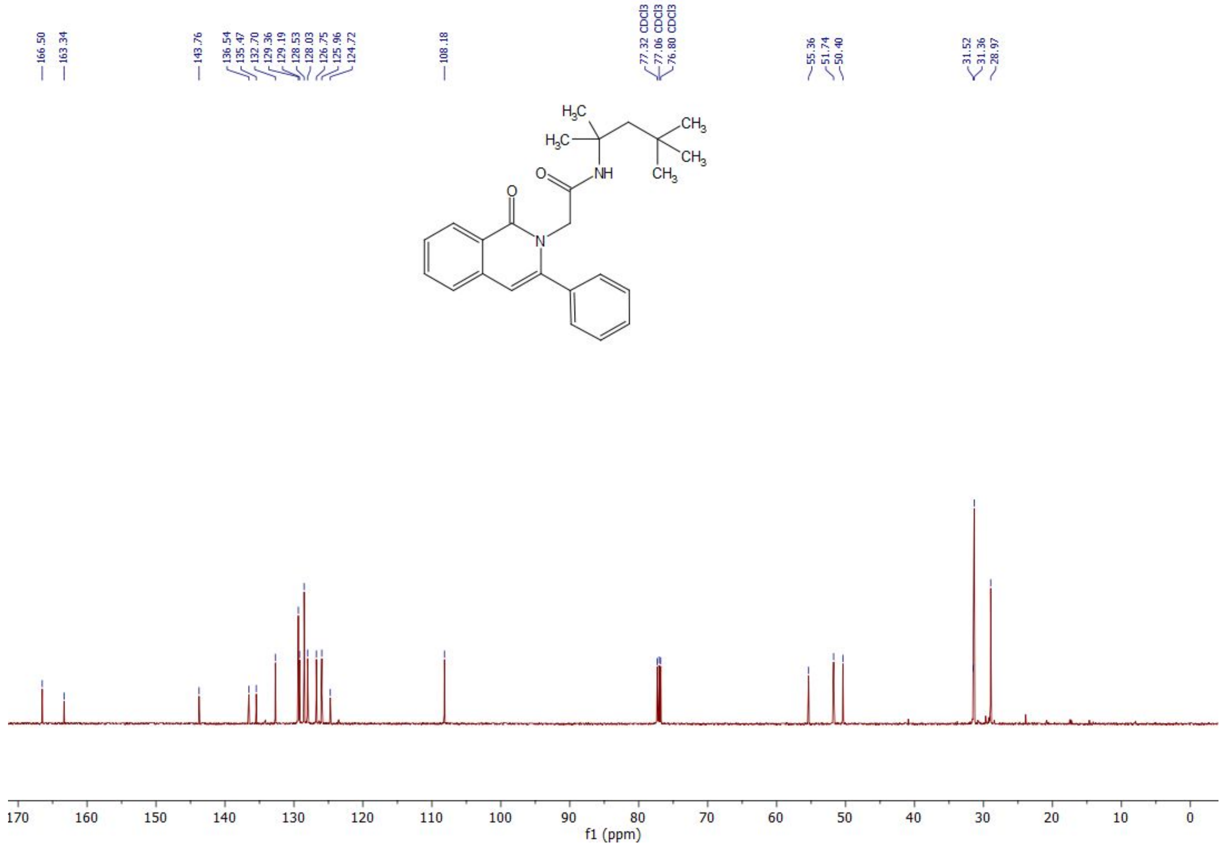

^{1}H NMR spectrum of **3l** (500 MHz, $CDCl_3$)

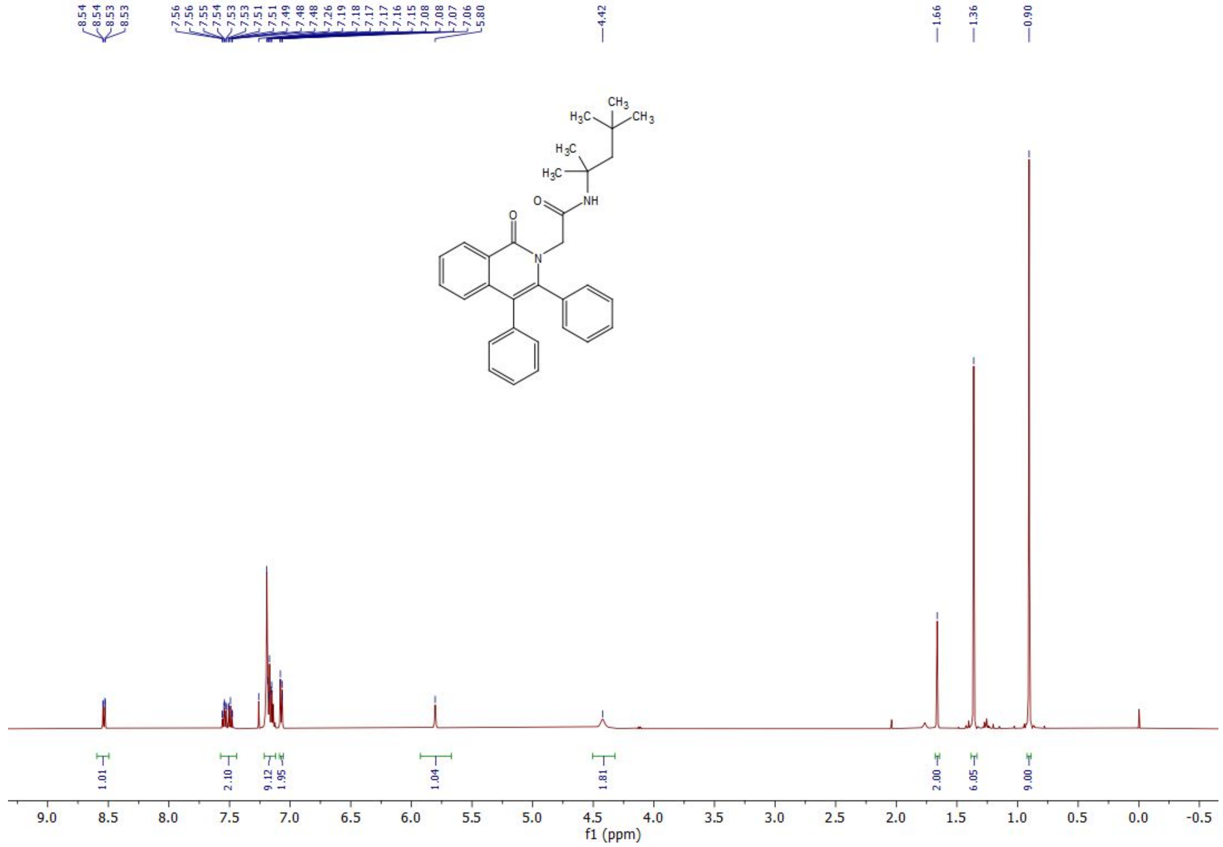

$^{13}C\{^{1}H\}$ NMR spectrum of **3l** (126 MHz, $CDCl_3$)

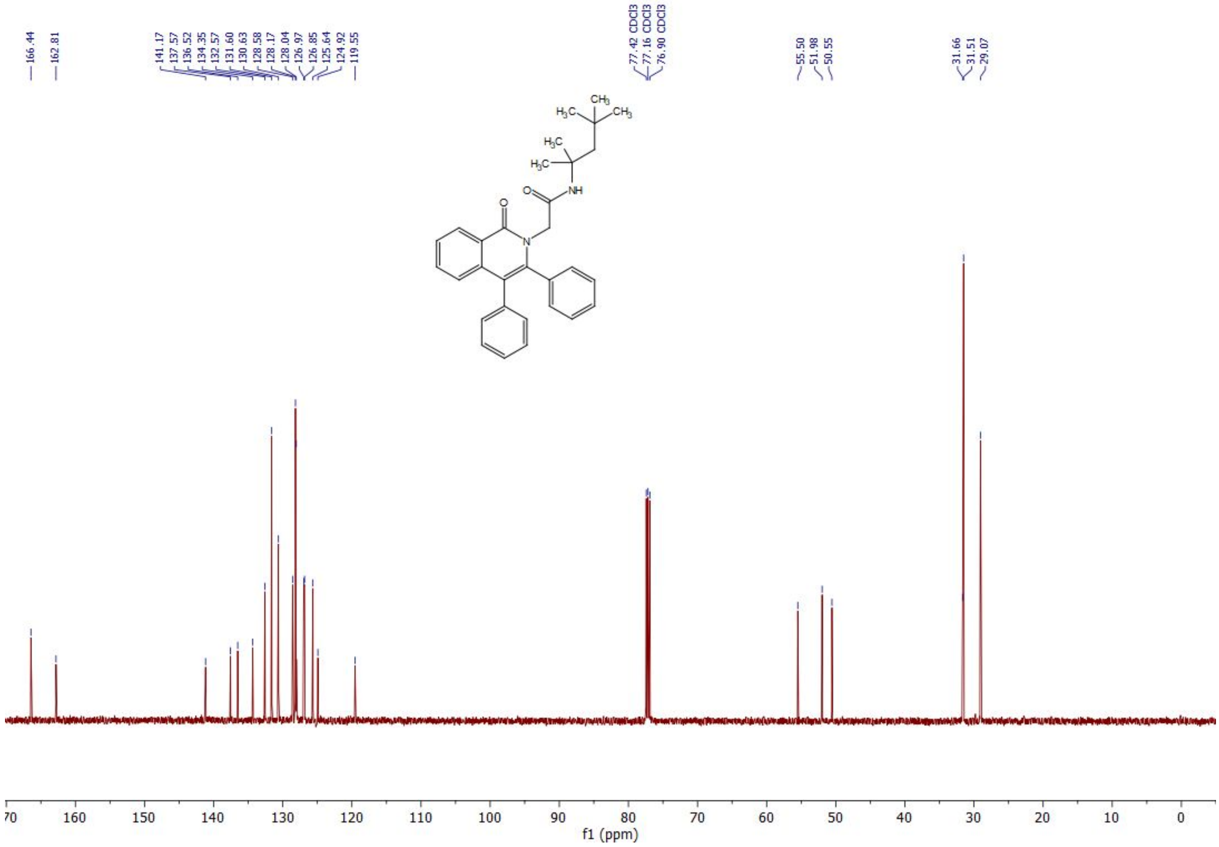

^{1}H NMR spectrum of **3m** (500 MHz, $CDCl_3$)

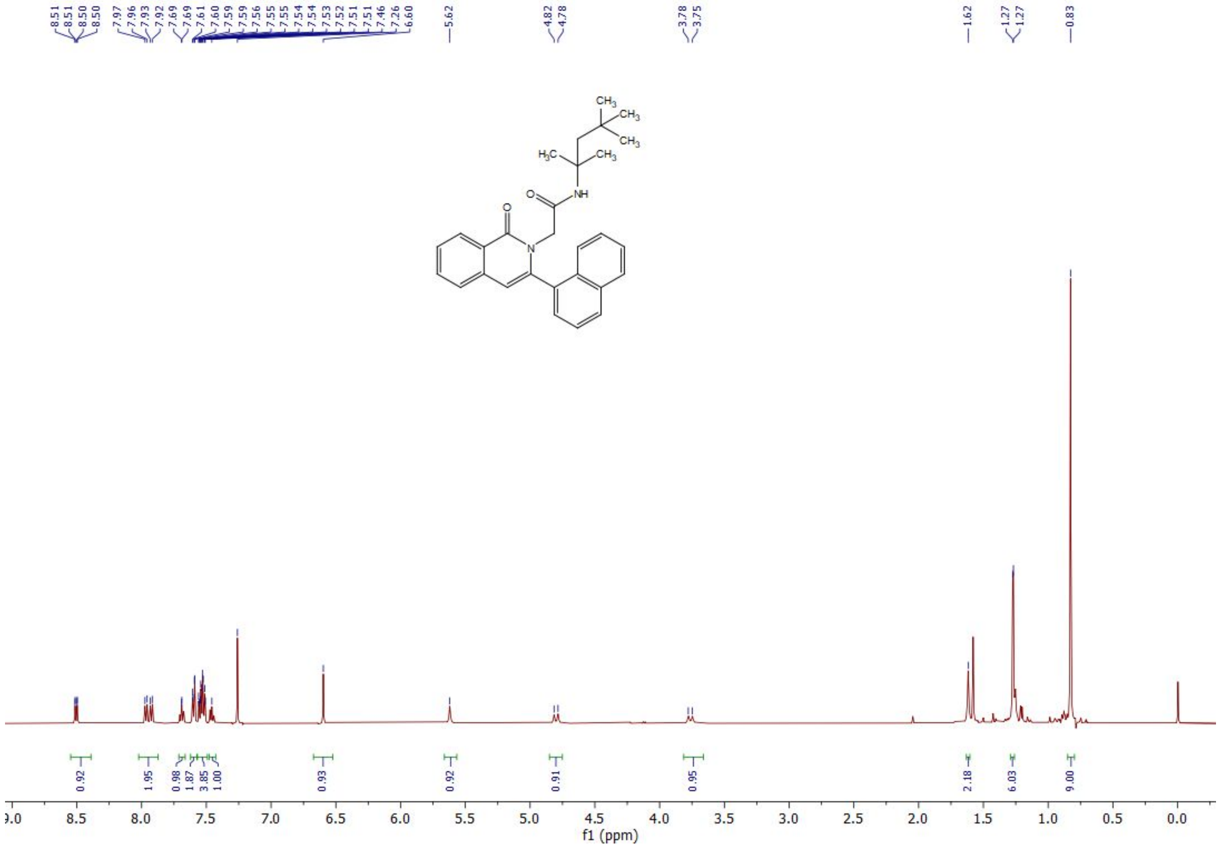

$^{13}C\{^1H\}$ NMR spectrum of **3m** (126 MHz, $CDCl_3$)

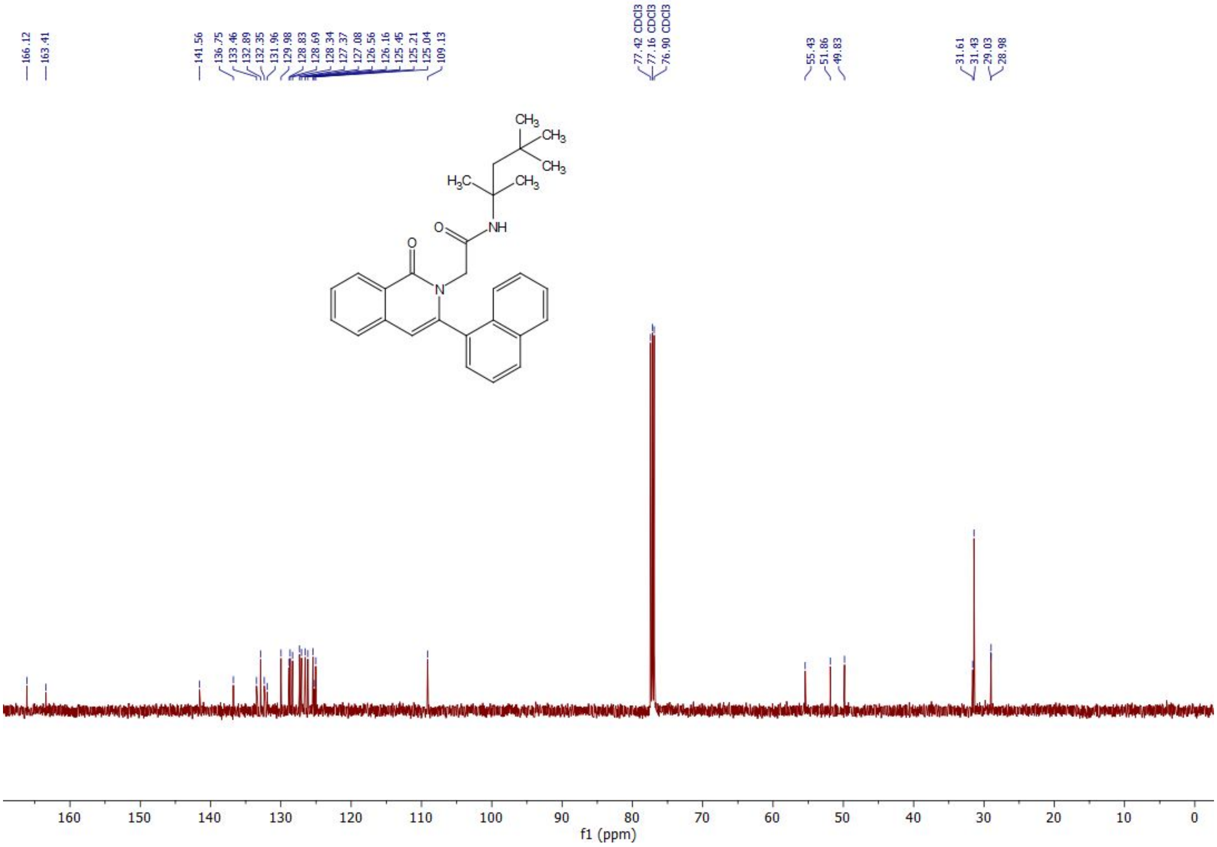

^1H NMR spectrum of **3n** (500 MHz, $CDCl_3$)

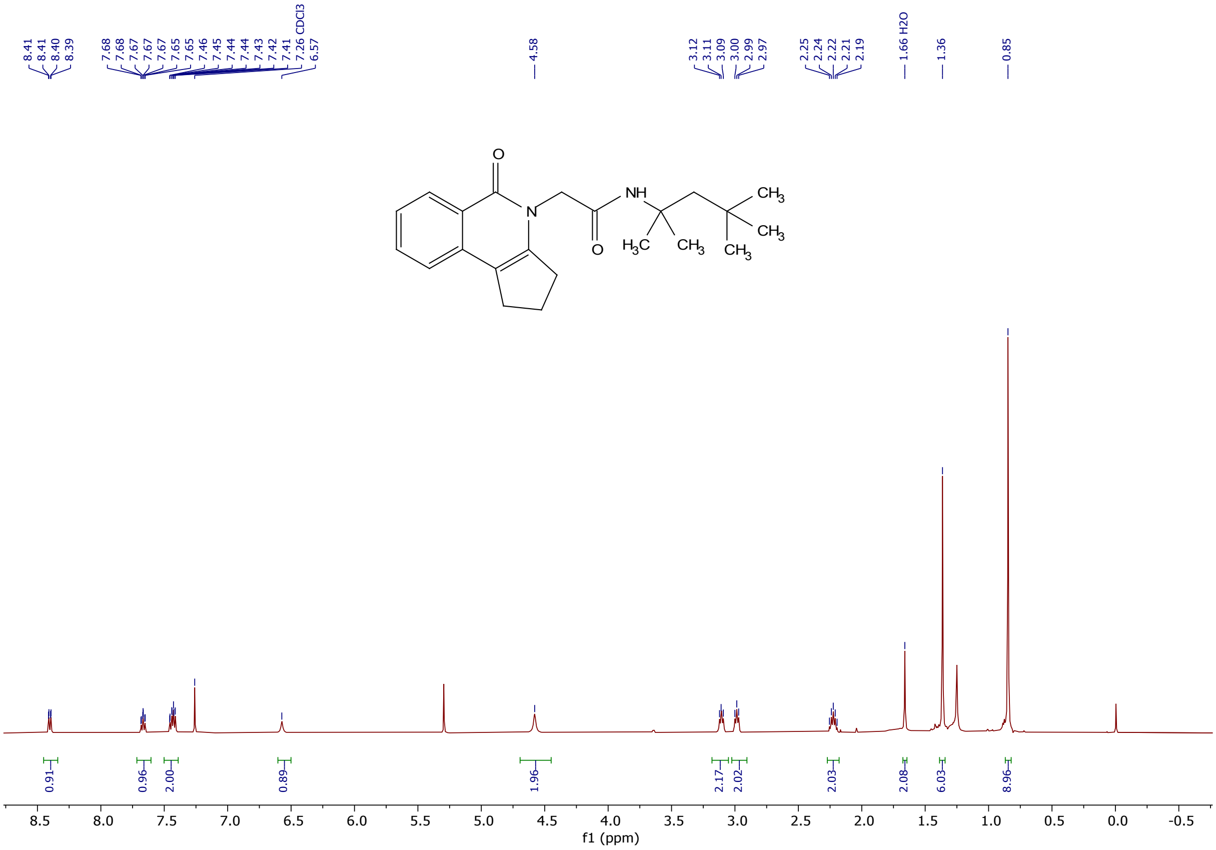

$^{13}C\{^1H\}$ NMR spectrum of **3n** (126 MHz, $CDCl_3$)

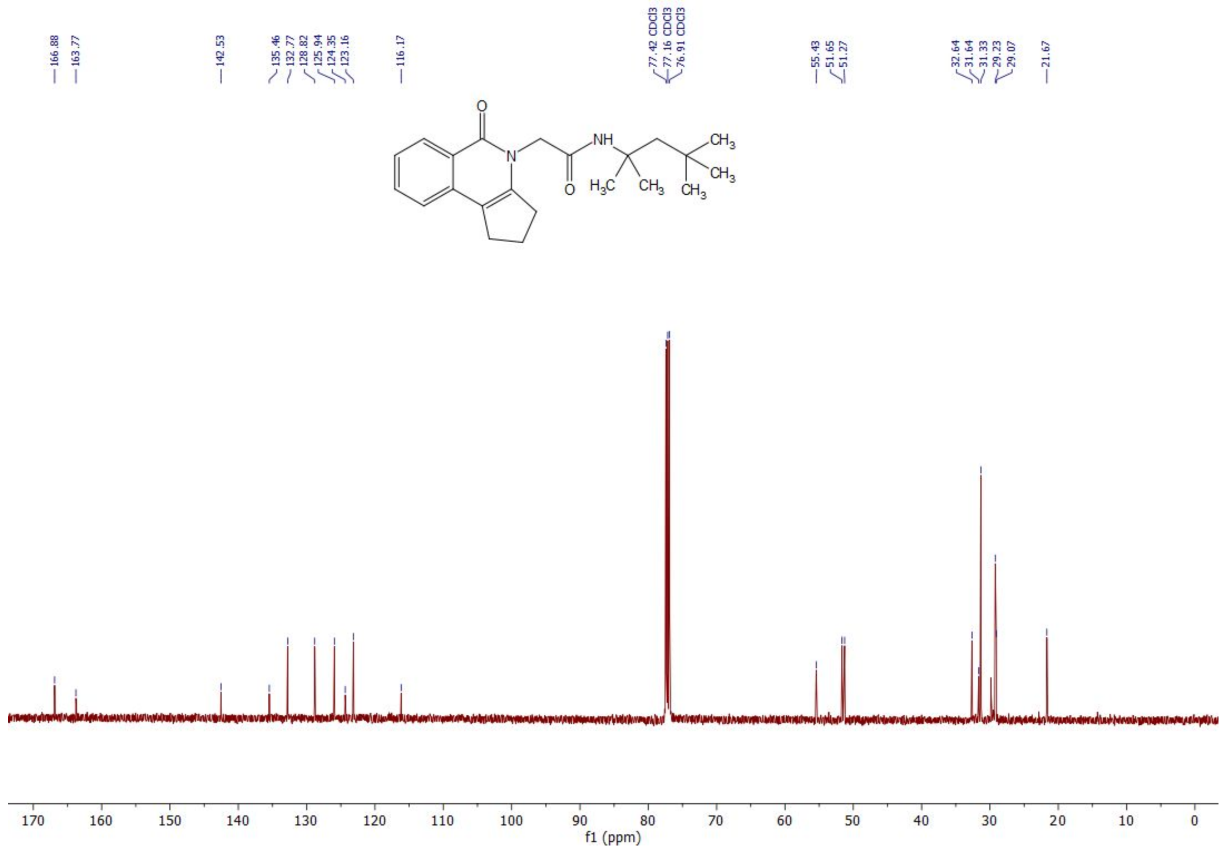

^1H NMR spectrum of **3o** (500 MHz, $CDCl_3$)

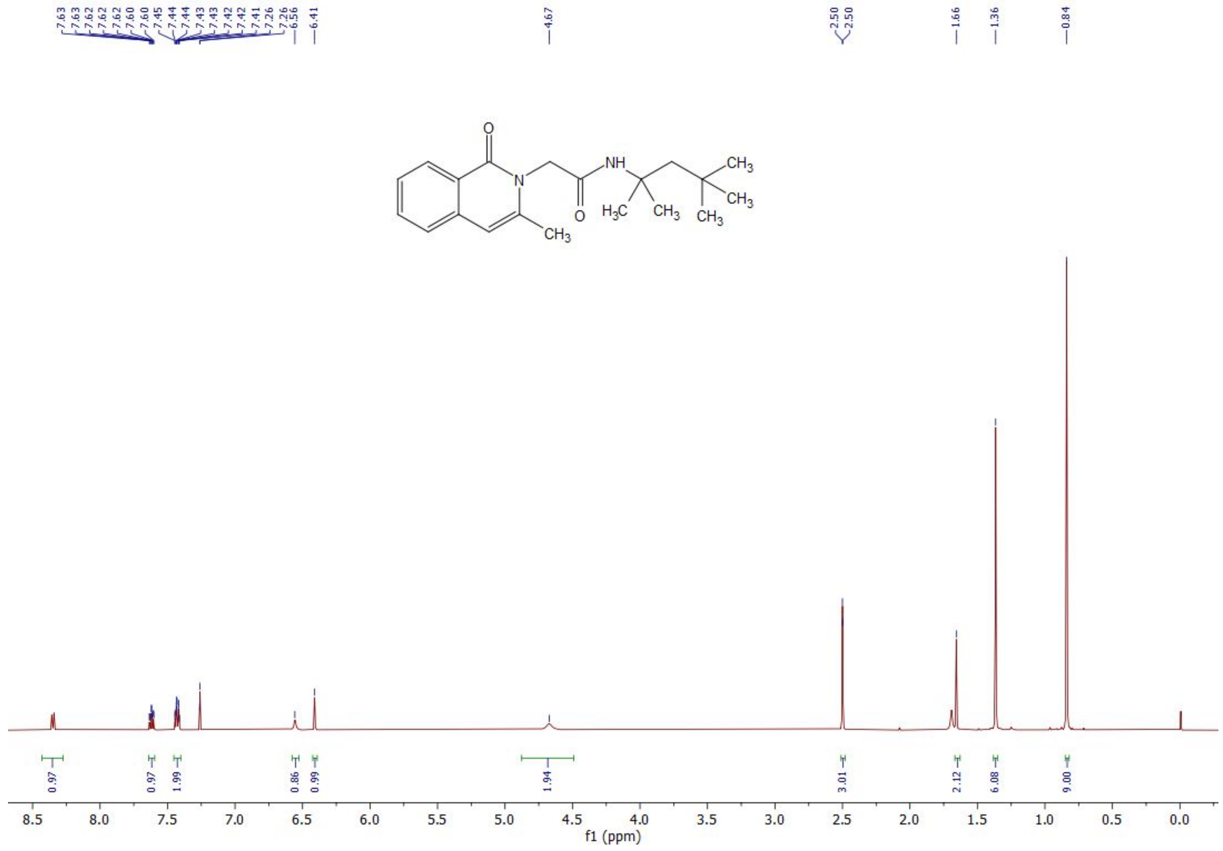

$^{13}C\{^{1}H\}$ NMR spectrum of **3o** (126 MHz, $CDCl_3$)

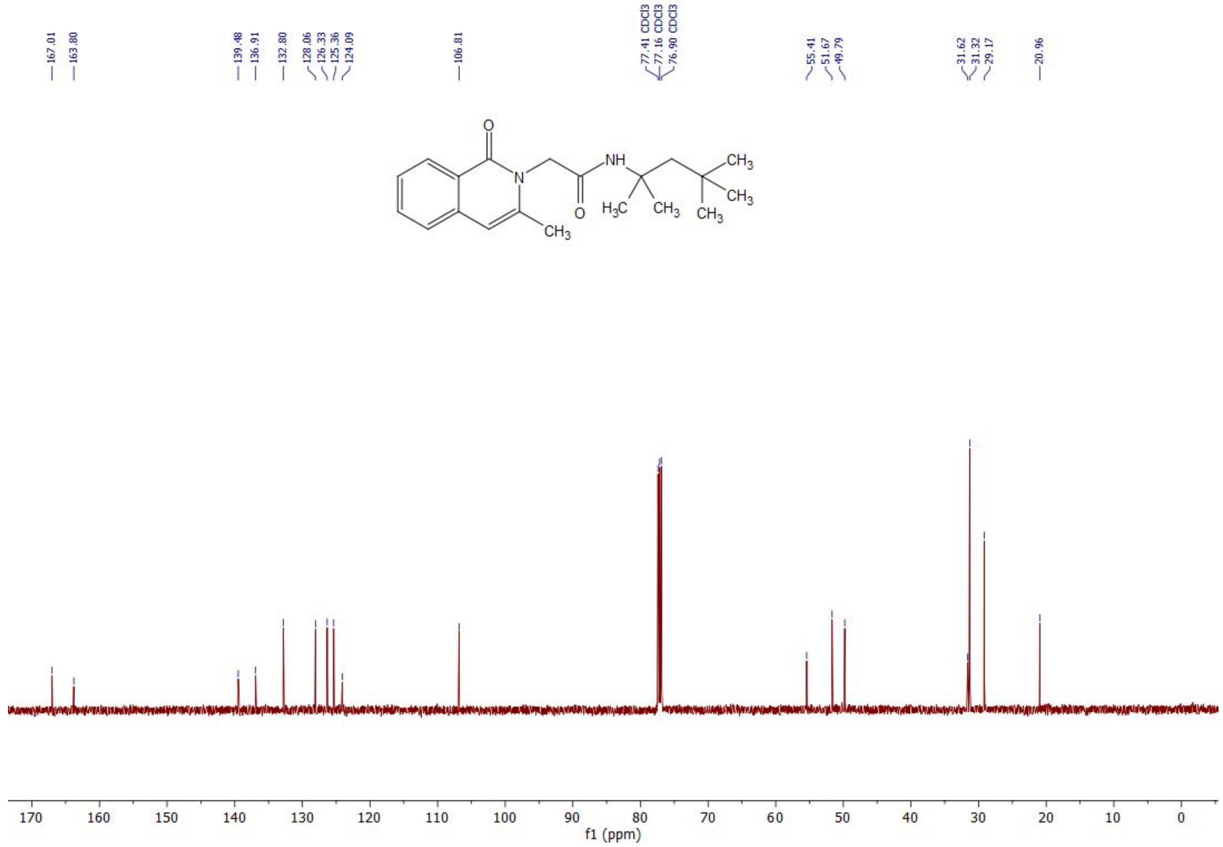

^{1}H NMR spectrum of **3p** (500 MHz, $CDCl_3$)

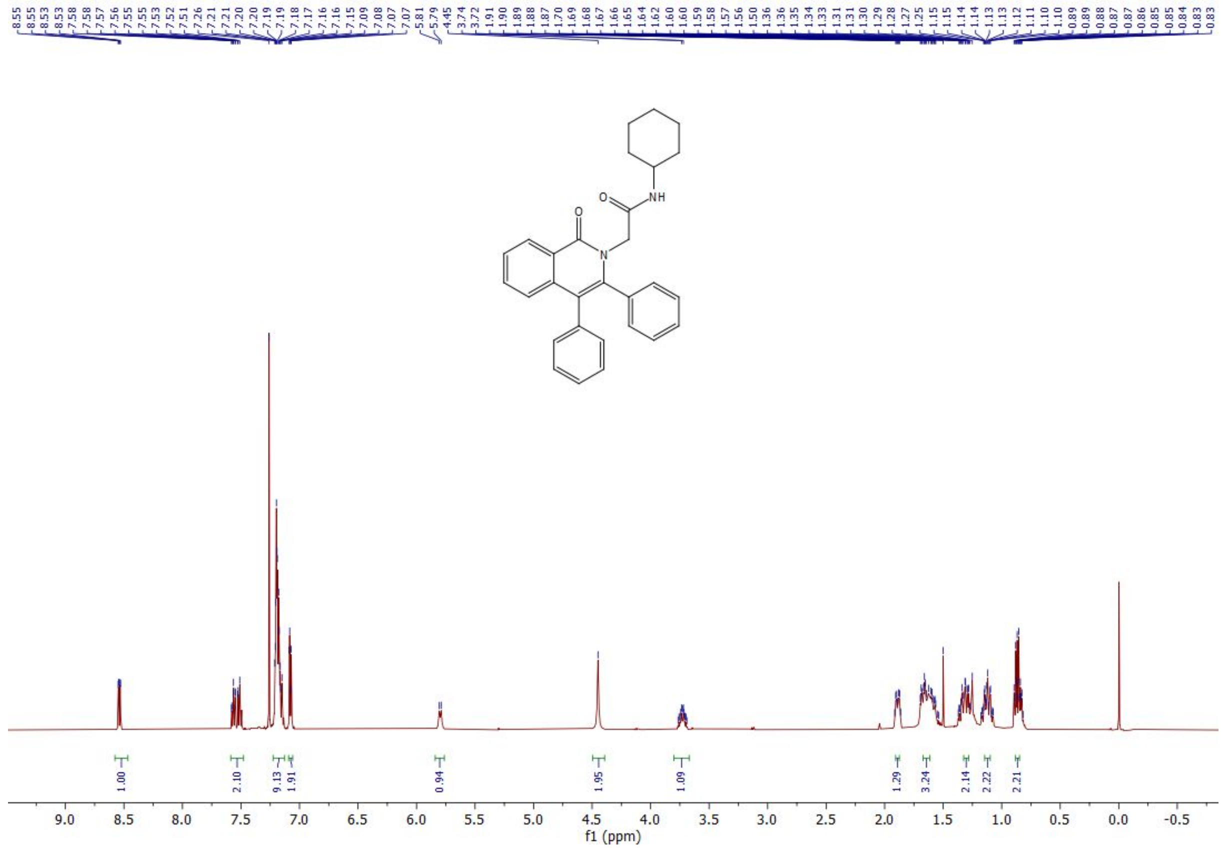

$^{13}C\{^1H\}$ NMR spectrum of **3p** (126 MHz, $CDCl_3$)

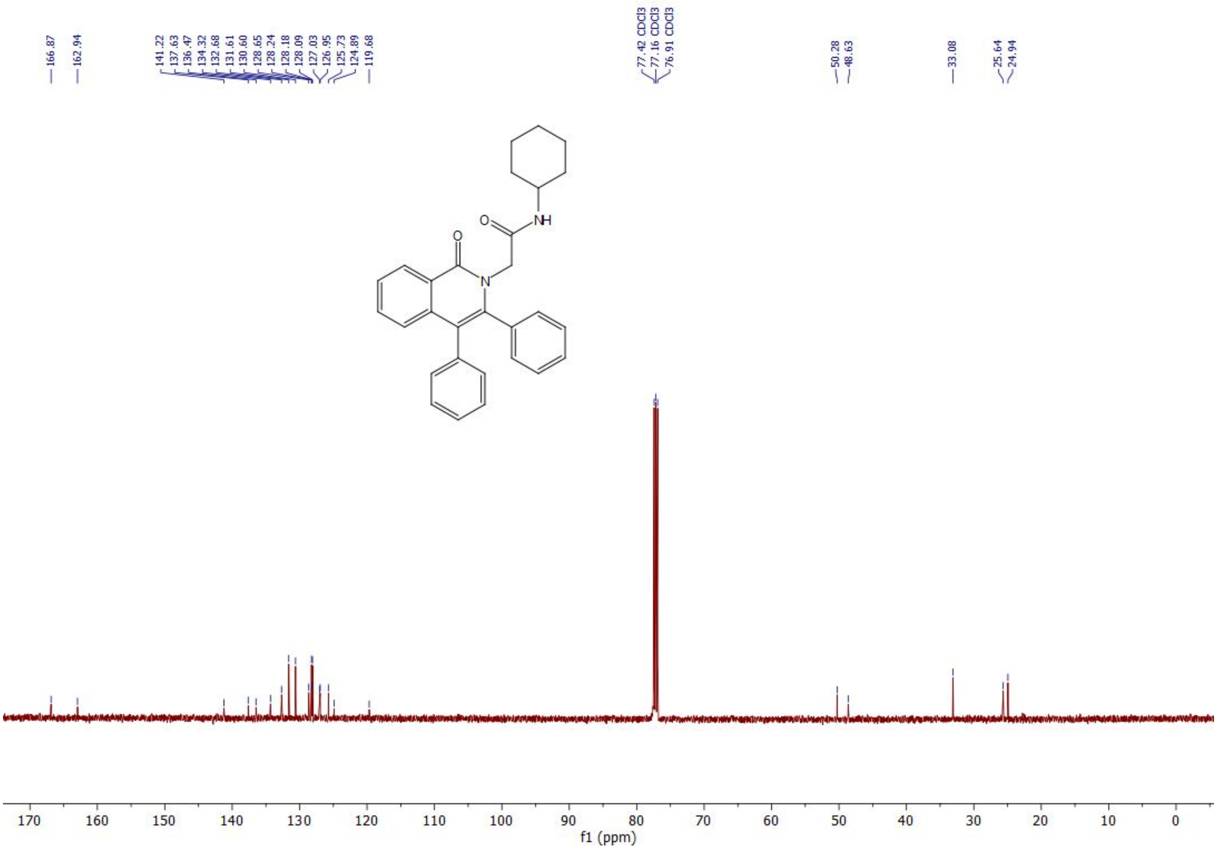

^1H NMR spectrum of **5a** (500 MHz, $CDCl_3$)

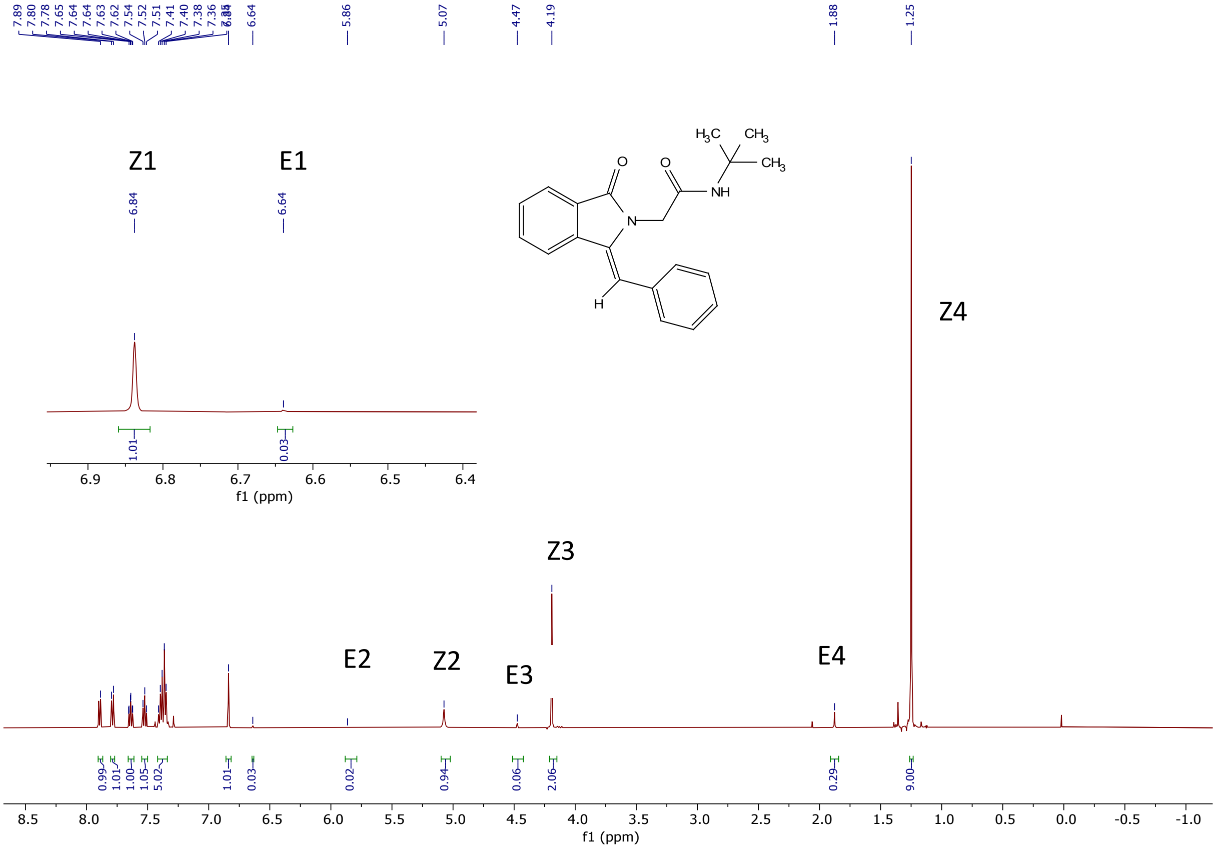

$^{13}C\{^1H\}$ NMR spectrum of **5a** (126 MHz, $CDCl_3$)

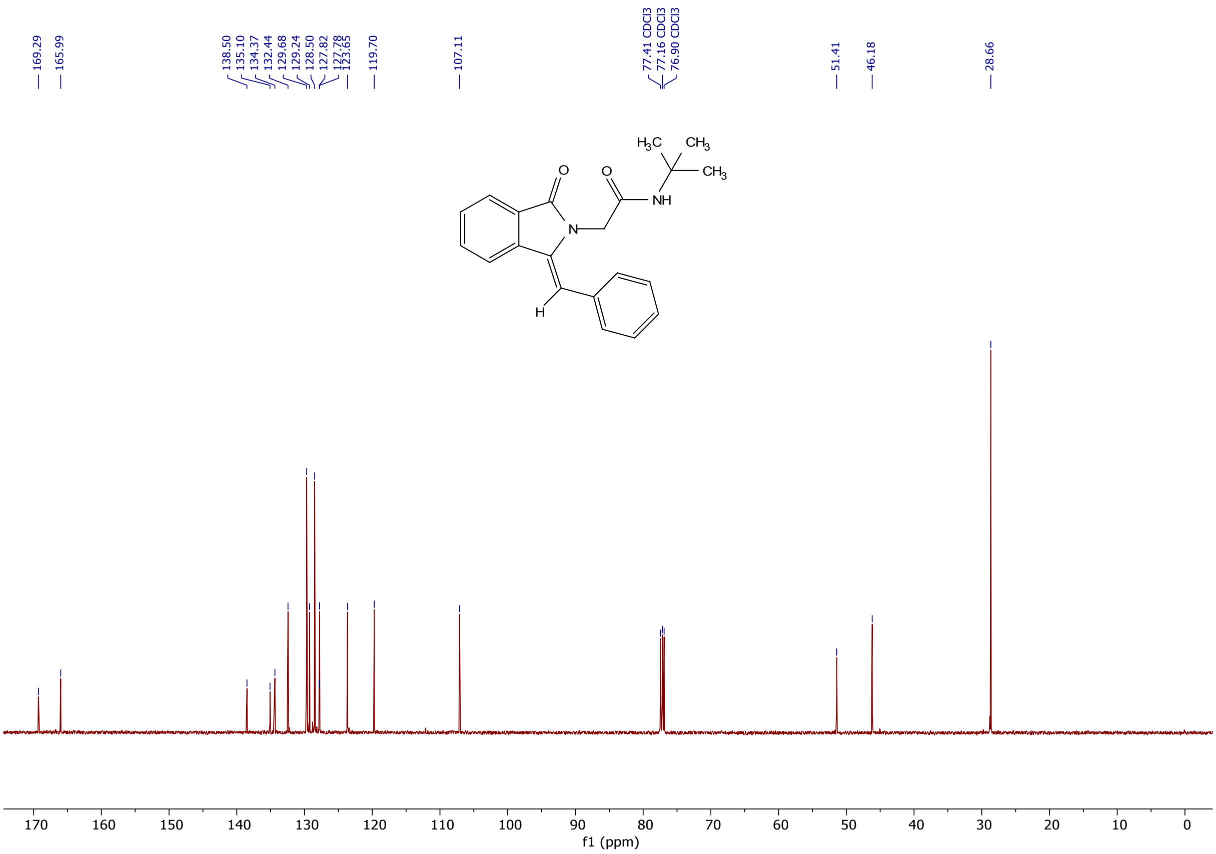

HMBC spectrum of **5a**

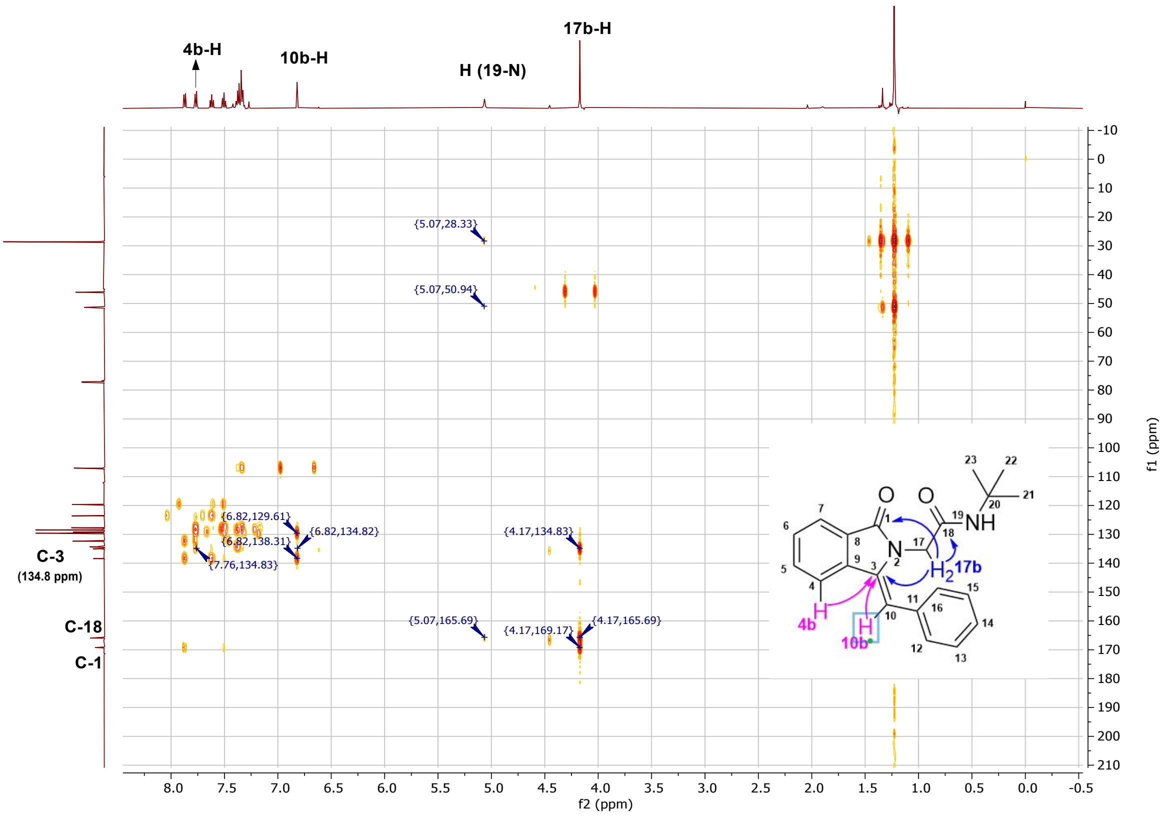

^{1}H NMR spectrum of **5b** (500 MHz, $CDCl_3$)

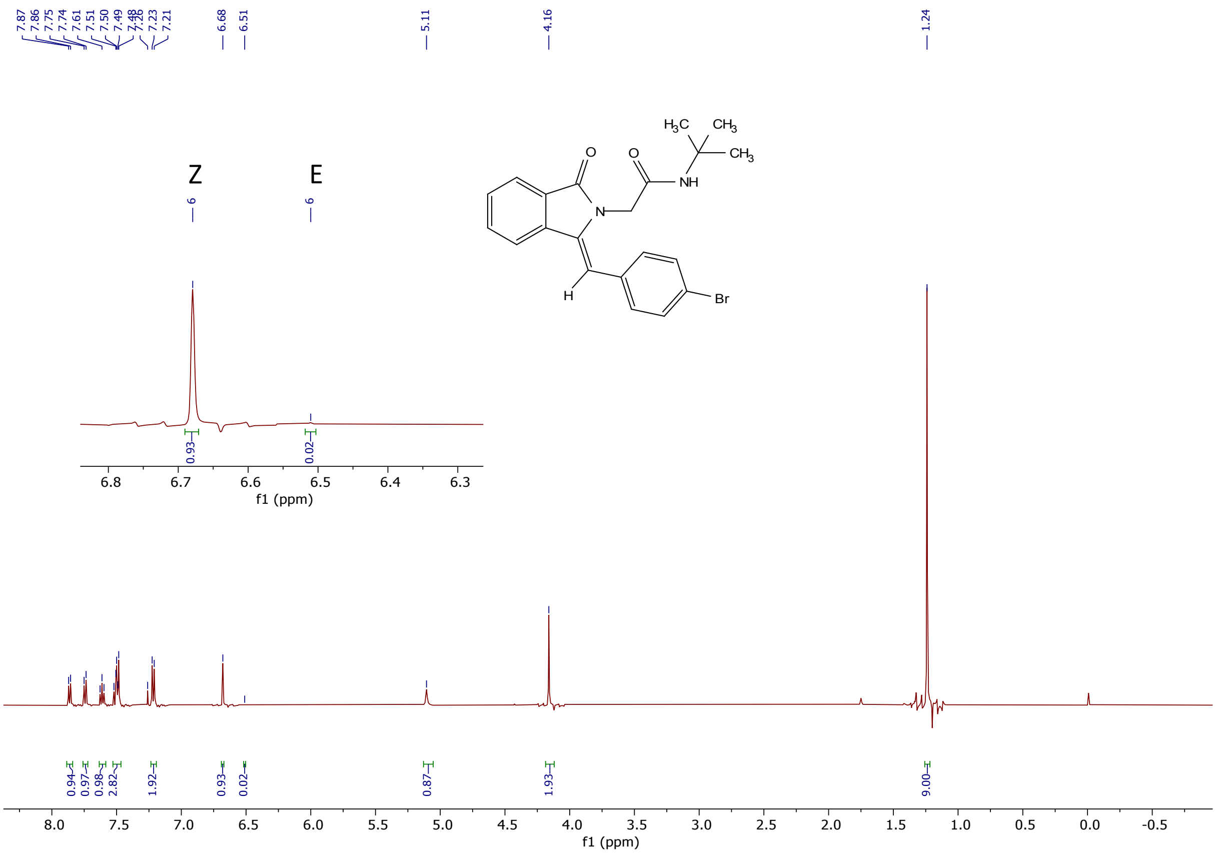

$^{13}C\{^{1}H\}$ NMR spectrum of **5b** (126 MHz, $CDCl_3$)

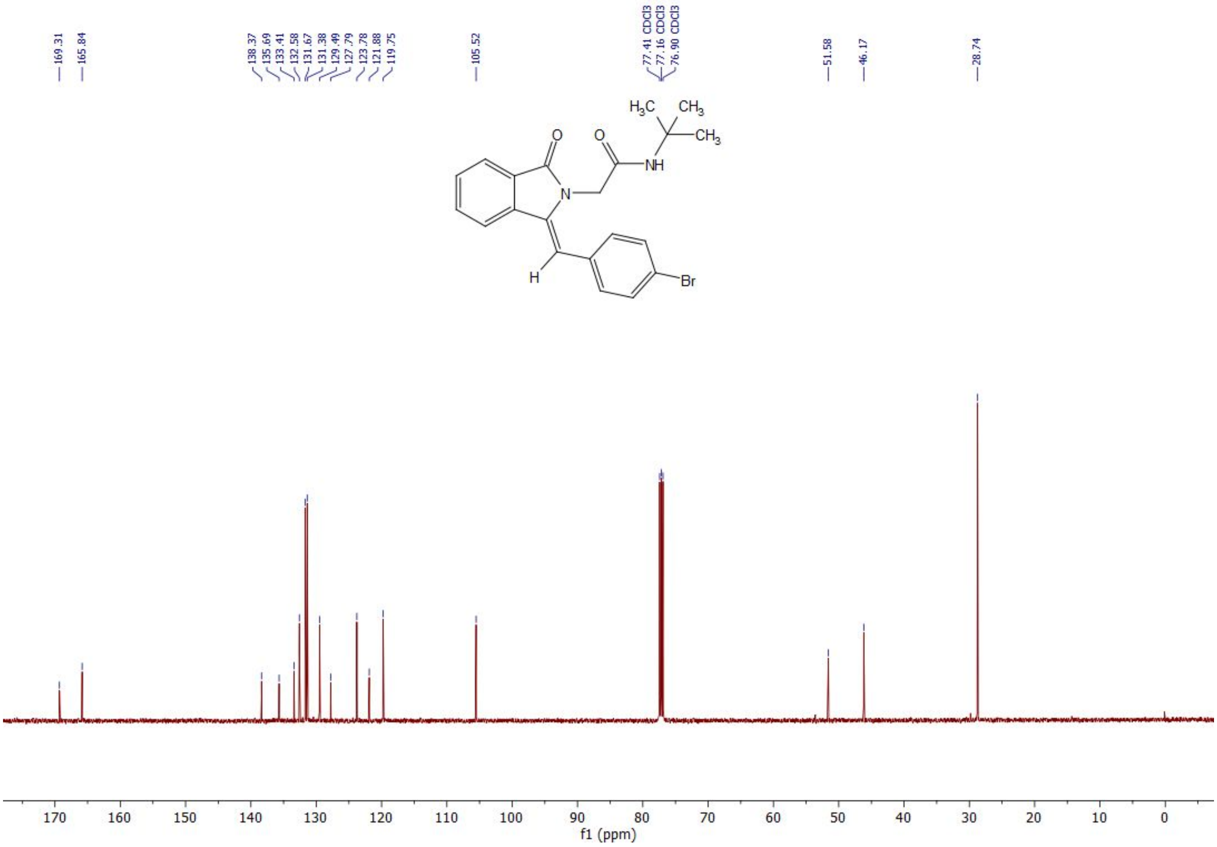

^{1}H NMR spectrum of **5c** (500 MHz, $CDCl_3$)

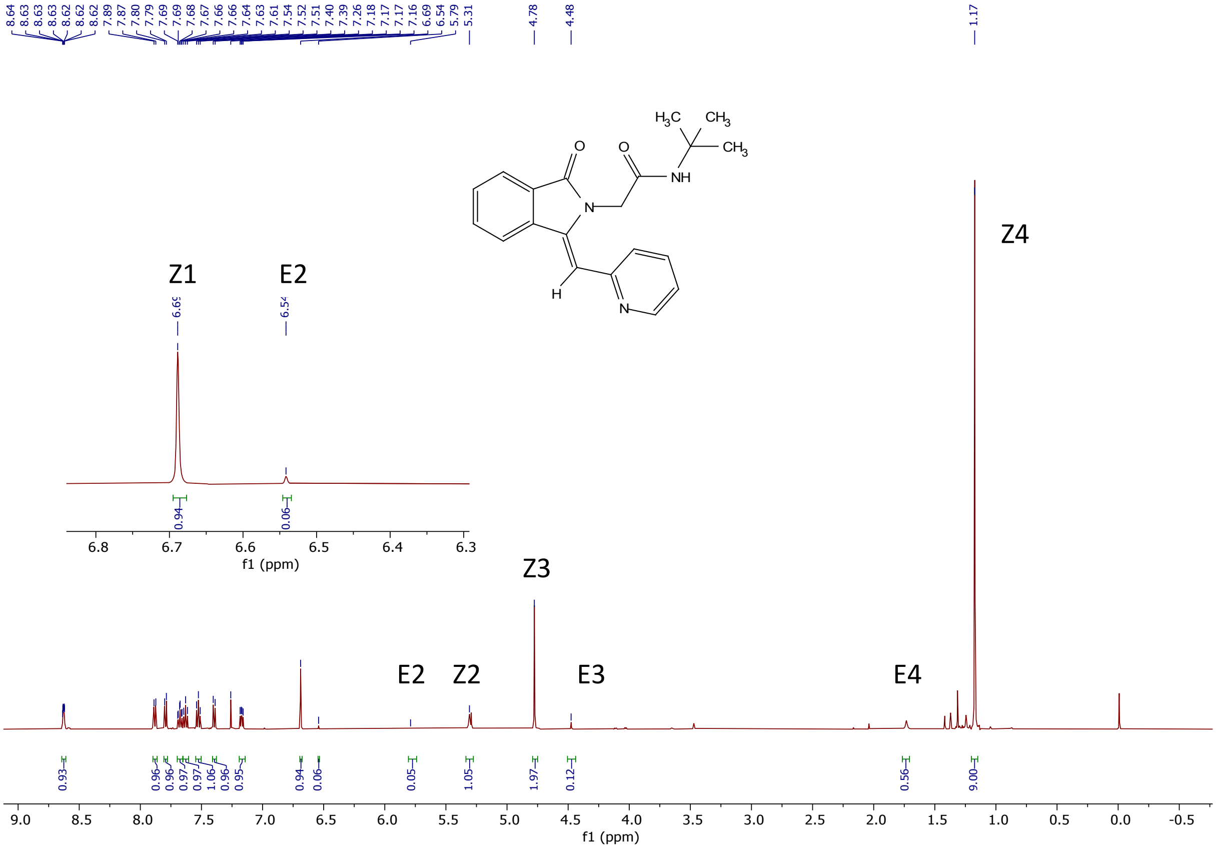

^{13}C{^{1}H} NMR spectrum of **5c** (126 MHz, $CDCl_3$)

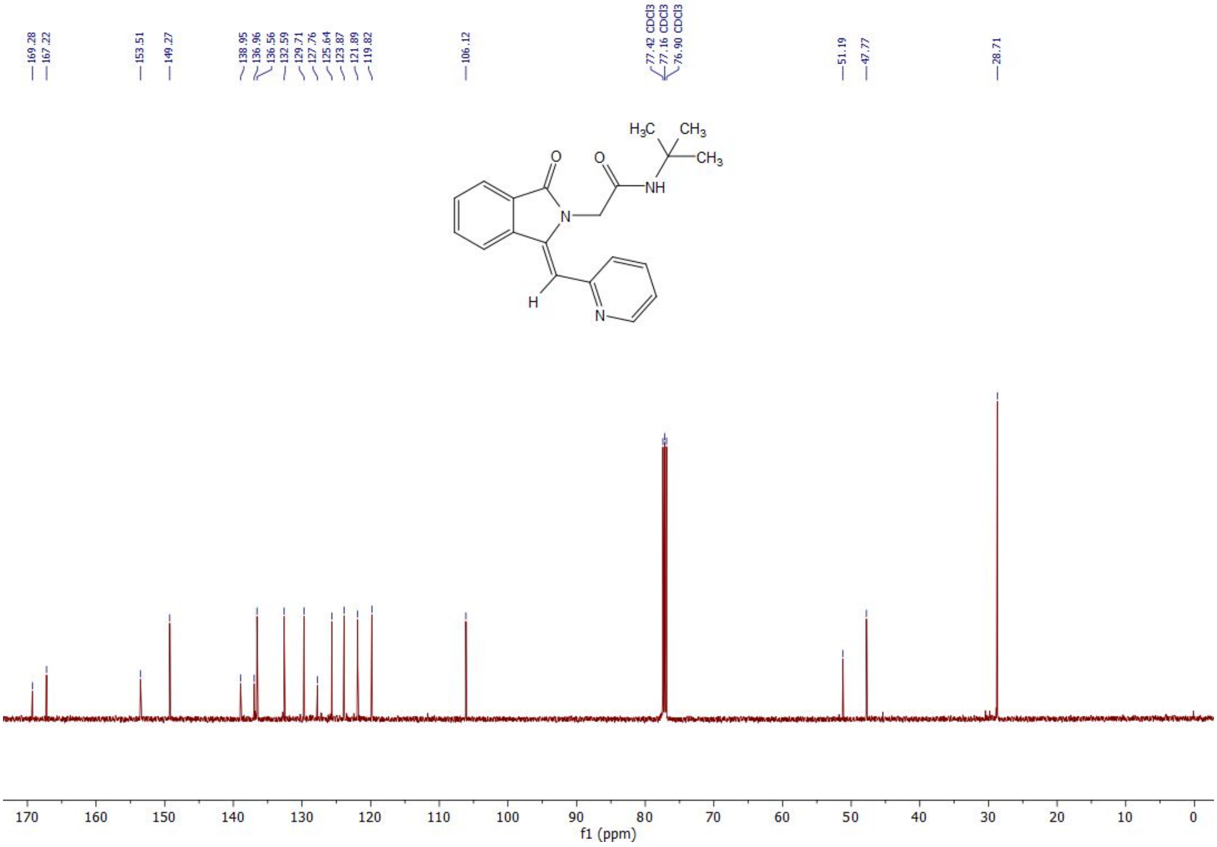

^{1}H NMR spectrum of **5d** (500 MHz, $CDCl_3$)

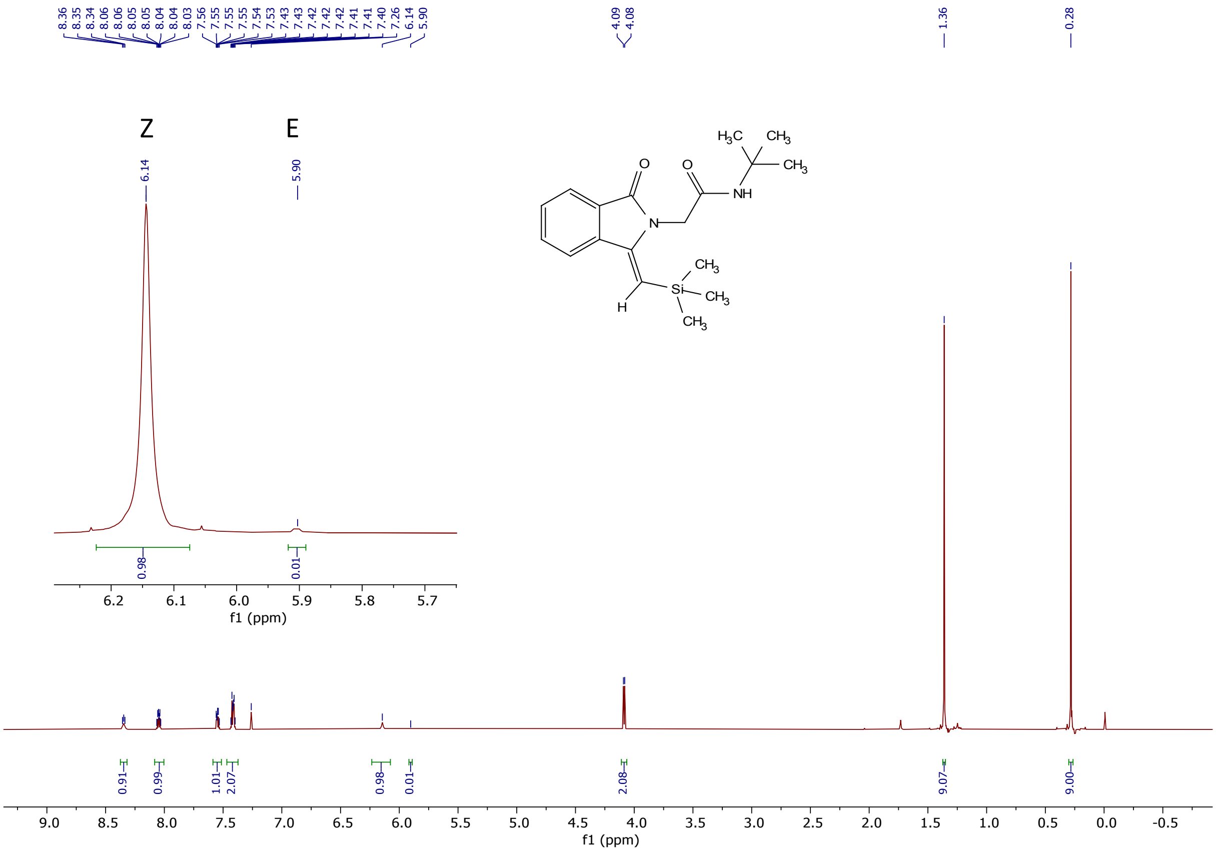

$^{13}C\{^{1}H\}$ NMR spectrum of **5d** (126 MHz, $CDCl_3$)

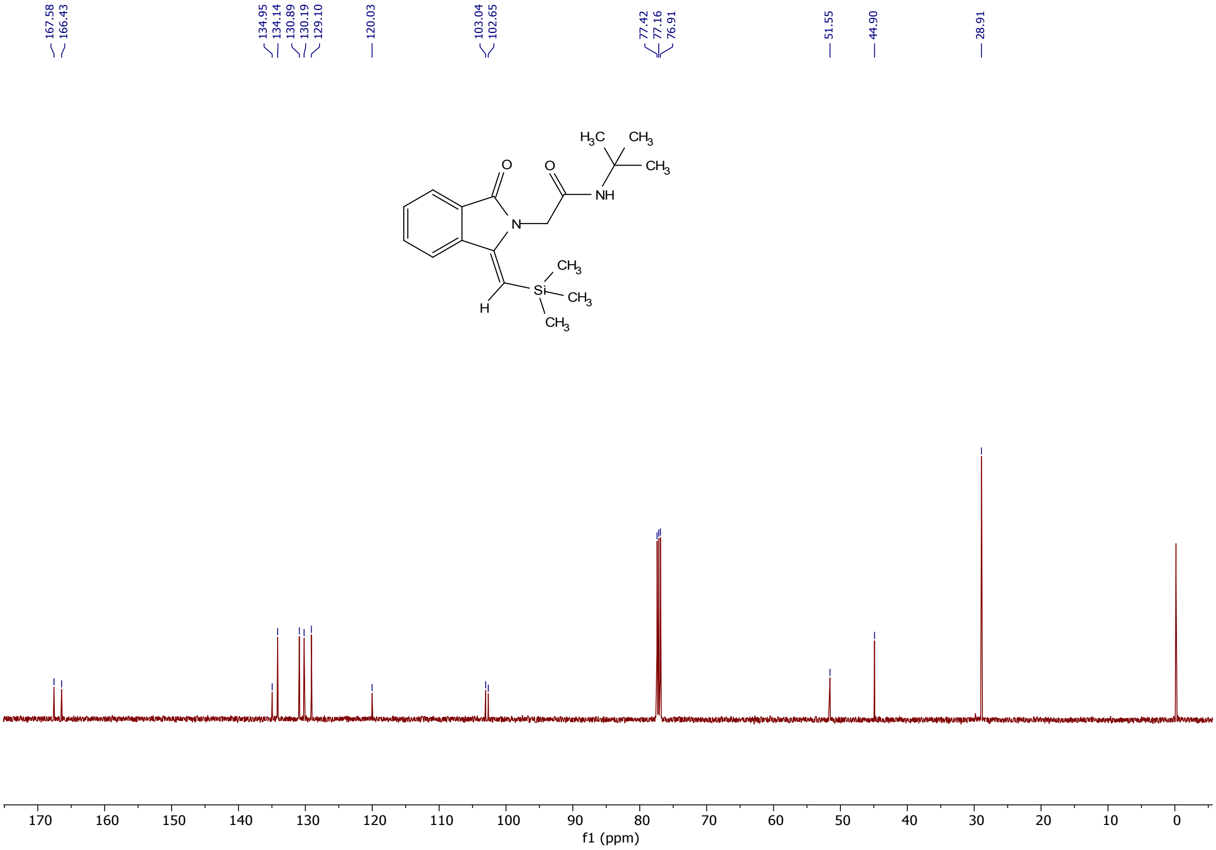

^{1}H NMR spectrum of **5e** (500 MHz, $CDCl_3$)

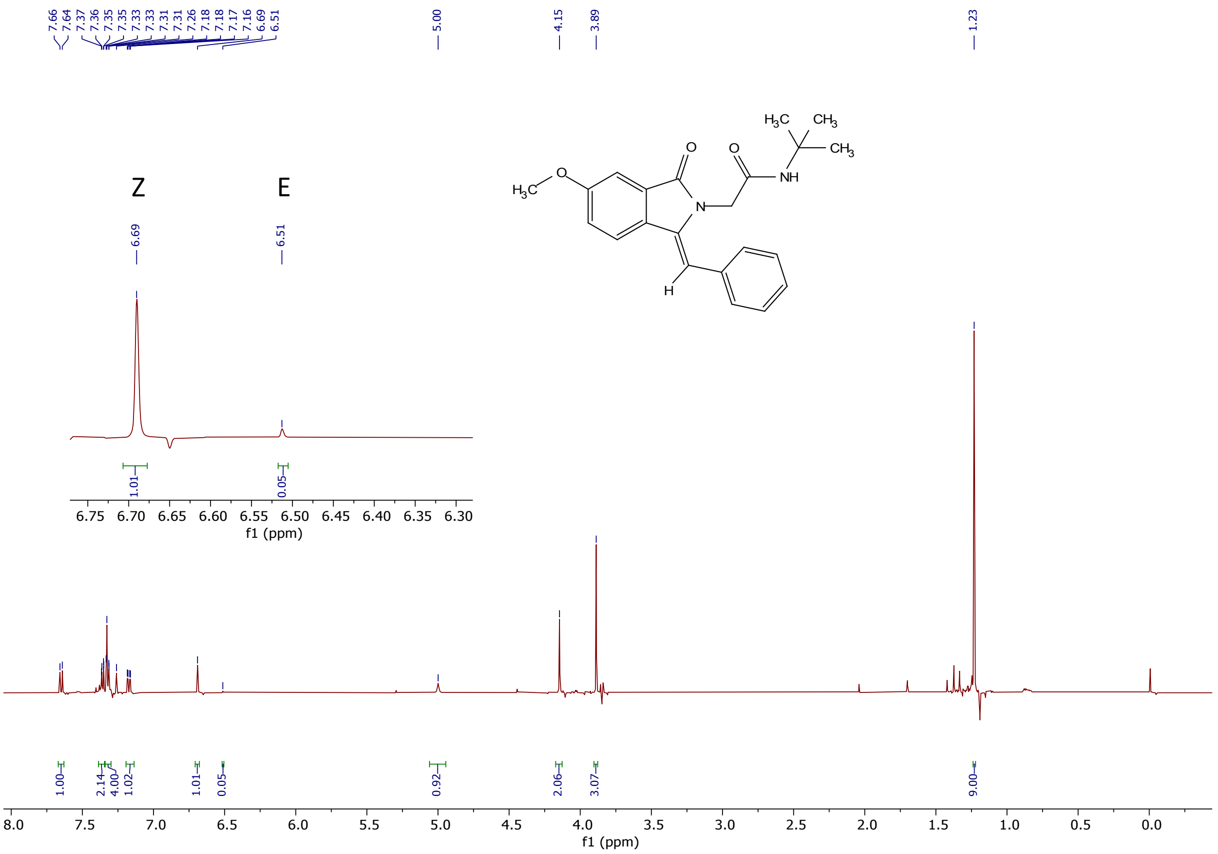

$^{13}C\{^{1}H\}$ NMR spectrum of **5e** (126 MHz, $CDCl_3$)

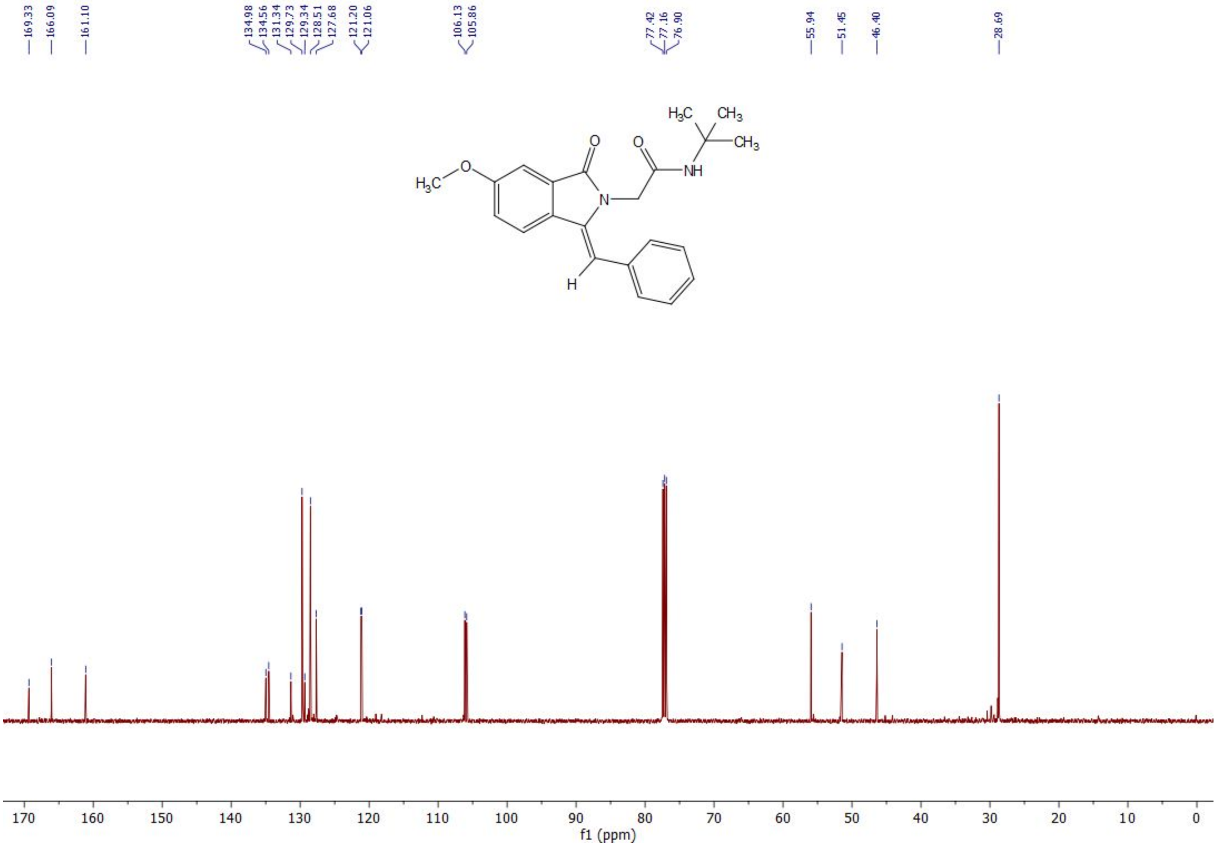

^{1}H NMR spectrum of **5f** (500 MHz, $CDCl_3$)

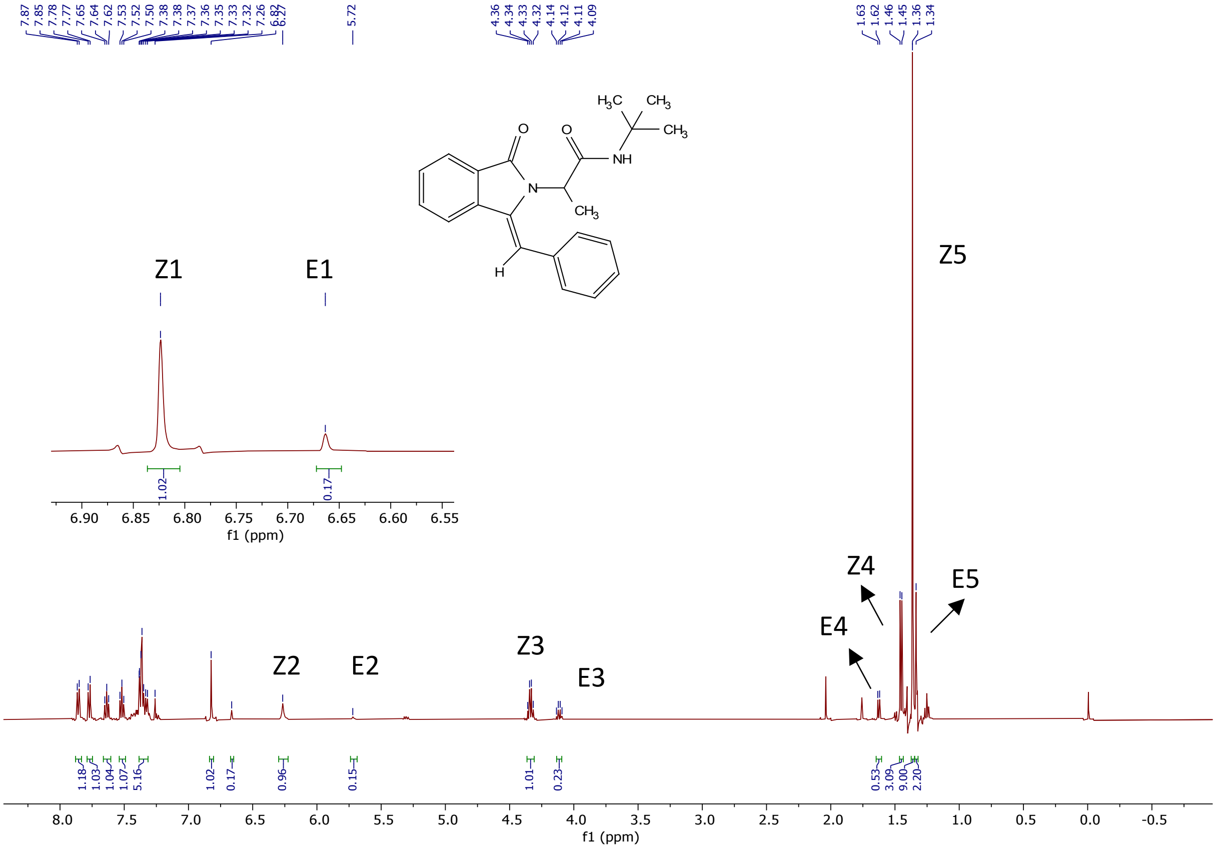

$^{13}C\{^{1}H\}$ NMR spectrum of **5f** (126 MHz, $CDCl_3$)

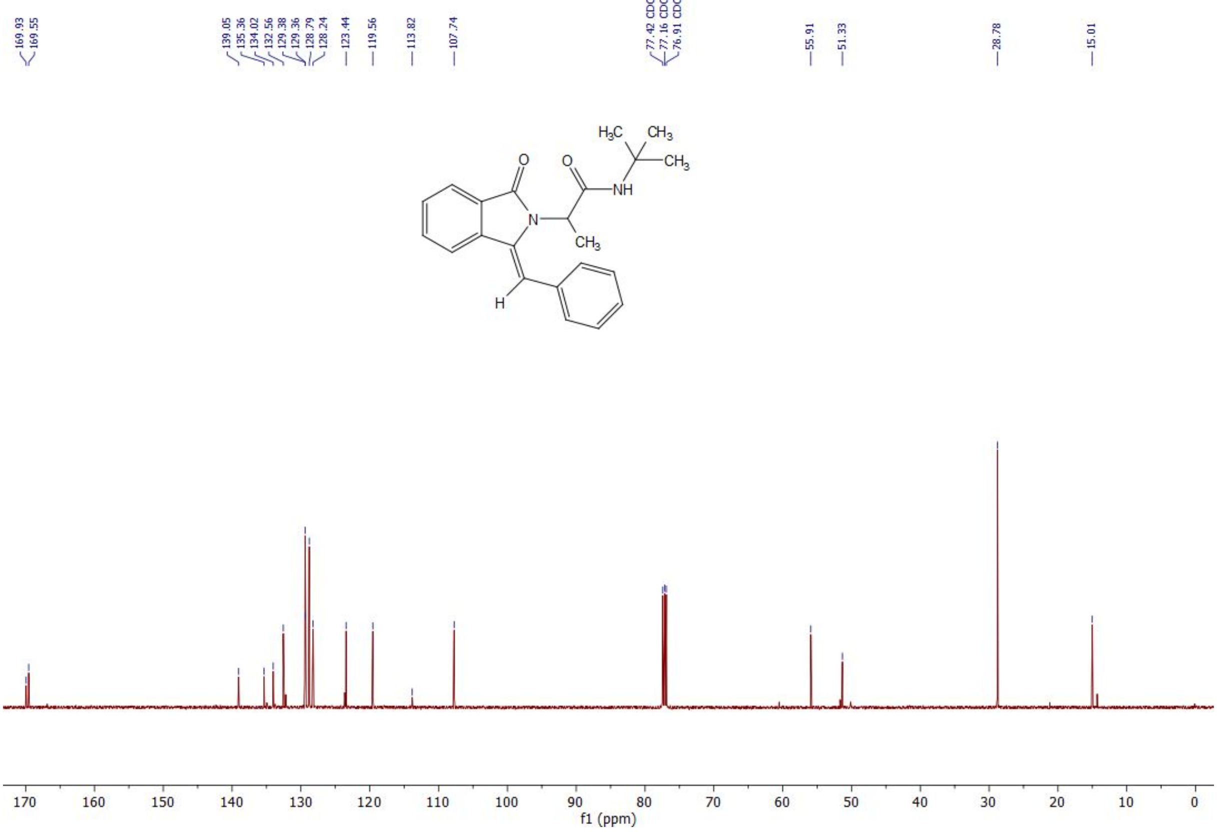

^{1}H NMR spectrum of **5g** (500 MHz, $CDCl_3$)

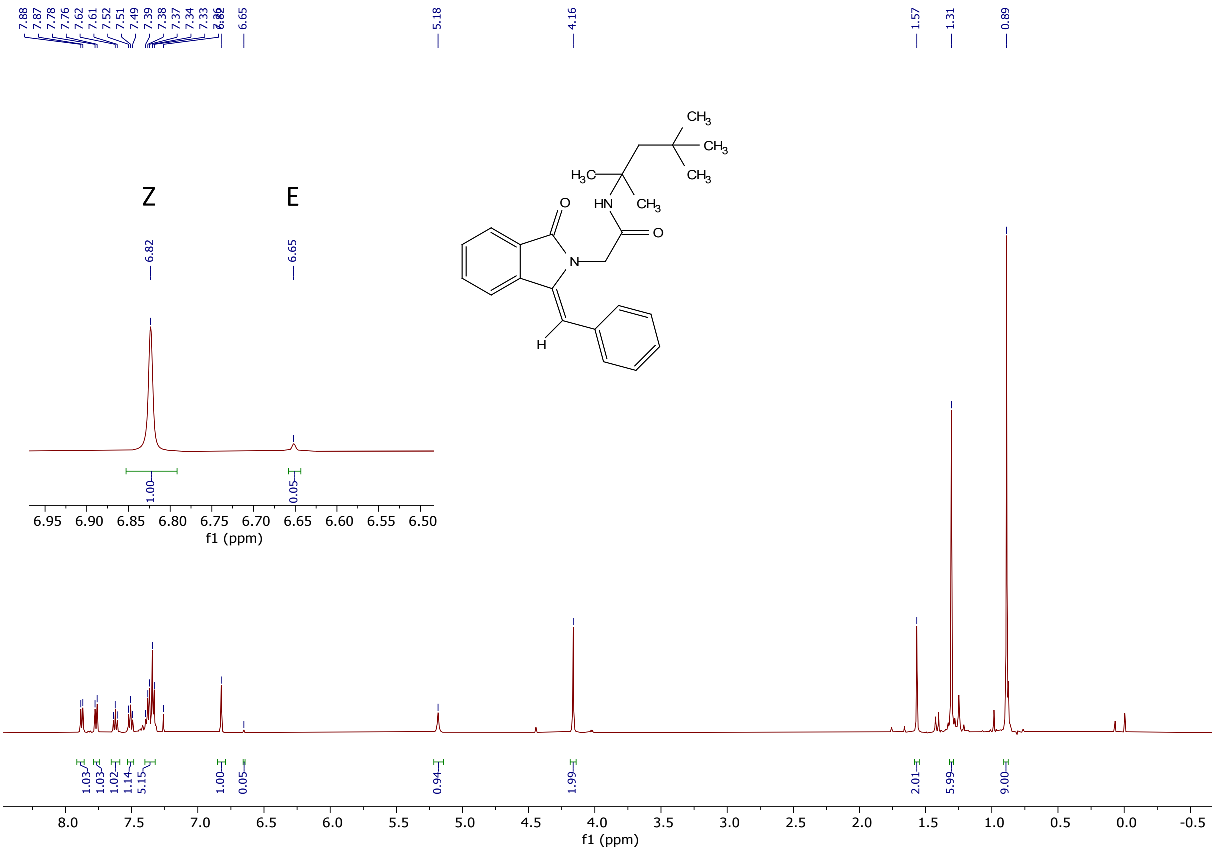

^{13}C{^{1}H} NMR spectrum of **5g** (126 MHz, $CDCl_3$)

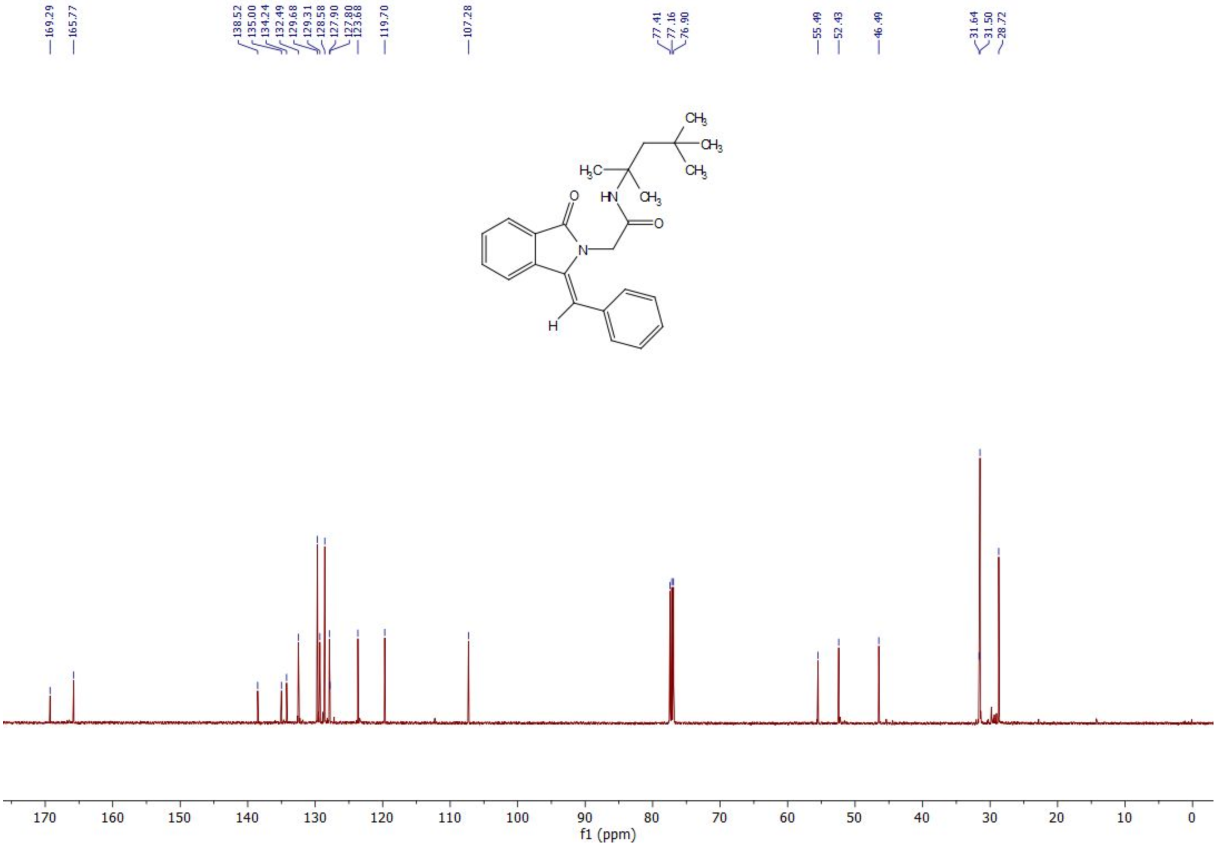

^{1}H NMR spectrum of **5h** (500 MHz, $CDCl_3$)

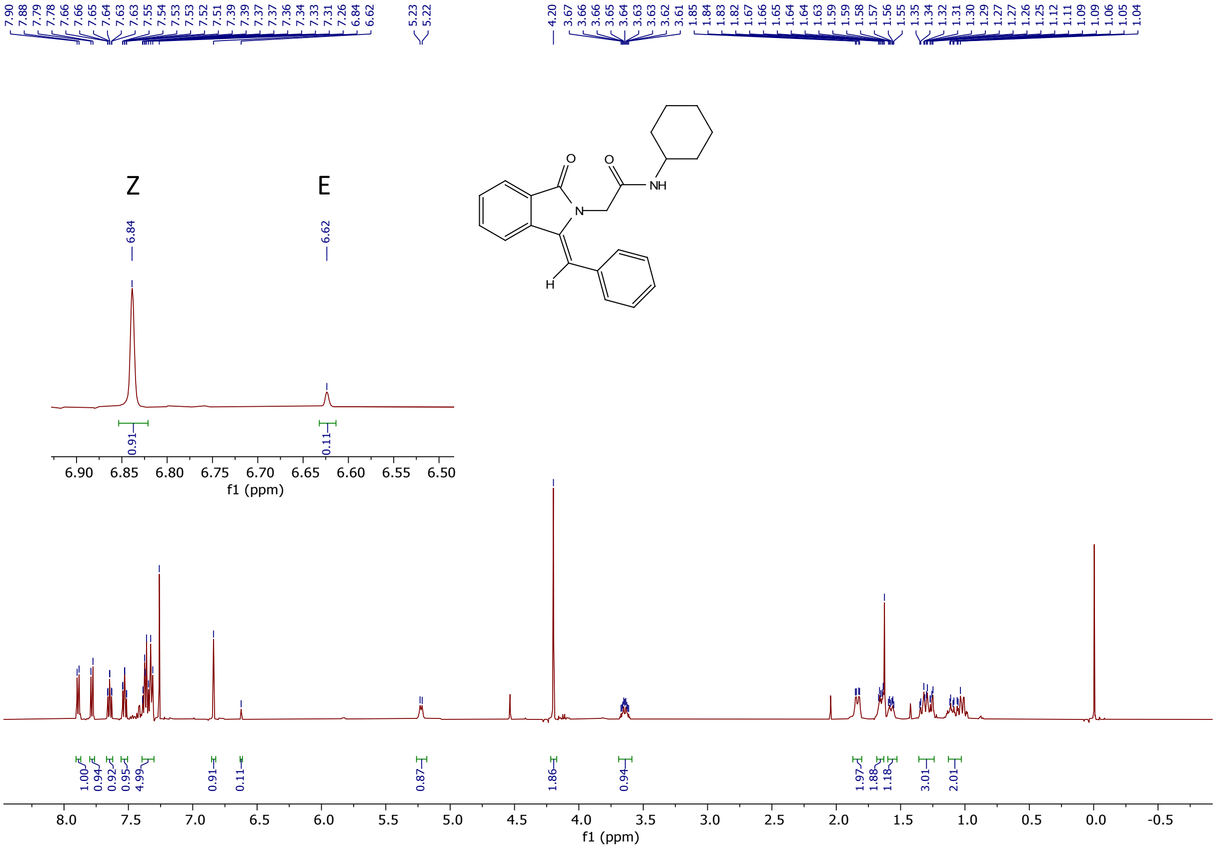

$^{13}C\{^{1}H\}$ NMR spectrum of **5h** (126 MHz, $CDCl_3$)

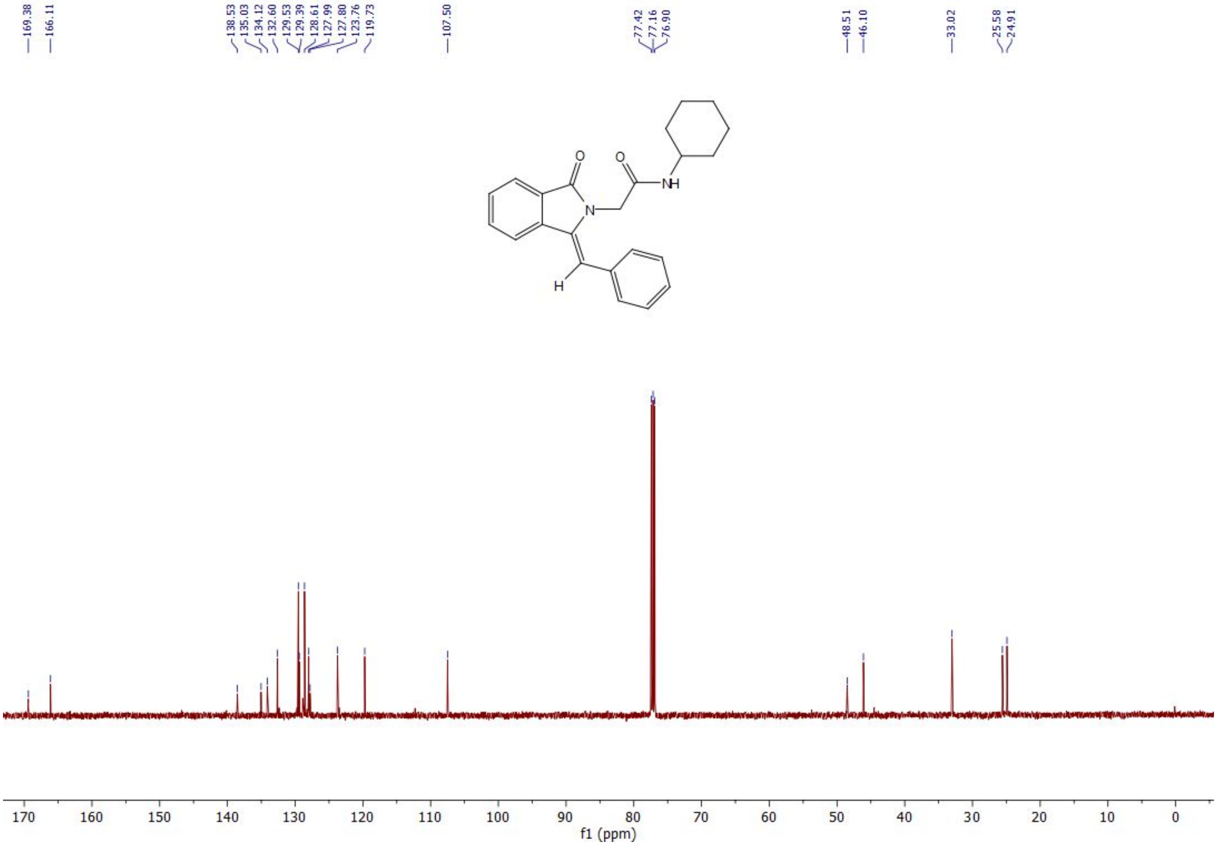

^{1}H NMR spectrum of **5i** (500 MHz, $CDCl_3$)

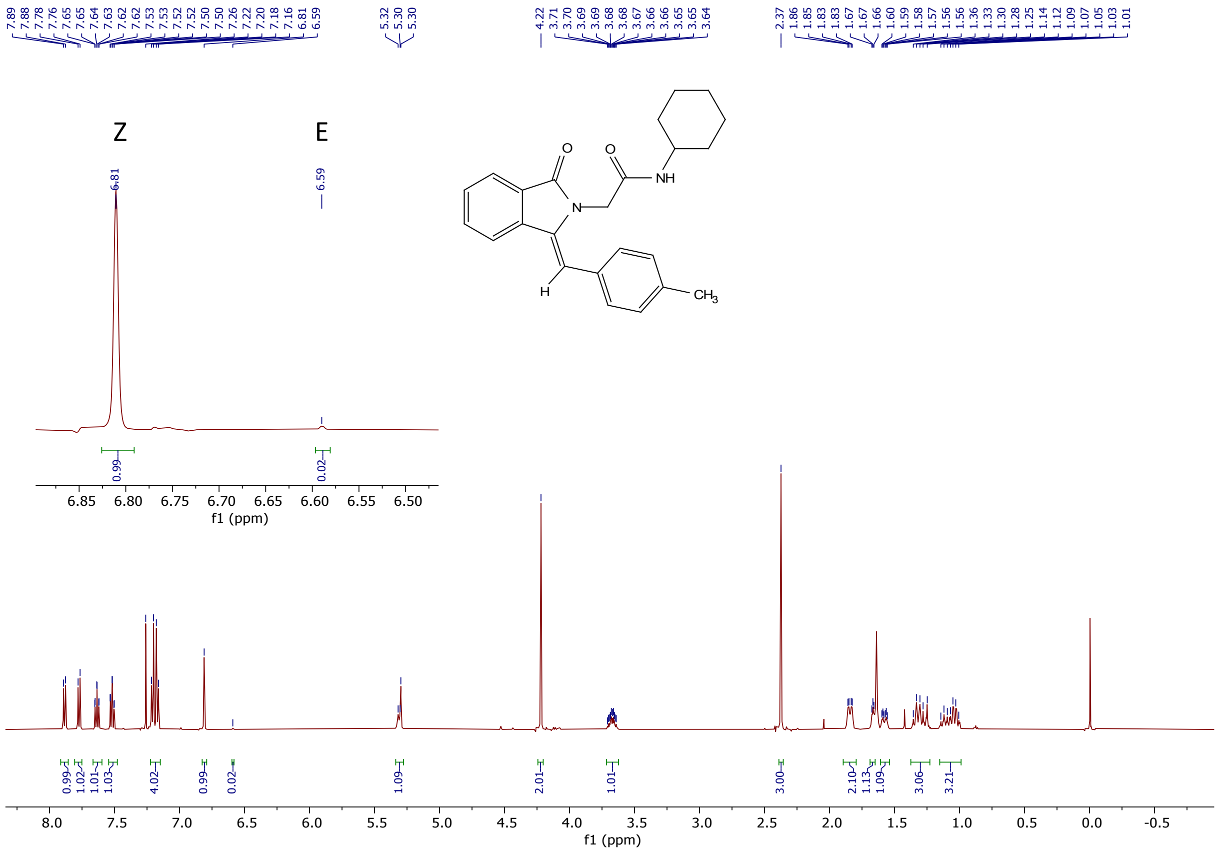

^{13}C{^{1}H} NMR spectrum of **5i** (126 MHz, $CDCl_3$)

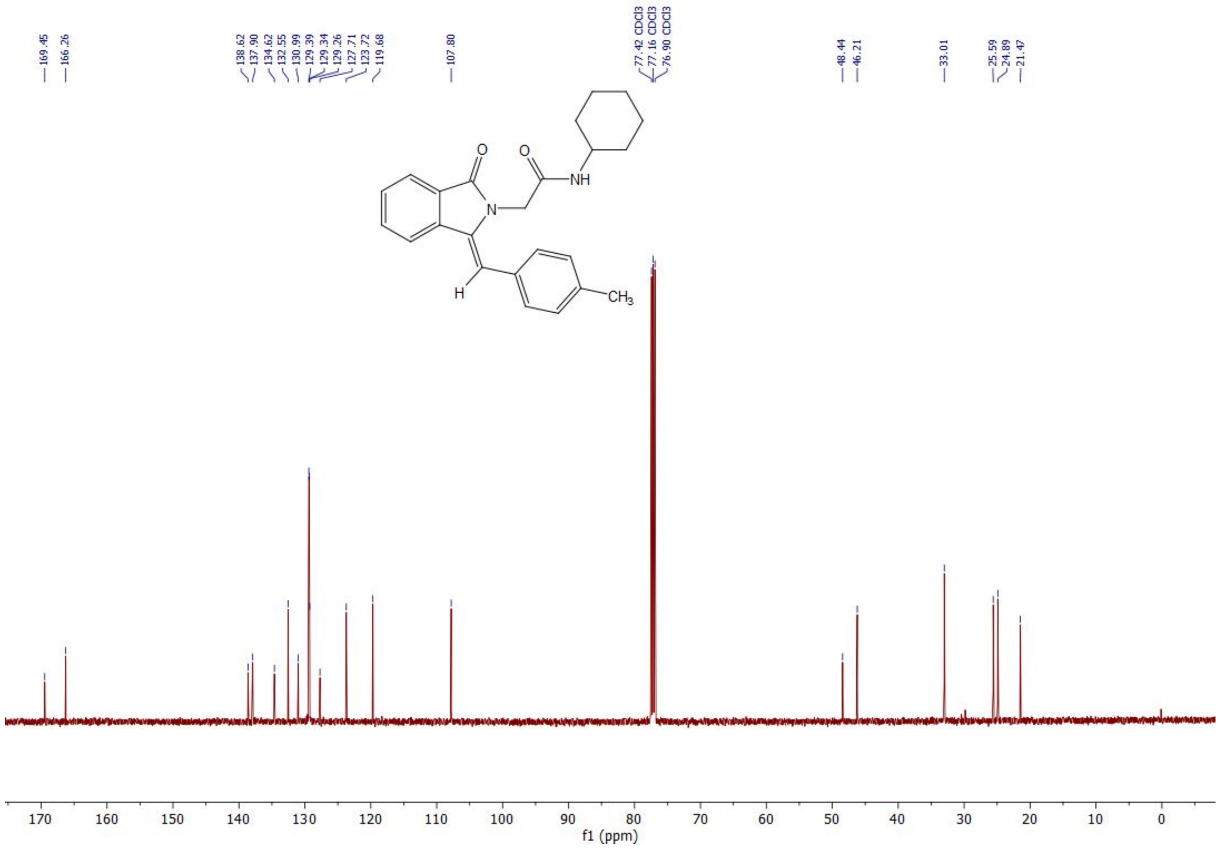

^{1}H NMR spectrum of **5j** (500 MHz, $CDCl_3$)

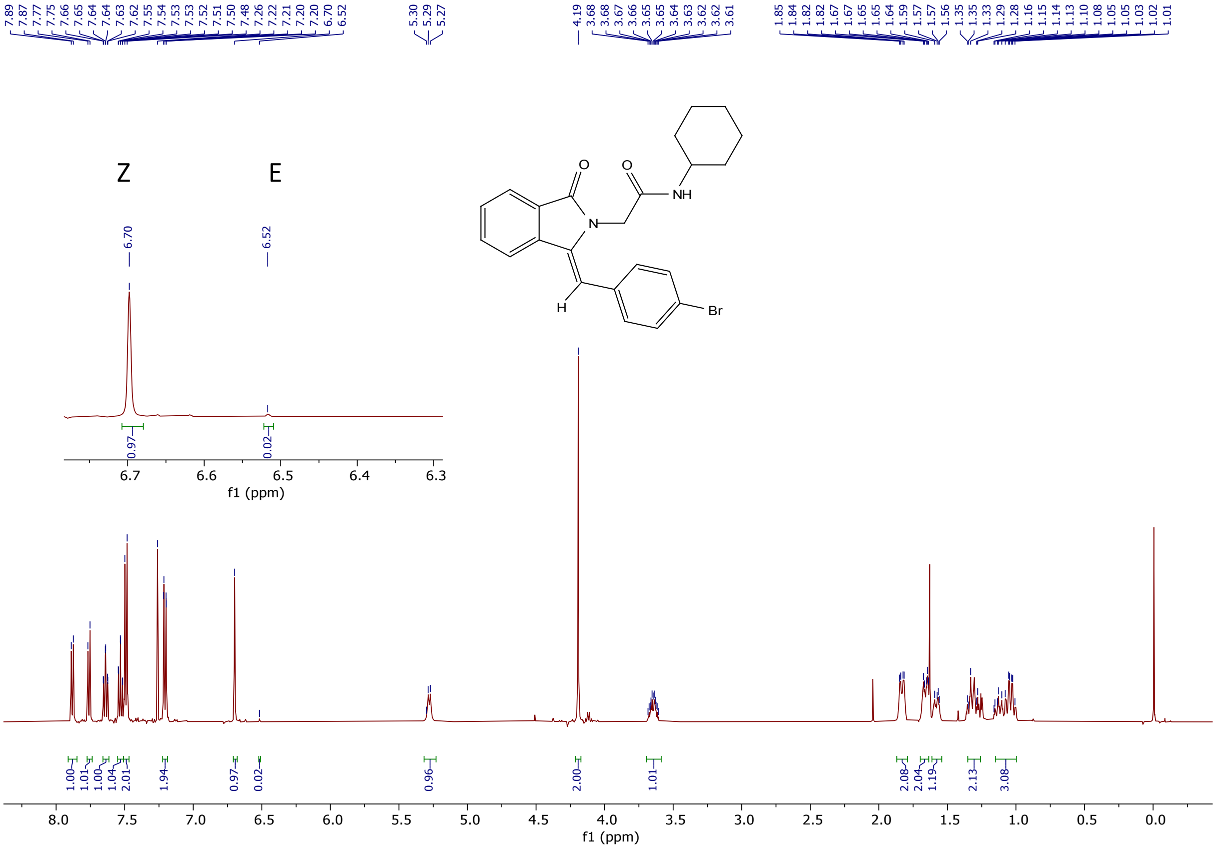

$^{13}C\{^{1}H\}$ NMR spectrum of **5j** (126 MHz, $CDCl_3$)

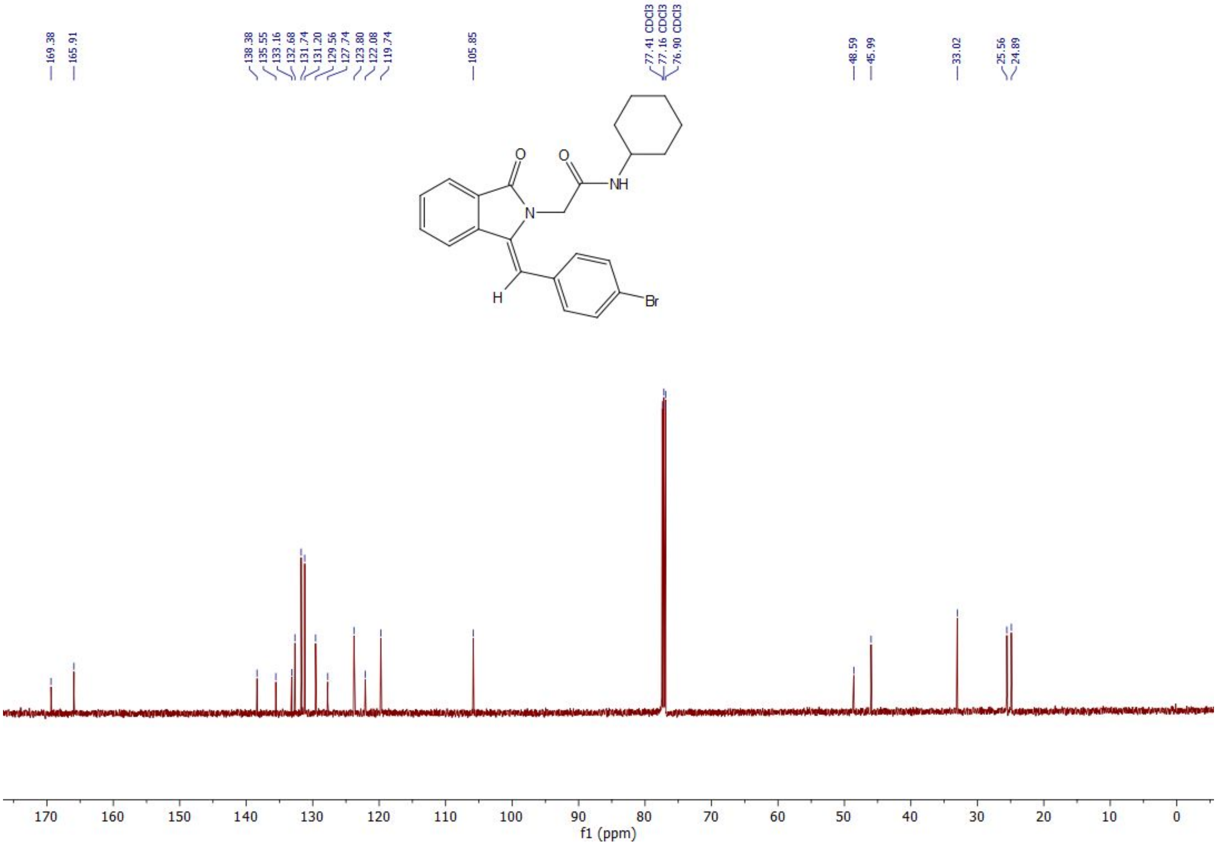

^{1}H NMR spectrum of **5k** (500 MHz, $CDCl_3$)

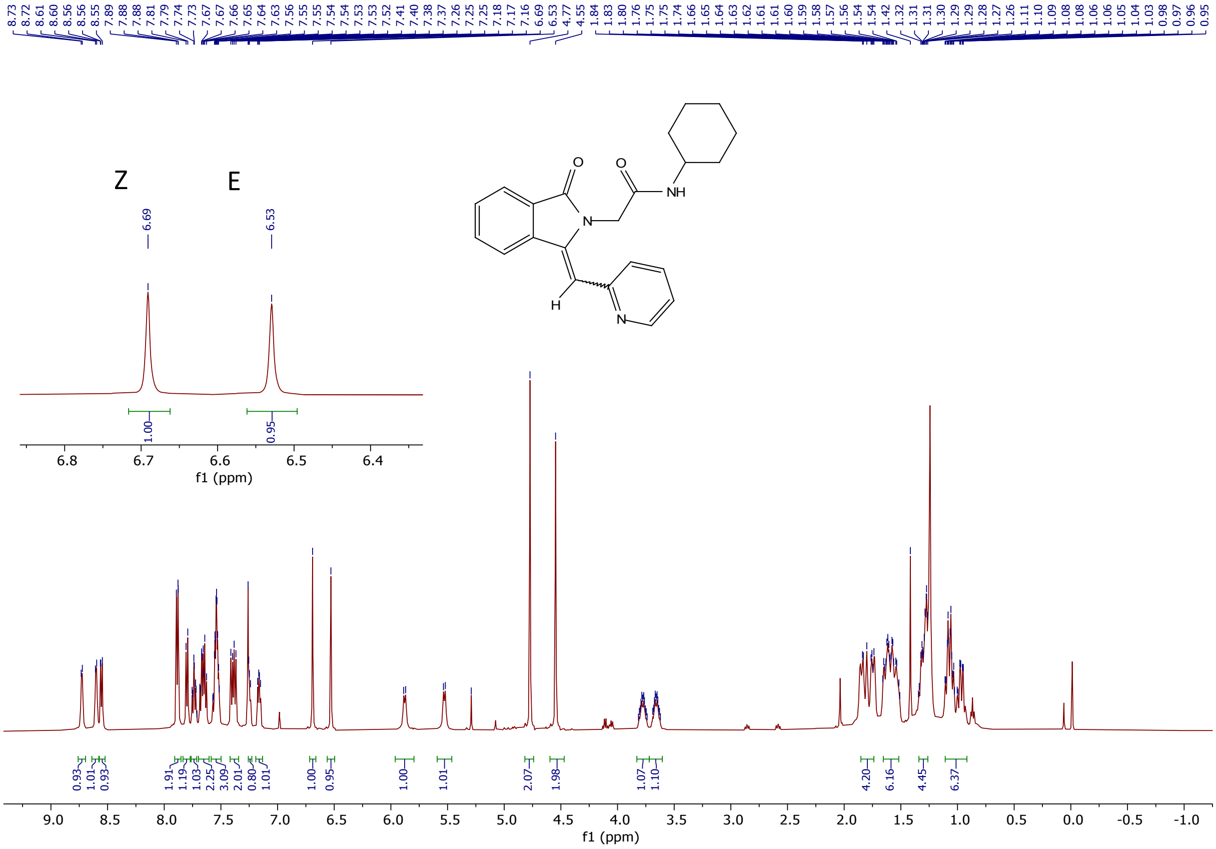

^{13}C{^{1}H} NMR spectrum of **5k** (126 MHz, $CDCl_3$)

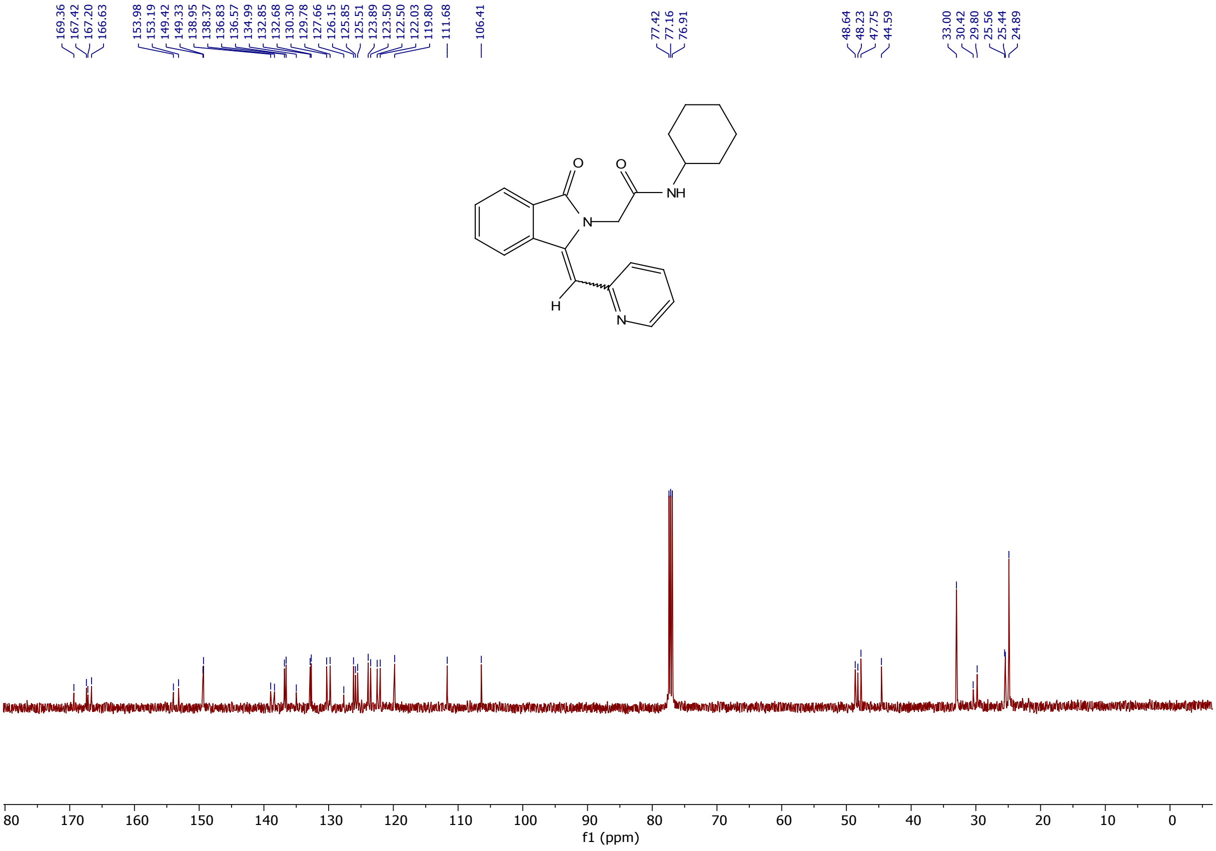

^{1}H NMR spectrum of **5l** (500 MHz, $CDCl_3$)

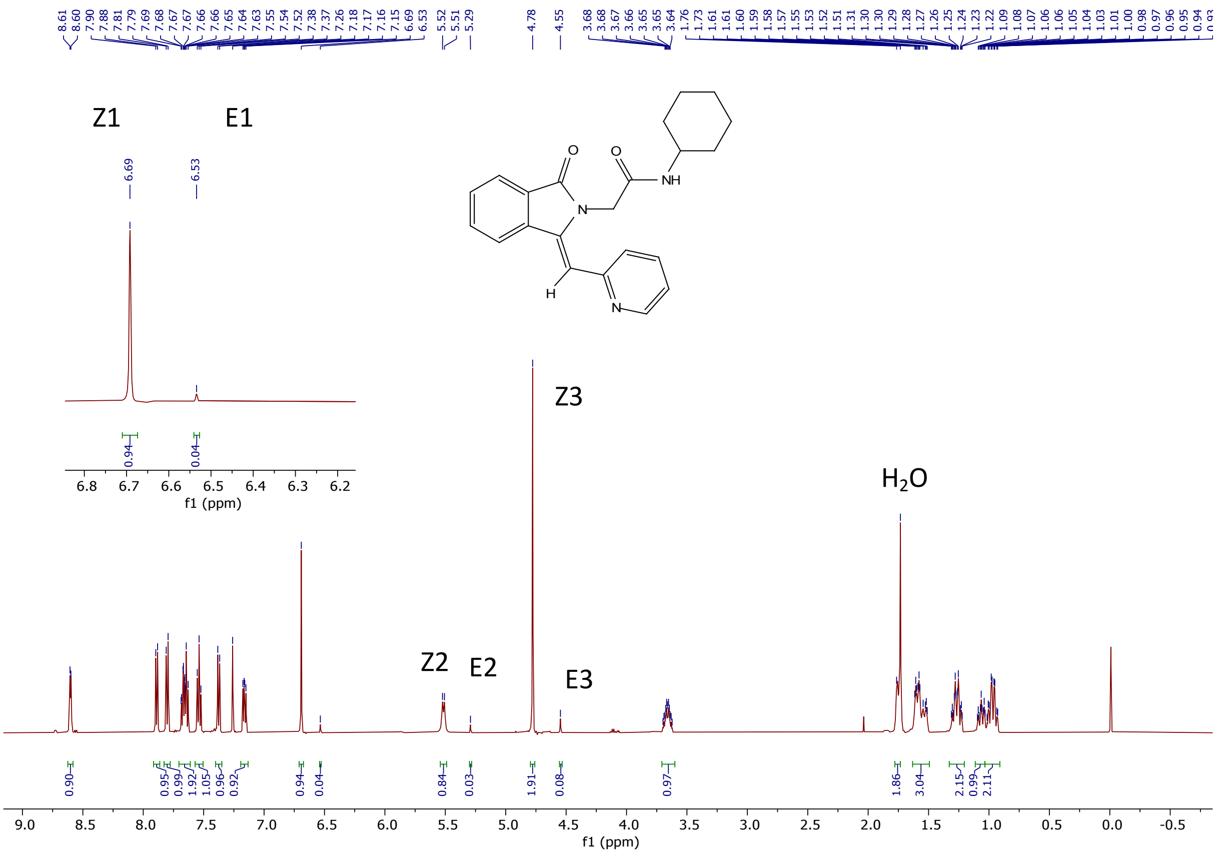

$^{13}C\{^{1}H\}$ NMR spectrum of **5l** (126 MHz, $CDCl_3$)

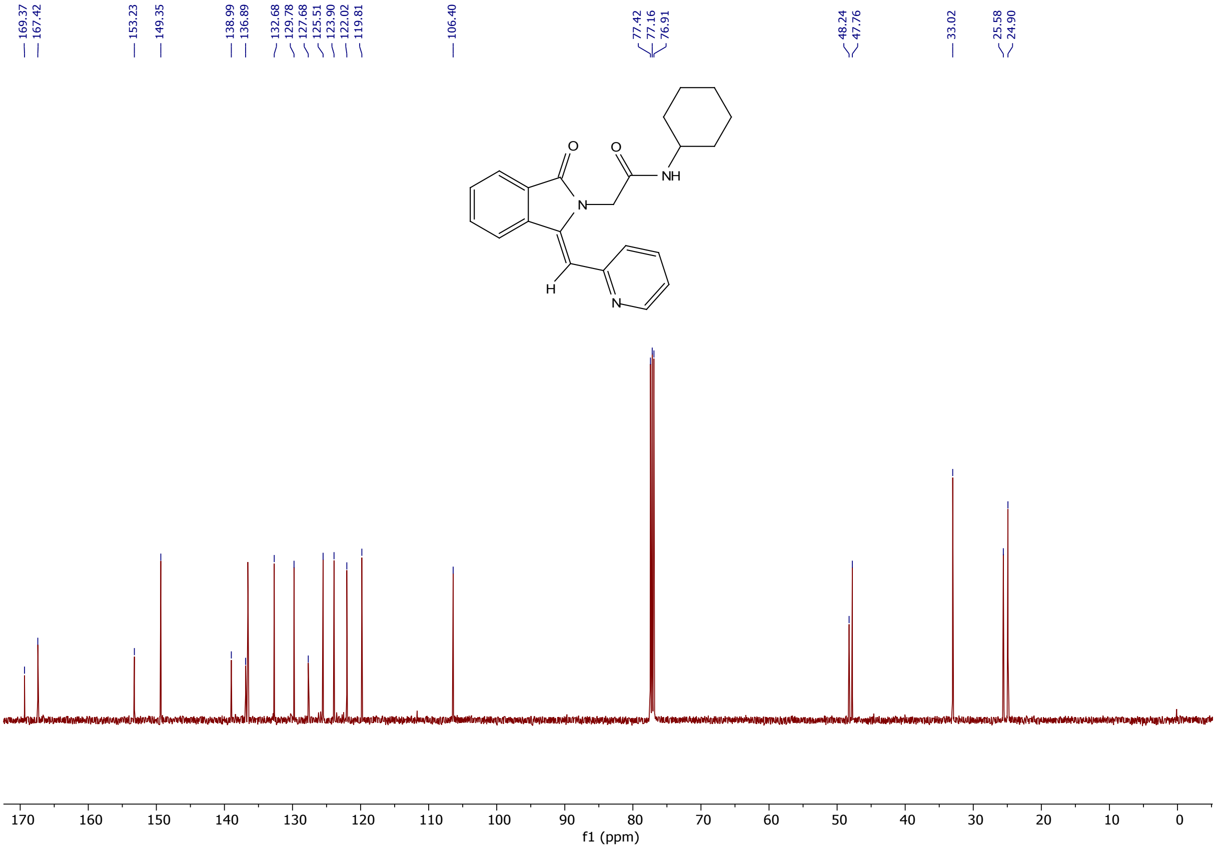

^{1}H NMR spectrum of **5m**(500 MHz, $CDCl_3$)

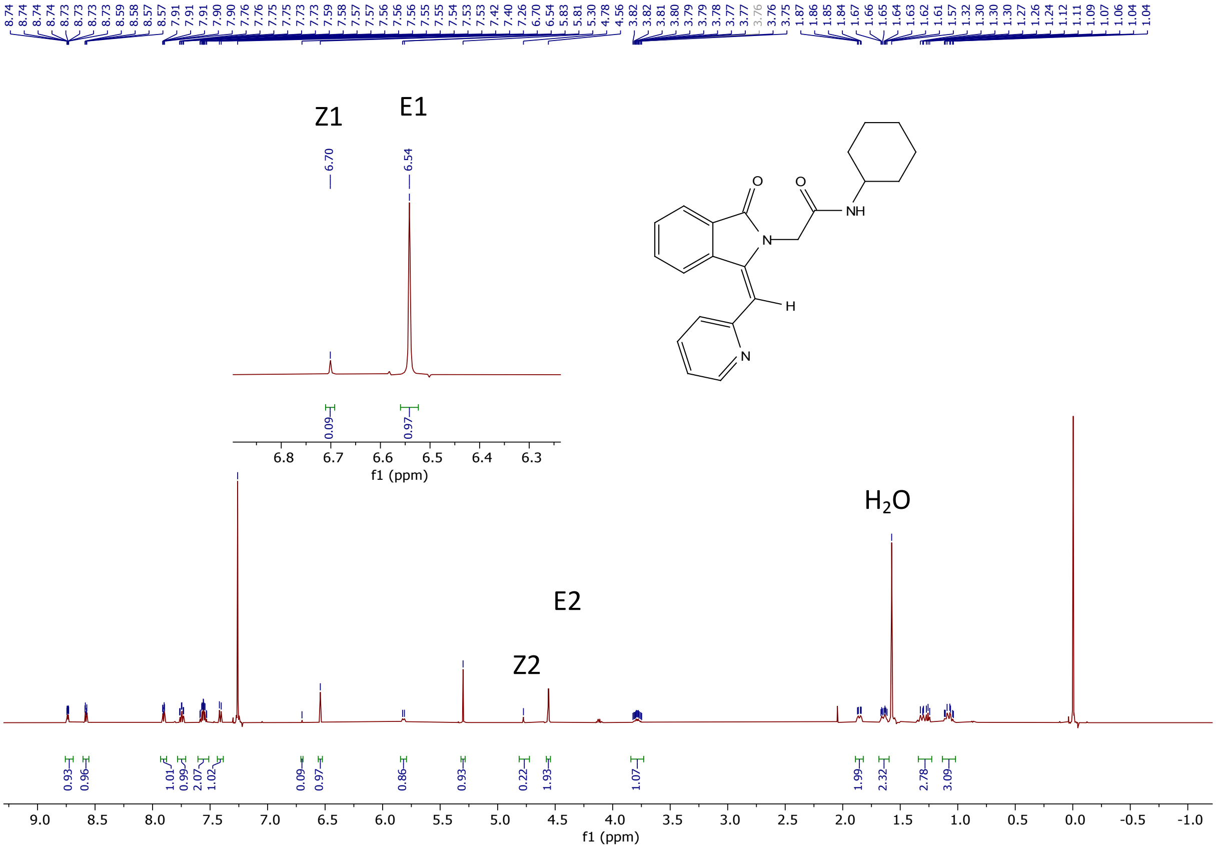

^{13}C{^{1}H} NMR spectrum of **5m**(126 MHz, $CDCl_3$)

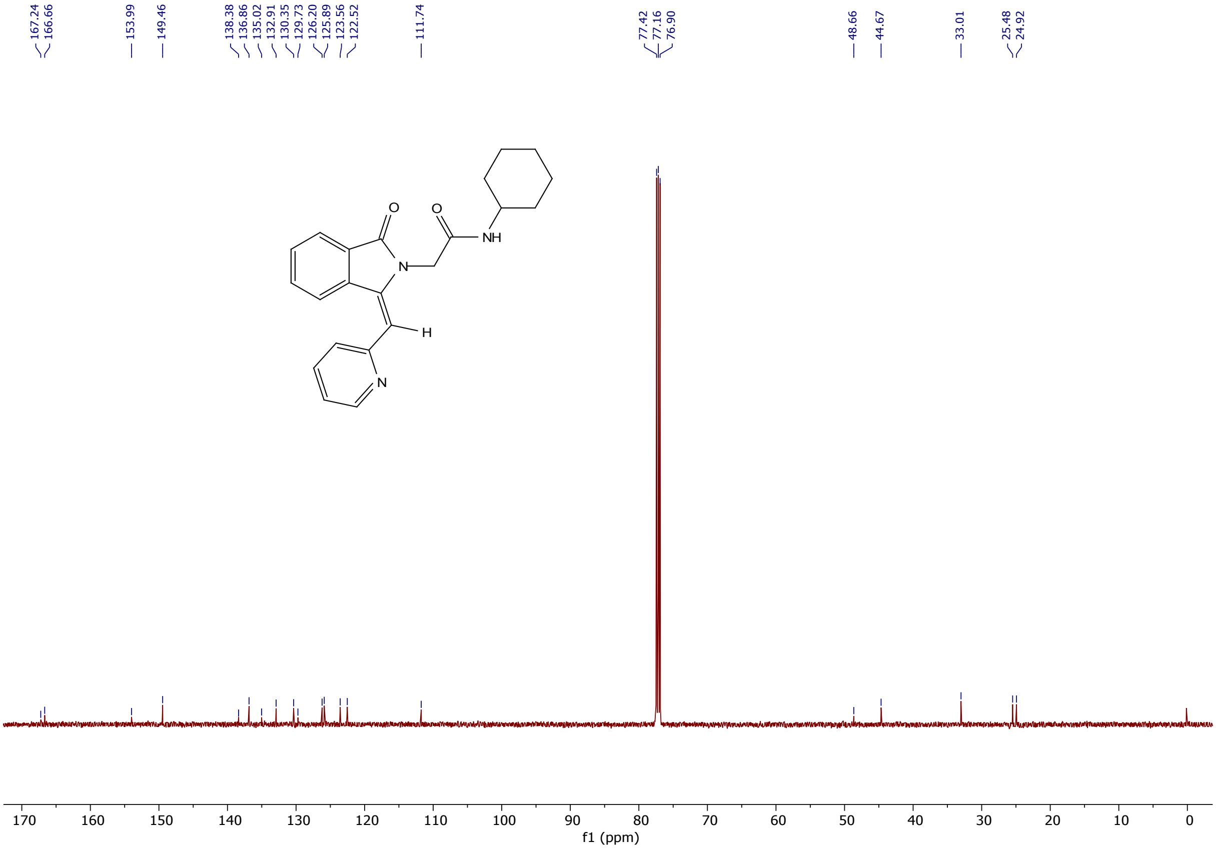

^{1}H NMR spectrum of **5n** (500 MHz, $CDCl_3$)

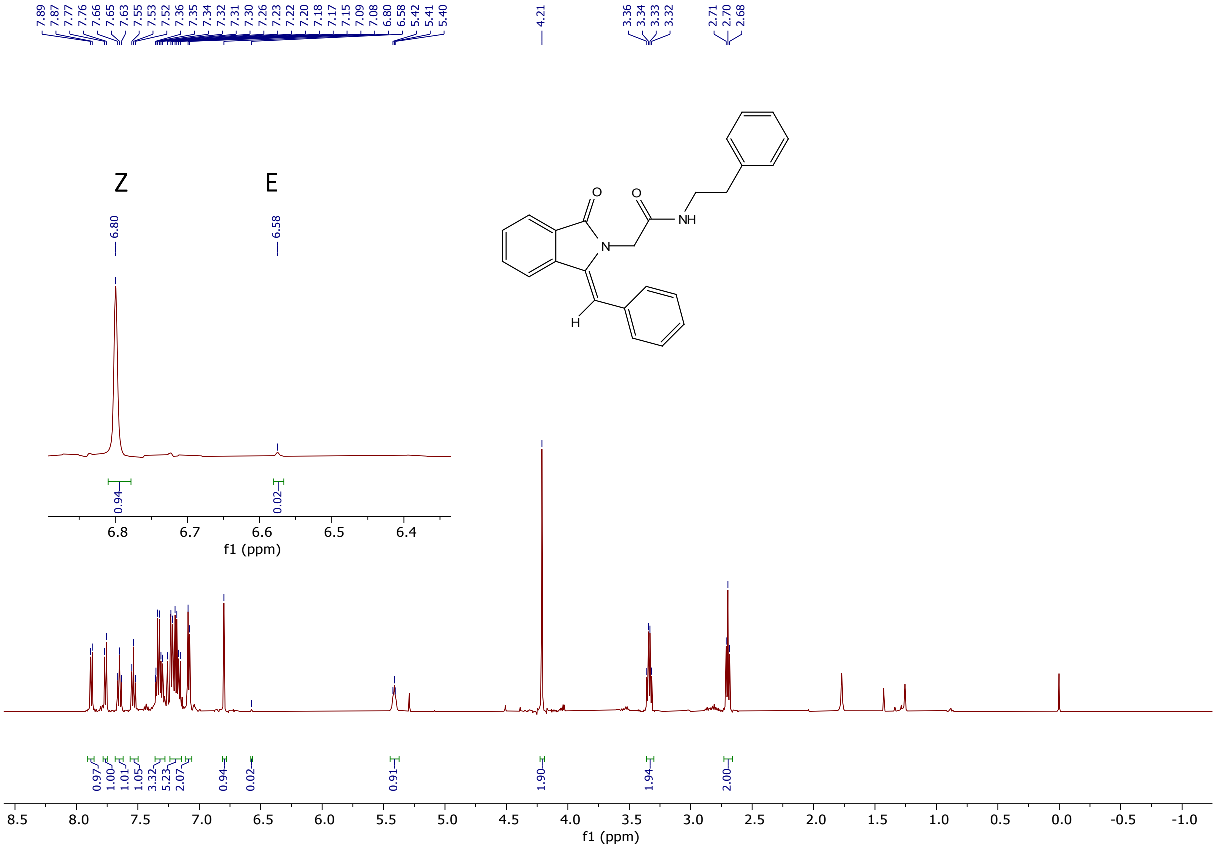

^{13}C{^{1}H} NMR spectrum of **5n** (126 MHz, $CDCl_3$)

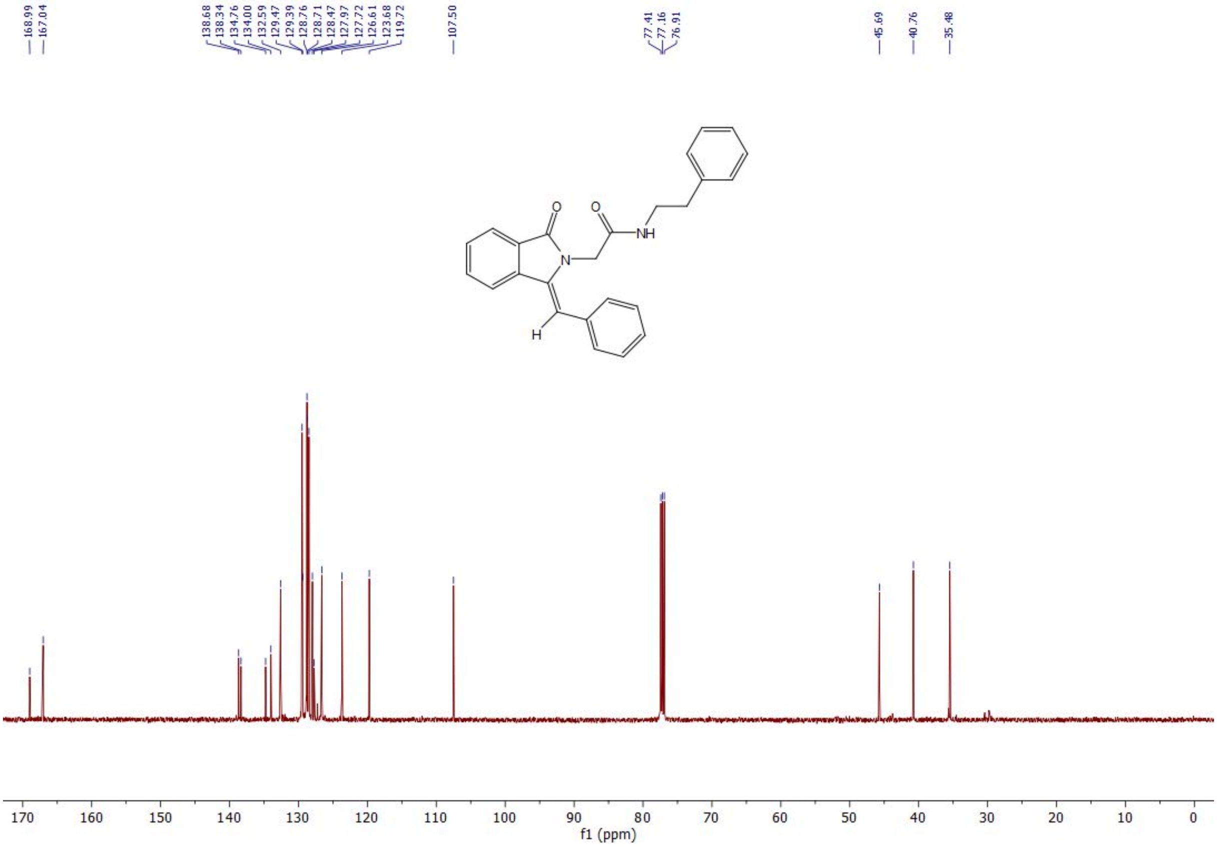

^{1}H NMR spectrum of **5o** (500 MHz, $CDCl_3$)

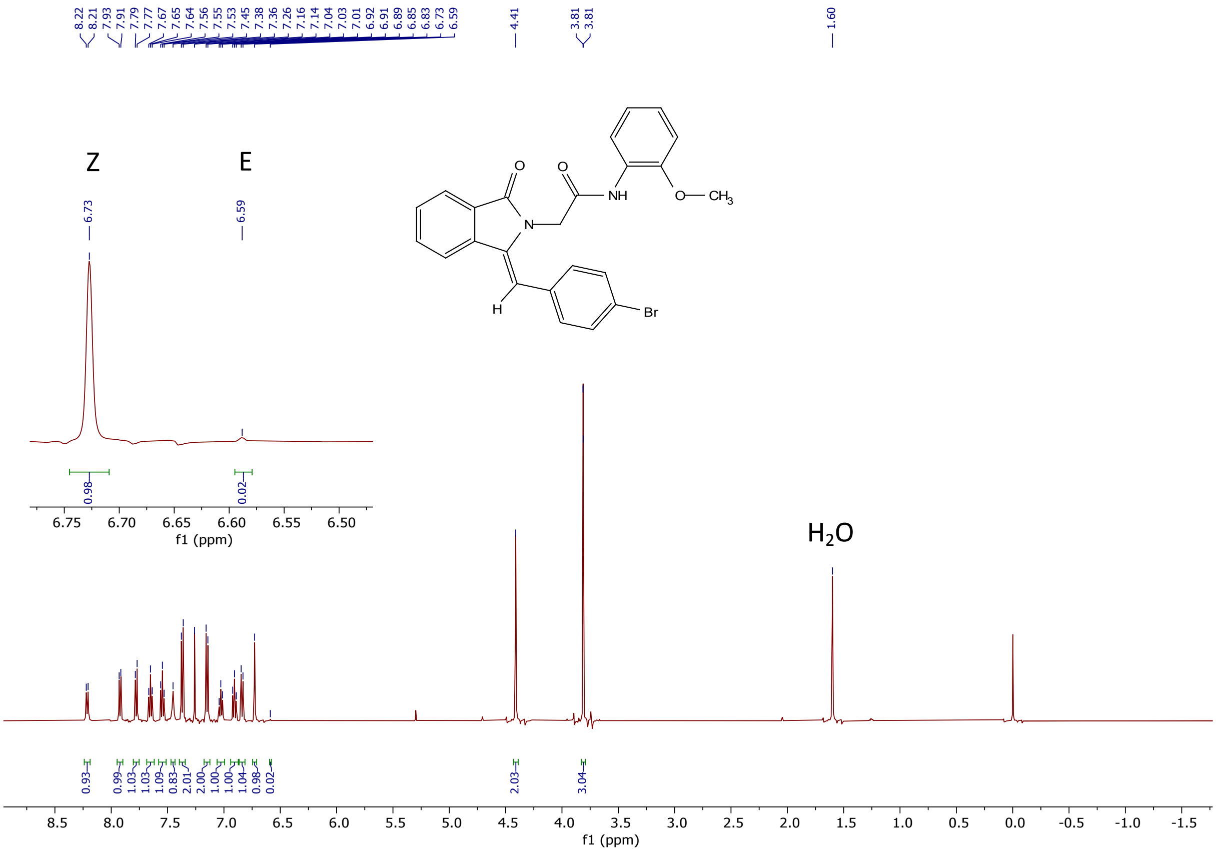

$^{13}C\{^{1}H\}$ NMR spectrum of **5o** (126 MHz, $CDCl_3$)

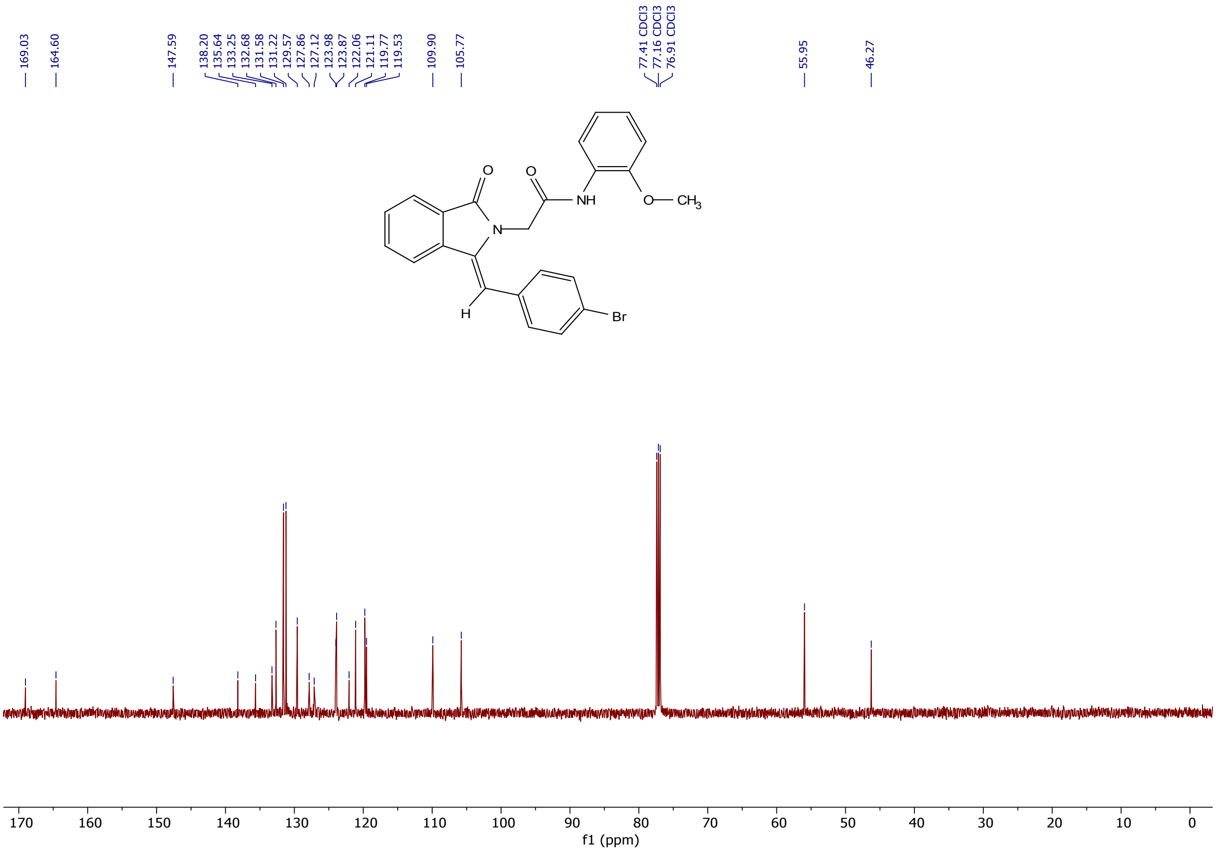

^{1}H NMR spectrum of **6a** (500 MHz, $CDCl_3$)

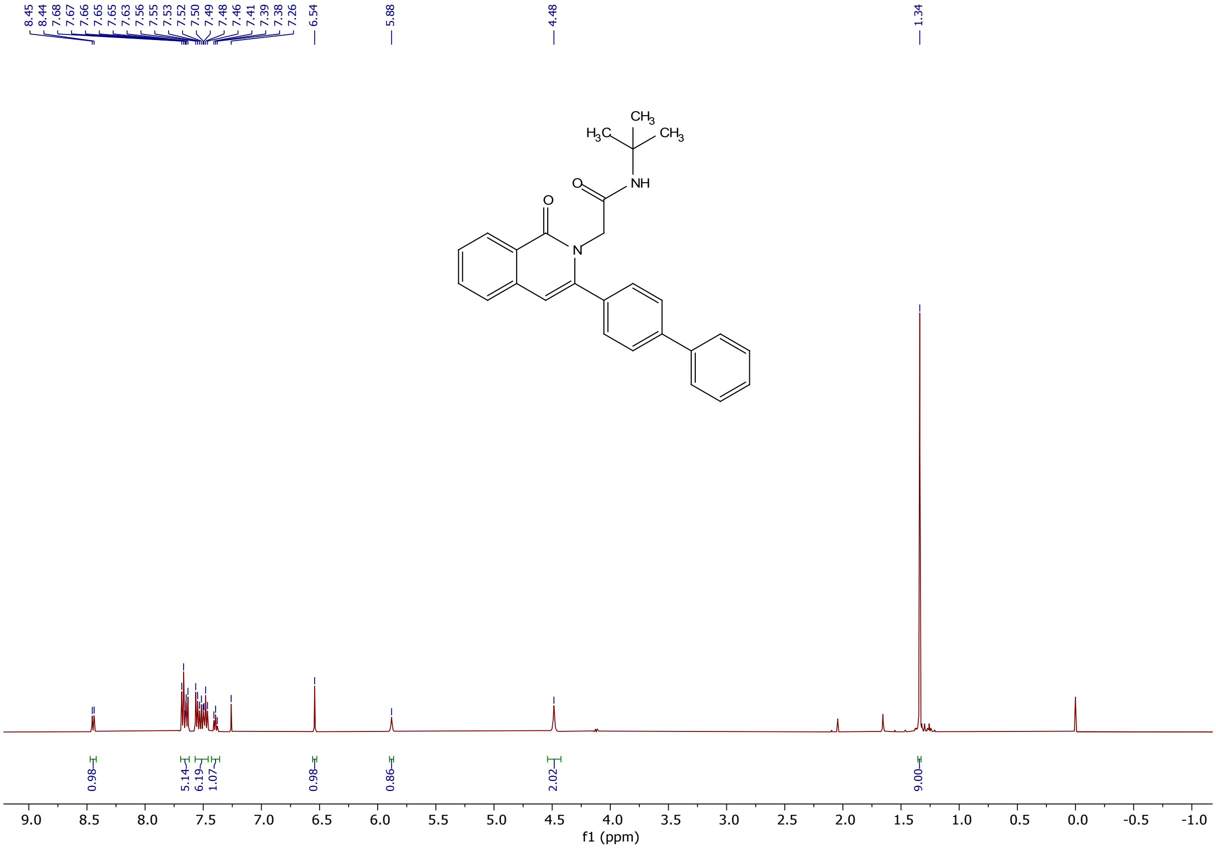

$^{13}C\{^{1}H\}$ NMR spectrum of **6a** (126 MHz, $CDCl_3$)

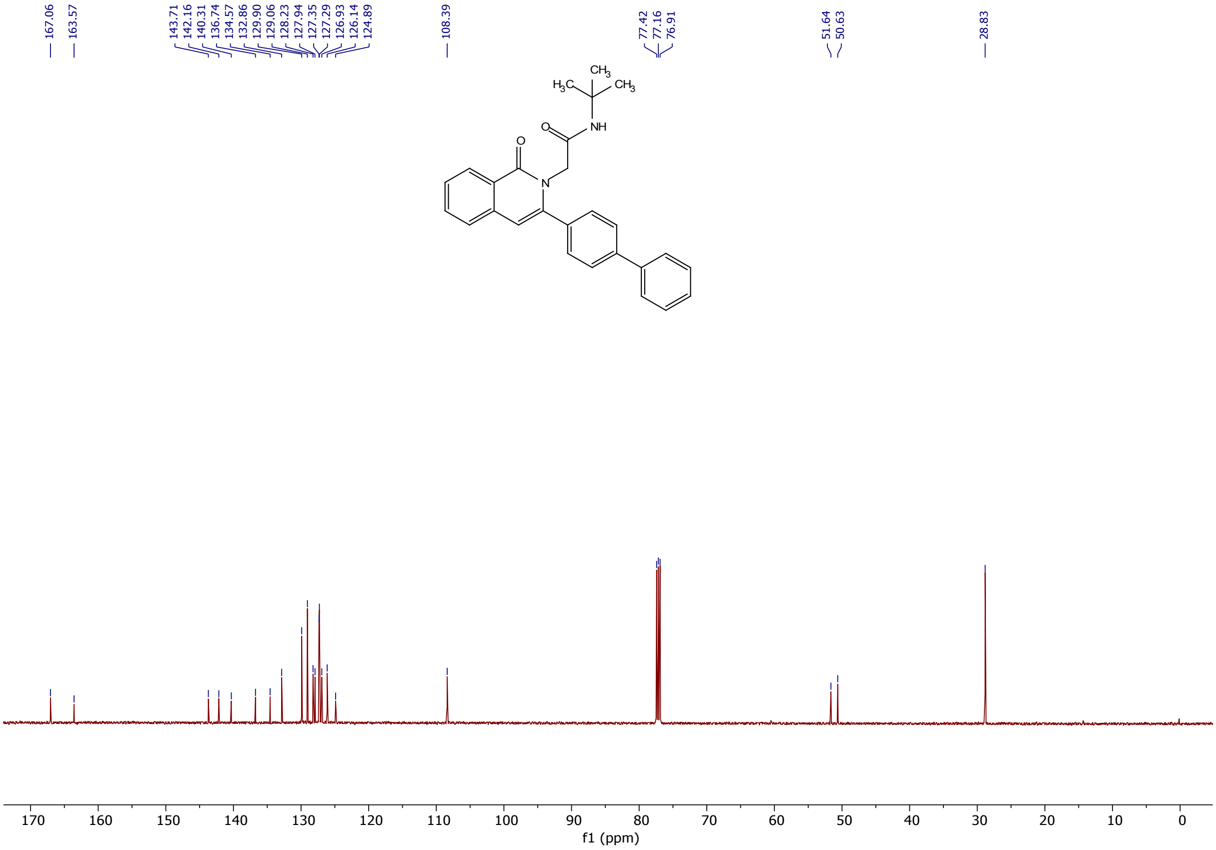

^{1}H NMR spectrum of **6b** (500 MHz, $CDCl_3$)

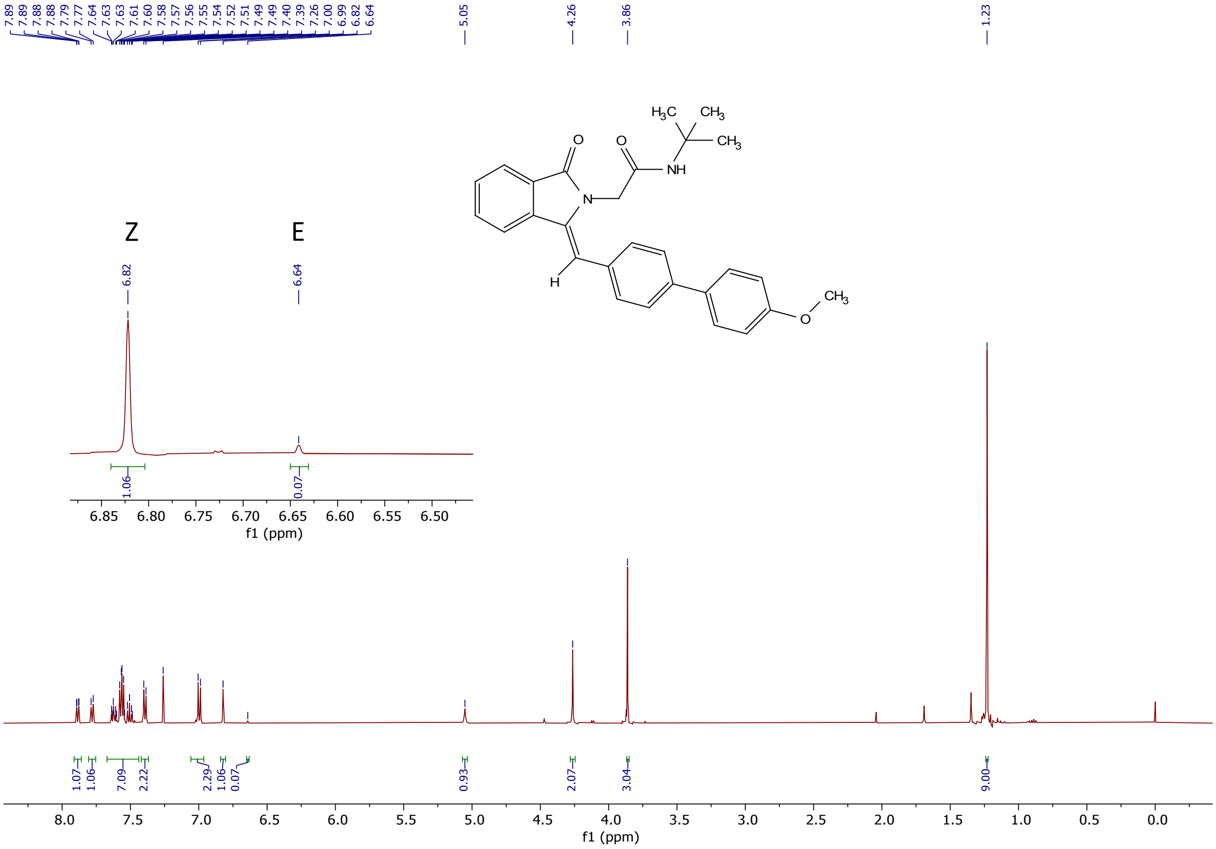

$^{13}C\{^{1}H\}$ NMR spectrum of **6b** (126 MHz, $CDCl_3$)

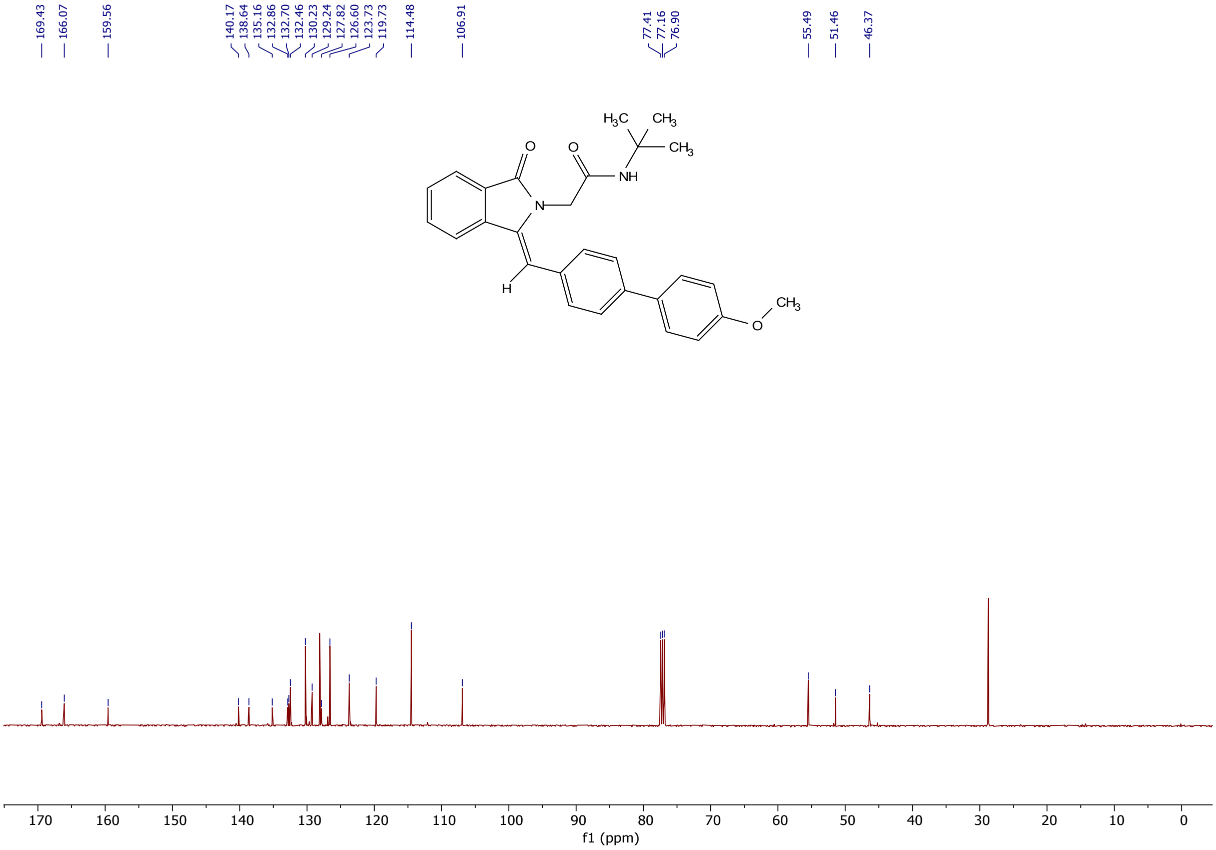

^{1}H NMR spectrum of **6d** (500 MHz, $CDCl_3$)

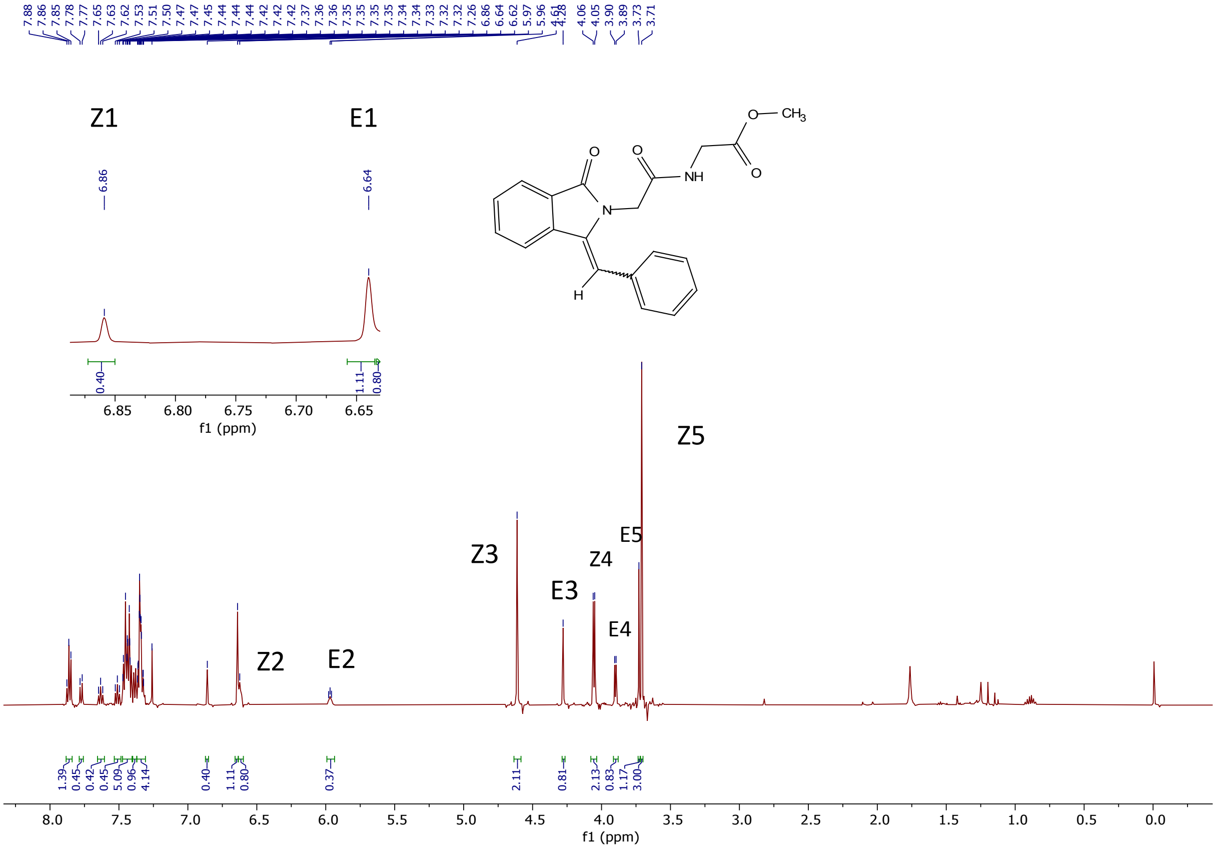

$^{13}C\{^{1}H\}$ NMR spectrum of **6d** (126 MHz, $CDCl_3$)

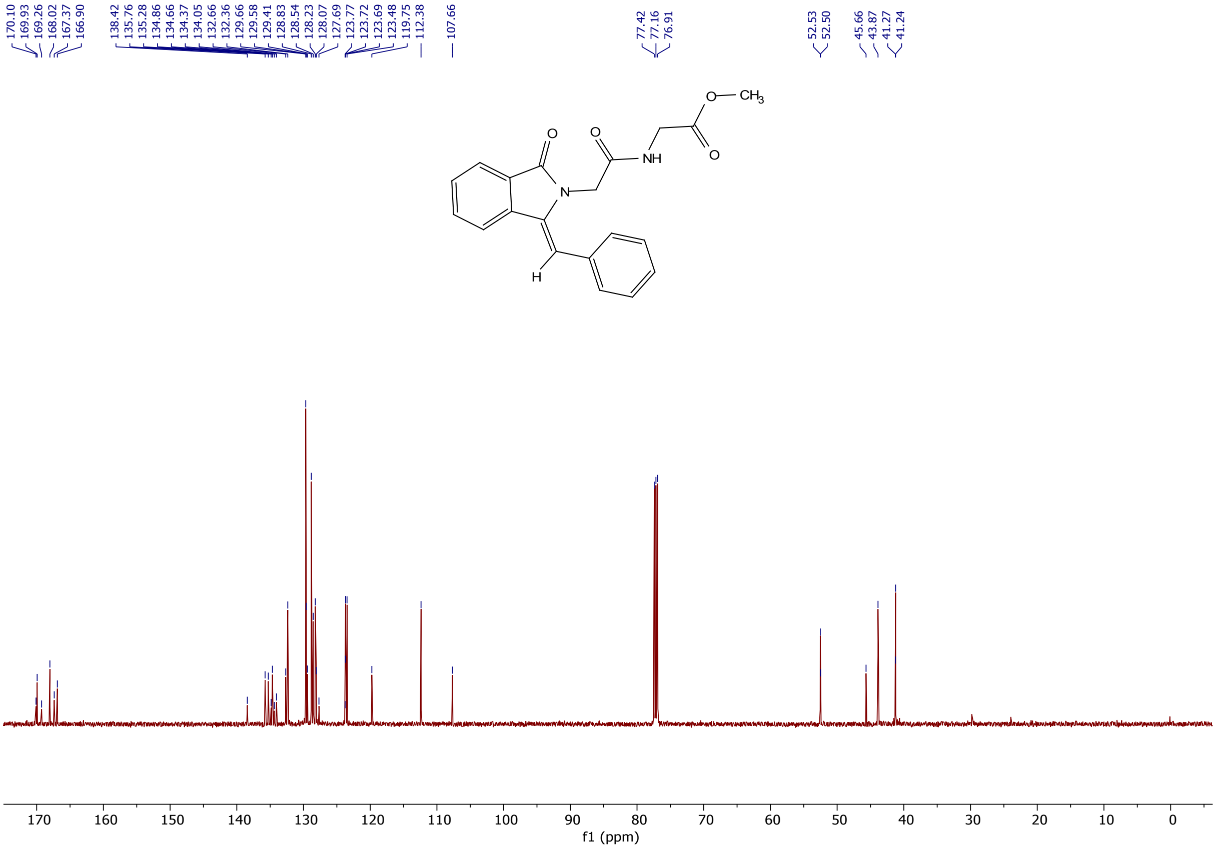

References:

[1] Rigaku-Oxford Diffraction; CrysAlisPro Oxford Diffraction Ltd, Abingdon, England V 1. 171. 36. 2.; (release 27-06-2012 CN) 2006.

[2] Burla M.C., Caliandro R., Carrozzini B. Cascarano G.L., Cuocci C., Giacovazzo C., Mallamo M., Mazzone A., Polidori G., J. Appl. Cryst. 2015, 48 (1), 306–309.

[3] Sheldrick G. M., Acta Cryst. 2008, A64, 112-122.

[4] Farrugia L.J., J. Appl. Cryst. 1999, 32, 837-838.

[5] Macrae C. F., Sovago I., Cottrell S.J., Galek P.T.A., McCabe P., Pidcock E., Platings M., Shields G.P., Stevens J.S., Towler M. and Wood P.A., J. Appl. Cryst. 2020, 53, 226-235.

[6] Wang, Q.; Tuinhof, J.; Mgimpatsang, K. C.; Kurpiewska, K.; Kalinowska-Tluscik, J.; Dömling, A. Copper-Catalyzed Modular Assembly of Polyheterocycles. *J. Org. Chem.* **2020**, *85* (15), 9915-9927.

[7] Li, L.; Wang, M.; Zhang, X.; Jiang, Y.; Ma, D., Assembly of substituted 3-methyleneisoindolin-1-ones via a CuI/L-proline-catalyzed domino reaction process of 2-bromobenzamides and terminal alkynes. *Organic Letters* **2009**, *11* (6), 1309-1312.

[8] GangadharaáChary, R.; VaraáPrasad, K.; ShivaáKumar, K. A simple access to N-(un) substituted isoquinolin-1 (2 H)-ones: unusual formation of regioisomeric isoquinolin-1 (4 H)-ones. *ChemComm.* **2014**, *50* (51), 6797-6800.
